# Non-catalytic role of SETD1A promotes gastric cancer cell proliferation through the E2F4–TAF6 axis in the cell cycle

**DOI:** 10.1038/s41419-025-07976-4

**Published:** 2025-08-23

**Authors:** Meng Ning, Takayuki Hoshii, Takuya Nakagawa, Genki Usui, Shintaro Izumi, Kanako Hayashi, Makoto Matsumoto, Bahityar Rahmutulla, Masaki Fukuyo, Hiroyuki Abe, Tetsuo Ushiku, Atsushi Kaneda

**Affiliations:** 1 Department of Molecular Oncology, Graduate School of Medicine, Chiba University, Chiba-shi, Japan; 2 Department of Otorhinolaryngology, Head and Neck Surgery, Graduate School of Medicine, Chiba University, Chiba-shi, Japan; 3 Health and Disease Omics Center, Chiba University, Chiba-shi, Japan; 4 Department of Pathology, Graduate School of Medicine, The University of Tokyo, Tokyo, Japan

**Keywords:** Oncogenes, Transcription, Cell division

## Abstract

SETD1A is a member of the KMT2 histone H3K4 methyltransferase family of mammalian proteins. Aberrant *SETD1A* expression is associated with a poor prognosis in patients with gastric cancer (GC). We found that the catalytic domain of SETD1A is nonessential for GC cell proliferation, whereas the non-catalytic FLOS domain is essential. The loss of SETD1A commonly reduces the expression of E2F target genes in GC cell lines from the three independent molecular subtypes. A pooled CRISPR screen and cDNA rescue experiment showed that TAF6 acts downstream of SETD1A’s non-catalytic function, which is essential for GC cell proliferation. Both SETD1A and TAF6 are required for G1/S cell cycle progression in GC cells. The mRNA expression of *E2F4* highly correlated with both the *SETD1A* and *TAF6* expression in patients with GC. Notably, E2F4 supported the expression of TAF6 but not that of SETD1A, suggesting that E2F4 serves as a coregulator of SETD1A, which is involved in regulating TAF6. These results demonstrate that the non-canonical roles of SETD1A and its downstream pathways are crucial for cell cycle progression in GC.

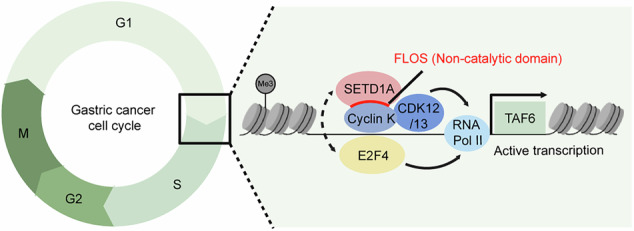

## Introduction

Gastric cancer (GC) has a high incidence worldwide. GC accounts for a large portion of global mortality and has a low survival rate [1]. Therefore, identifying therapeutic targets is crucial for improving prognosis. Epigenetic alterations are frequently observed in GC, particularly DNA methylation within CpG islands of tumor suppressor genes such as *CDKN2A* and *MLH1* [2–4]. The overexpression of epigenetic enzymes that act as transcriptional repressors, such as DNMT1/3 DNA methyltransferases and EZH2 histone methyltransferase (HMT), positively correlates with GC progression [5–7]. In contrast, SETD1A (KMT2F), a histone H3 lysine 4 HMT, is associated with transcriptional activation and promotes tumorigenesis, as well as malignant phenotypes in GC [8, 9].

SETD1A is a member of the KMT2 H3K4 HMT family of mammals [10]. Each HMT KMT2 contains a catalytic SET domain. A comprehensive analysis defined the frequent mutation rates of H3K4 HMTs, including *KMT2A/C/D* in the GC microsatellite instability (MSI) subtype, and genes associated with the cell cycle and DNA repair pathways are upregulated in *KMT2*-mutant GCs [11]. *KMT2C/D* is subject to frequent mutational events in mucinous GC [12]. Notably, KMT2A promotes GC cell migration and invasion [13]. KMT2D promotes GC cell proliferation and suppresses apoptosis [14]. However, the importance of the catalytic activity of each enzyme associated with the SET domain in GC remains unclear.

SETD1A is a well-characterized KMT2 HMT harboring catalytic and non-catalytic functions. The SET domain of SETD1A is essential for embryonic stem (ES) cell differentiation but not for proliferation and self-renewal in ES cells [15, 16]. The N-terminus of SETD1A, which consists of an RNA recognition motif (RRM) and a WDR82 binding motif, promotes the transcription of CpG-island-associated genes in ES cells [17]. In leukemia, the non-catalytic FLOS domain of SETD1A is necessary for cell survival and interacts with Cyclin K, a cofactor of CDK12/13 that phosphorylates RNAP2 [18]. Thus, SETD1A regulates RNAP2 for the active transcriptional elongation of the genes associated with DNA repair and mitochondrial respiration in leukemia cells [19]. However, the roles of SETD1A’s catalytic and non-catalytic domains in GC remain unclear. Understanding the functional roles of SETD1A may lead to the identification of novel therapeutic targets in GC. Here, we found that the non-catalytic domain of SETD1A is crucial for the cell cycle and the regulation of the expression of the general transcription factor TAF6 in GC. SETD1A cooperatively regulates TAF6 expression via the cell-cycle-related transcription factor E2F4. Collectively, our study demonstrates the essential role of SETD1A’s non-catalytic domain in promoting GC cell cycle progression.

## Materials and methods

### Cell lines and culture

The 293T (American Type Culture Collection; ATCC) and Plat-A (gifted by Dr. Kitamura [20]) cells were cultured in Dulbecco’s modified Eagle’s medium (DMEM) supplemented with 10% fetal bovine serum (FBS) and 1% penicillin–streptomycin. AGS (ATCC), SNU719 (Korean Cell Line Bank), and MKN45 (Japanese Cancer Research Resources Cell Bank) cells were cultured in Roswell Park Memorial Institute (RPMI) 1640 medium containing 10% FBS and 1% penicillin–streptomycin. AGS cells were supplemented with nonessential amino acid solution (Life Technologies). Cells were maintained in a humidified incubator at 37 °C and 5% CO_2_.

### Competitive growth assay

Doxycycline (Dox)-inducible Cas9 (iCas9) cell lines were generated through the lentivirus infection of GC cells with the pCW-Cas9 vector (Addgene #50661), followed by the isolation of single-cell-derived clones that highly expressed Cas9 after Dox treatment. iCas9 cells were infected with sgRNA vectors (Tables S1 and S3). The percentage of GFP or tRFP657 was monitored three days after infection using a CytoFLEX Flow Cytometer (Beckman Coulter), and the cells were subsequently treated with Dox. The percentage of GFP or tRFP657 was monitored every 7 days after Dox treatment. sgEmpty and sgRPA3 were used as the negative and positive controls, respectively.

### cDNA rescue experiment

pMSCV-SETD1A-ires-GFP and pLKO5.sgRNA.EFS.tRFP657.ires.Hygro vectors were constructed as described in previous studies [18, 21, 22]. SETD1A expression vectors were transduced into iCas9-AGS cells via retroviral infection. GFP-positive cells were sorted using a SH800 Cell Sorter (Sony), followed by transduction with sgRNA vectors. The competitive growth assay was performed as described in the previous section. tRFP657-expressing cells were treated with 1 mg/mL hygromycin for 7 days to establish cell lines that stably expressed sgRNA.

### CRISPR screening

We designed four sgRNAs (Table S1) for each target using the CRISPick Tool (https://broad.io/crispick), along with 100 non-targeting controls, 10 sgRNAs as negative controls, and 25 sgRNAs targeting essential genes as positive controls. The pooled oligos were synthesized by Twist Bioscience and ligated into the BsmBI-digested sgRNA vector pLKO5.sgRNA.EFS.GFP (Addgene #57822), as described previously [22]. The pooled sgRNA vector was transduced into 1 × 10^6^ iCas9-AGS cells via lentiviral infection. GFP-positive cells were sorted using an SH800 Cell Sorter on day three after infection. The sorted cells (5 × 10^5^) were cultured with Dox and passaged every 3–4 days. Genomic DNA was extracted from 1 × 10^6^ sorted cells on days 0 and 14 after Dox treatment using a NucleoSpin Tissue Kit (Takara Bio). sgRNA sequences were amplified, and the DNA library was prepared and sequenced on a NovaSeq 6000 (Illumina), as described previously [22]. Data were analyzed using PoolQ (3.3.2). We applied a threshold of Log_2_ FC < −1 (day 14 vs day 0) and *P* < 0.05, selecting sensitive genes that had more than two effective sgRNAs.

### TAF6 overexpression

Full-length human TAF6 was cloned into the pLEX305.degTAG.BSD vector. The construct was transduced into 1 × 10^6^ iCas9-AGS cells through lentiviral infection. Infected cells were selected with 10 µg/mL blasticidin for 7 days to establish cells that stably expressed TAF6.

### Western blot

Western blotting was performed as previously described [19]. Blots were incubated with primary antibodies (Table S3) at 4 °C overnight. All primary antibodies were diluted 200–5000 times with 5% skim milk in TBS-T or Can Get Signal Immunoreaction Enhancer Solution (TOYOBO). The immunocomplexes were visualized using Amersham ECL Prime (Cytiva). Signals were detected using ChemiDoc Touch MP (Bio-Rad). Full and uncropped western blotting images were shown in “Supplemental Material_Original Western Blots”.

### ChIP

The ChIP assay was performed as described previously [19, 22]. Appropriate amounts of antibodies (Table S3) were added to chromatin and incubated overnight at 4 °C. Spike-in chromatin and spike-in antibodies (Table S3) were used to normalize the SETD1A ChIP signal. For ChIP-qPCR, DNA was quantified using SYBR Green Real-time PCR with specific primers (Table S2) and NEB Taq polymerase (NEB) using a CFX96 Real-Time PCR detection system (Bio-Rad). For ChIP-seq, the libraries were prepared using a KAPA Hyper Prep Kit (KAPA Biosystems) and sequenced using Illumina NextSeq 500 or NovaSeq 6000 platforms. Data were mapped to the University of California, Santa Cruz Human Genome Assembly (hg19) using Bowtie2, and duplicate reads were removed using Picard tools. Peaks were called, and genes were annotated using HOMER software. Heatmaps and plot profiles of ChIP-seq data were generated using DeepTools.

### RNA quantification

Total RNA was extracted using an RNeasy Mini Kit (Qiagen). cDNA synthesis and RT-qPCR were performed using gene-specific primers (Table S2), as previously described [21]. The RNA-seq libraries were prepared using a TruSeq Stranded mRNA Sample Prep Kit (Illumina). The libraries were sequenced as described above. Data were analyzed using HISAT2 and Cufflinks software. Genes of interest were analyzed using the Enrichr tool.

### Cell cycle and apoptosis

The cell cycle was analyzed using a Click-iT EdU Flow Cytometry Assay Kit (Thermo Fisher Scientific) and a CytoFLEX Flow Cytometer, as previously described [19]. Cells were incubated with a medium containing 10 μM EdU for 2 h at 37 °C. For DNA content analysis, cells were fixed with 100% cold ethanol and incubated with 7AAD and RNase A for 30 min. The stained cells were analyzed using a CytoFLEX Flow Cytometer, and the 7AAD^hi^ population was identified as S/G2/M-phase cells. For the apoptosis assay, cells were incubated with Annexin V APC and 7AAD as previously described [19]. Stained cells were analyzed using a CytoFLEX Flow Cytometer.

### Immunohistochemistry (IHC)

Clinical GC tissue samples were obtained from seven patients at the University of Tokyo Hospital, Japan. Tissue blocks were cut and subjected to IHC analysis, as previously described [23]. For quantitative analysis of immunostaining, *H*-scores were calculated using QuPath software (version 0.5.1, 64-bit) [24] by an experienced board-certified pathologist (Fig. S7). First, three anatomical regions were manually annotated on each IHC-stained image: (1) the tumor region, (2) the foveolar and (3) the isthmus region of the normal gastric mucosa. Cell segmentation was initially performed using the “Cell Detection” function. Based on pathologist-provided ground truth, an object classifier was then trained using the Random Trees (RTrees) algorithm to label each detected cell. In the tumor region, cells were classified into tumor cells, stromal cells, or immune cells; in the normal mucosa, cells were classified into normal epithelial cells or immune cells. Subsequently, the “Positive Cell Detection” function was applied to assign immunostaining intensity scores based on the nuclear DAB optical density mean. Thresholds were set at 0.2, 0.4, and 0.6, corresponding to scores of 0 (negative), 1 (weak), 2 (moderate), and 3 (strong). For *H*-score calculation, only tumor cells in the tumor region and normal epithelial cells in the foveolar and isthmus regions were used to ensure cell-type-specific quantification of immunostaining intensity. The *H*-scores were calculated as (1 × % of cells with score 1) + (2 × % of cells with score 2) + (3 × % of cells with score 3). The Ethical Review Committee of the University of Tokyo Hospital approved the use of clinical specimens (approval number: G3521-(21)). Informed consent was obtained from all participants.

### CD44 staining

A total of 1 × 10^5^ cells were stained in 200× diluted anti-CD44-PE antibody (BioLegend) for 30 min. The stained cells were analyzed using a CytoFLEX Flow Cytometer.

### Drug treatment

Dox was prepared as a stock solution at 1 mg/mL in phosphate-buffered saline (PBS) at a 1:1000 dilution. CR-8 was prepared as a 1 mM stock solution in DMSO.

### Data and statistical analysis

The mRNA expression data of the clinical tissues were obtained from the Cancer Genome Atlas (TCGA) database. Overall survival was analyzed using a Kaplan–Meier plotter (https://www.kmplot.com) [25]. The MA plot was generated using RStudio. For statistical comparisons, we performed Student’s *t*-test (two-tailed) or one-way ANOVA. Pearson’s R was used to analyze the correlation data. The error bars in the figures represent the standard deviation. Statistical significance (ns, no significance; ^*^P < 0.05; ^**^P < 0.01) is indicated. Statistical analysis was performed using Prism 9.5 software (GraphPad).

## Results

### SETD1A is required for GC cell proliferation

**Fig. 1:**
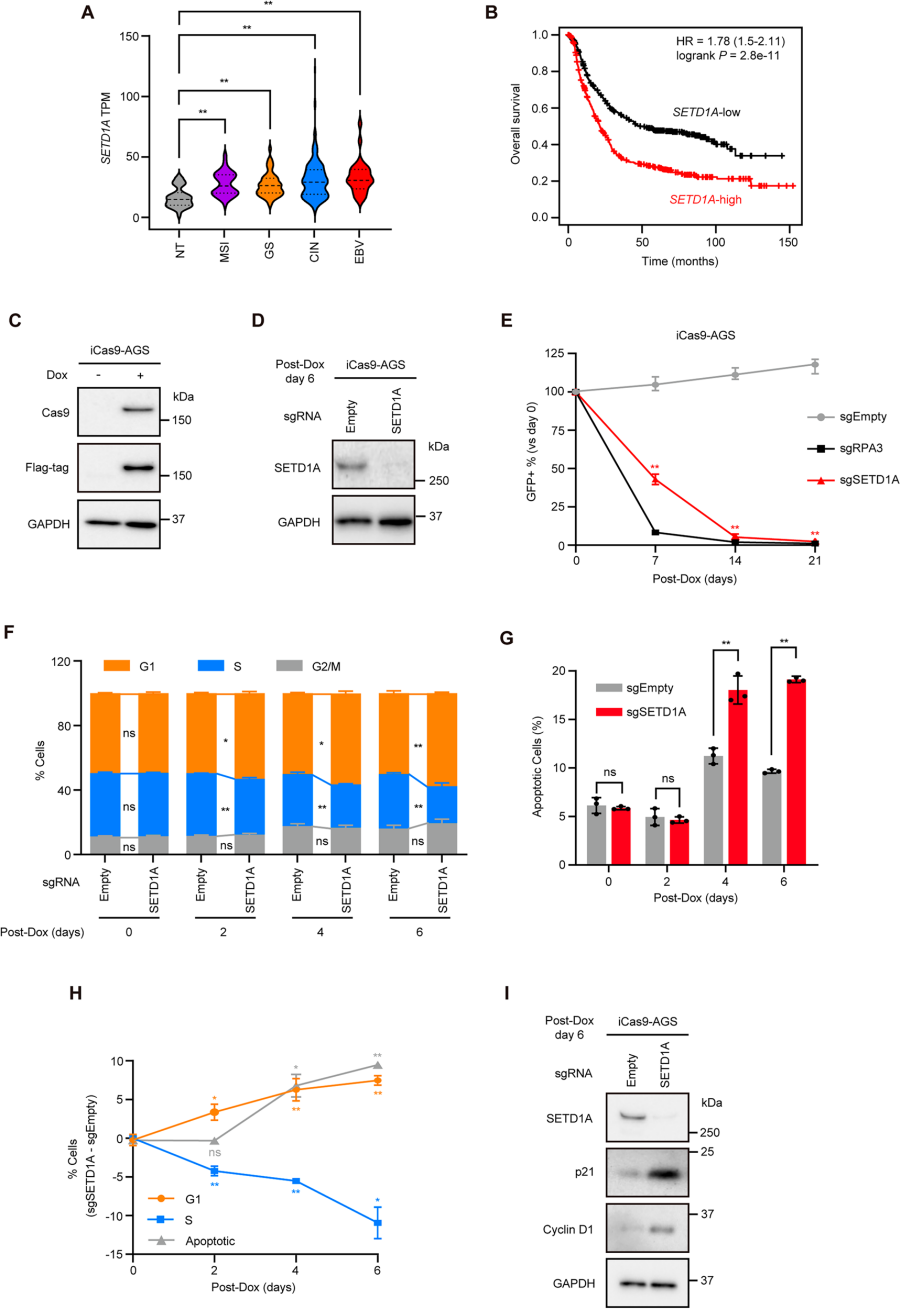
SETD1A is required for GC cell proliferation. **A** *SETD1A* mRNA expression in clinical GC subtypes from the TCGA dataset. NT, normal tissue, *n* = 36; MSI microsatellite instability, *n* = 72; GS genomically stable, *n* = 49; CIN chromosomal instability, *n* = 220; EBV Epstein–Barr virus, *n* = 30. **B** Kaplan–Meier survival analysis of patients with *SETD1A*-high vs *SETD1A*-low GC (*n* = 875). **C** Flag-tagged Cas9 expression in iCas9-AGS cells with or without 24 h of Dox treatment. **D** Western blotting quantification of SETD1A protein levels after 6 days of Dox treatment of sgRNA-expressing iCas9-AGS cells. **E** Competitive growth assay of sgRNA-expressing iCas9-AGS cells after Dox treatment. Results are presented as mean ± SD from three biological replicates. Asterisks indicate statistical significance compared with sgEmpty at each time point. **F** Cell cycle analysis via EdU incorporation at indicated time points in sgRNA-expressing iCas9-AGS cells. Results are presented as mean ± SD from three biological replicates. **G** Apoptosis was measured using Annexin V/7AAD staining at the indicated time points in sgRNA-expressing iCas9-AGS cells. Results are presented as mean ± SD from three biological replicates. **H** Alteration ratios of G1 phase, S phase, and apoptotic cells in sgSETD1A vs sgEmpty controls. Results are presented as mean ± SD from three biological replicates. Asterisks indicate statistical significance compared with day 0. **I** Western blotting results of G1 arrest markers after 6 days of Dox treatment. Representative images of three biological replicates are shown.

Using TCGA data, we found that *SETD1A* was highly expressed in four molecular subtypes of GC tissues compared to normal tissues (Fig. 1A). Elevated *SETD1A* expression correlated with poor prognosis in patients with GC (Fig. 1B). To investigate the effect of SETD1A on GC, we selected three GC cell lines (AGS, SNU719, and MKN45) from independent subtypes (GS, EBV, and CIN) in which *SETD1A* was highly expressed. iCas9-expressing single-cell clones were established from these cell lines (Figs. 1C and S1A). Using these iCas9-expressing cells, we performed a CRISPR knockout (KO) targeting *SETD1A* and observed a substantial reduction in SETD1A protein levels (Figs. 1D and S1B). *SETD1A* KO significantly suppressed the proliferation of all three cell lines (Figs. 1E and S1C). *SETD1A* KO increased the number of AGS cells undergoing G1 cell cycle arrest and apoptosis (Fig. 1F, G). G1 arrest was more rapidly induced than apoptosis (Fig. 1H). The protein levels of Cyclin D1 and p21, which are markers of G1 arrest, were markedly elevated in SETD1A-deficient AGS cells (Figs. 1I and S1D). Apoptosis and G1 arrest were also observed in the other two GC cell lines (Fig. S1E, F). As SETD1A loss induces p53-dependent apoptosis in a leukemia model [18], we investigated whether a similar mechanism occurs in GC cells. We thus generated *TP53* KO AGS cells (Fig. S1G) and subsequently depleted endogenous SETD1A. We observed that p53 loss rescued cell growth but not the apoptotic phenotype induced by *SETD1A* KO (Fig. S1H, I), suggesting that prolonged cell cycle arrest causes p53-independent apoptosis in GC cells. These results indicated that SETD1A plays an essential role in promoting GC cell proliferation and facilitating G1/S cell cycle progression.

### Non-catalytic FLOS domain of SETD1A is required for GC cell growth

**Fig. 2:**
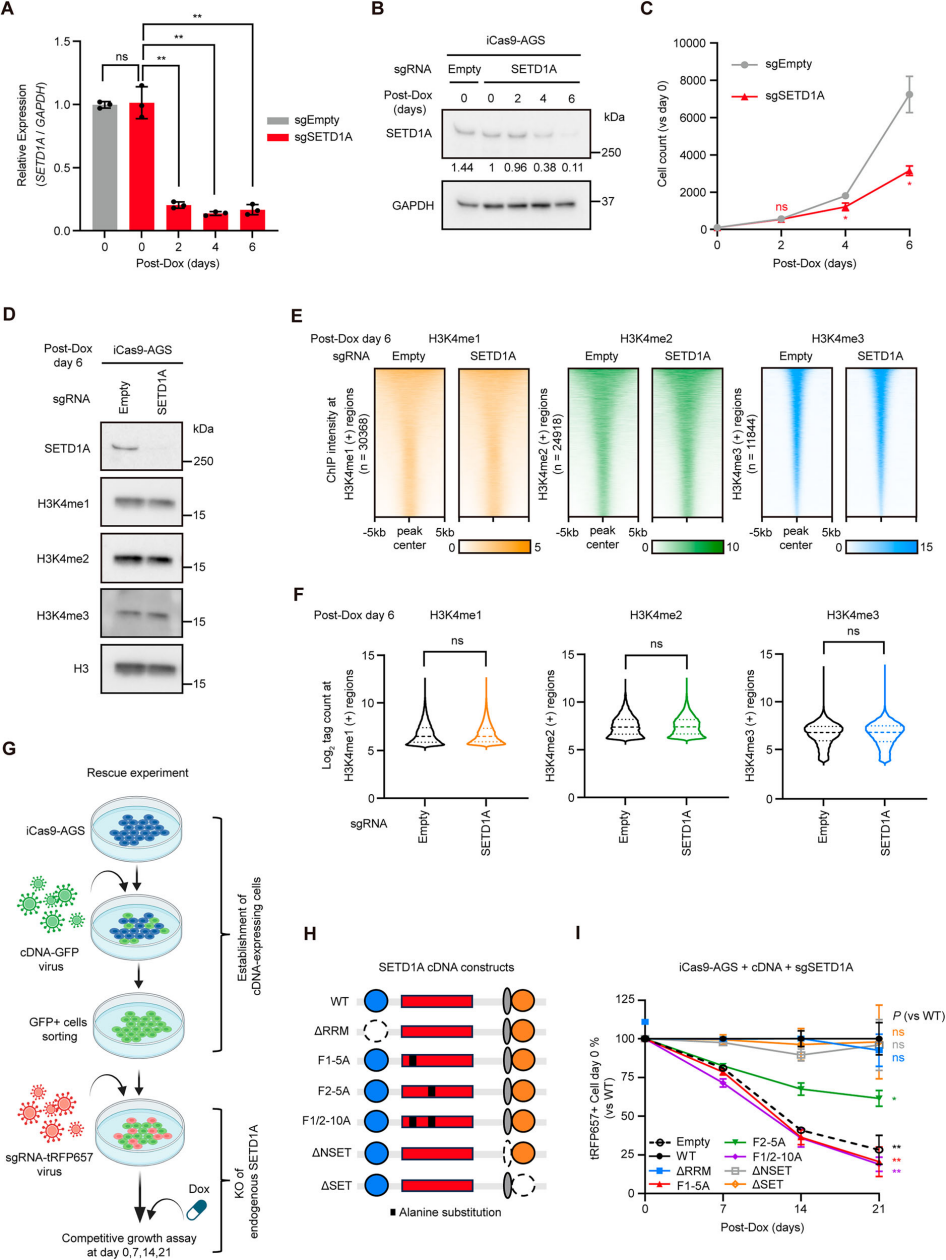
Non-catalytic FLOS domain of SETD1A is required for GC cell growth. **A** *SETD1A* mRNA levels were measured using RT-qPCR every 2 days after Dox treatment in sgRNA-expressing iCas9-AGS cells. Results are presented as mean ± SD from three biological replicates. **B** SETD1A protein levels were analyzed using western blotting at the indicated time points. **C** Cell numbers were counted every 2 days in sgRNA-expressing iCas9-AGS cells after Dox treatment. Results are presented as mean ± SD from three biological replicates. Asterisks indicate statistical significance compared with sgEmpty at each time point. **D** Western blotting results of SETD1A and H3K4me1/2/3 in sgRNA-expressing iCas9-AGS cells after 6 days of Dox treatment. Representative images of three biological replicates are shown. **E** Heatmaps of H3K4me1/2/3 ChIP-seq signals in sgRNA-expressing iCas9-AGS cells after 6 days of Dox treatment. **F** ChIP-seq signal intensities of H3K4me1/2/3 in the indicated genomic regions. **G** Workflow of rescue experiment. **H** Schematic representation of SETD1A cDNA constructs. **I** Competitive growth assay of sgSETD1A-expressing and cDNA-transduced iCas9-AGS cells after Dox treatment. Results are presented as mean ± SD from three biological replicates.

In Dox-inducible *SETD1A* KO GC cells, mRNA levels were nearly absent by day 2 in all cell lines (Fig. 2A and S2A), whereas the protein levels were markedly reduced and cell proliferation was significantly suppressed by day 6 (Figs. 2B, C and S2B, C). Therefore, we designated day 6 as the time point for *SETD1A* KO and proceeded with experiments using the AGS cell line, which is widely used in GC research. We did not observe considerable changes in H3K4 methylation using Western blotting (Figs. 2D and S3A) or ChIP-seq (Fig. 2E, F). These results suggested that a reduction in H3K4 methylation is not a critical factor in defective GC cell proliferation.

To investigate the roles of the catalytic and non-catalytic domains, we conducted a rescue experiment using six mutant constructs of SETD1A (Fig. 2G, H). Consistent with previous studies on leukemia cells, the RRM, NSET, and SET domains were nonessential, whereas the non-catalytic FLOS domain was essential for GC cell proliferation (Figs. 2I and S3B, C). These findings indicate that the FLOS domain of SETD1A, rather than the catalytic SET domain, is crucial for GC cell proliferation.

### Loss of SETD1A reduces E2F target gene expression

**Fig. 3:**
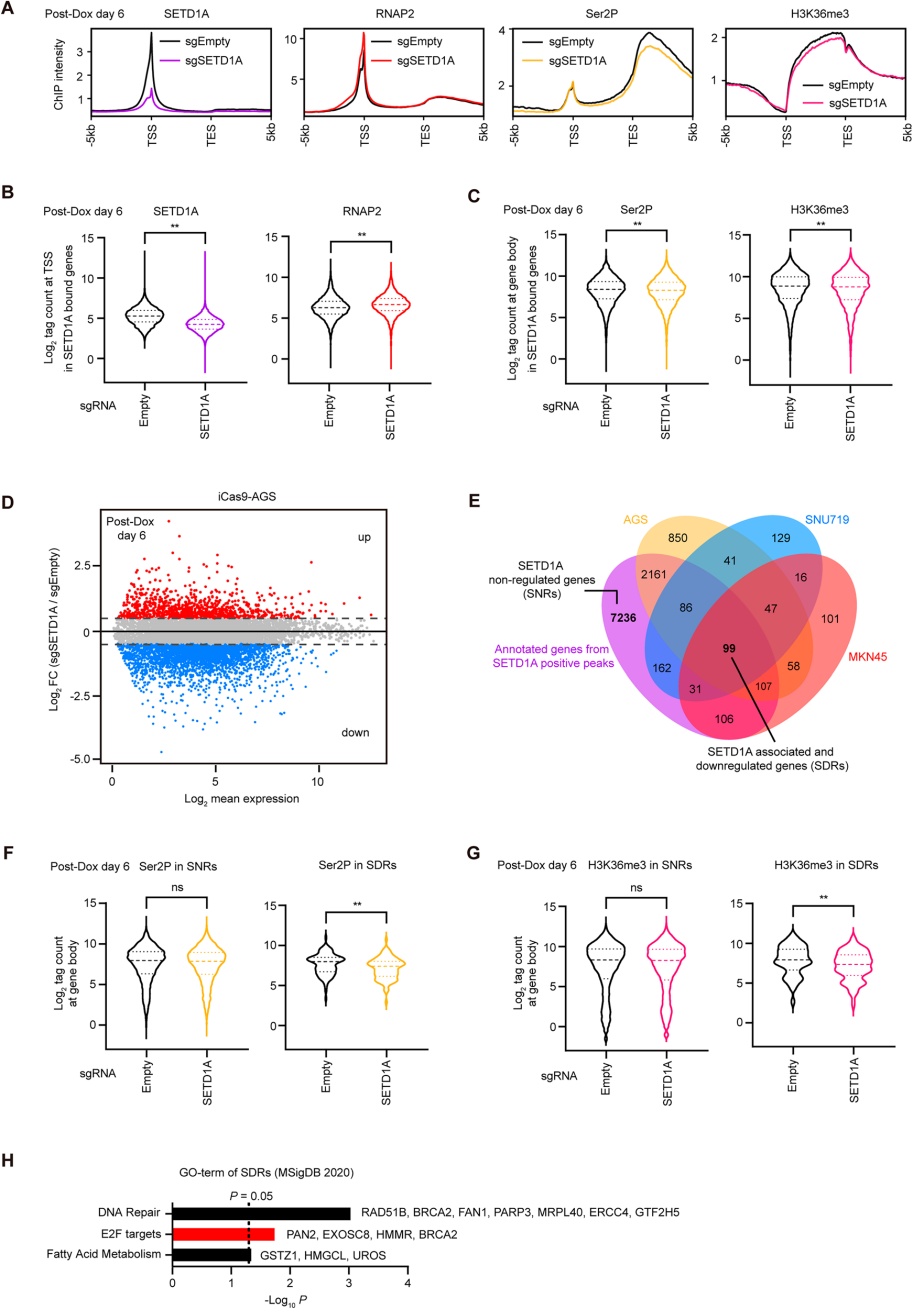
Loss of SETD1A reduces expression of E2F target genes. **A** ChIP-seq signals of SETD1A/RNAP2/Ser2P/H3K36me3 across SETD1A-bound genes (*n* = 9988) in sgRNA-expressing iCas9-AGS cells after 6 days of Dox treatment. TSS transcription start site, TES transcription end site. **B** ChIP-seq intensities of SETD1A and RNAP2 at TSS of SETD1A-bound genes in sgRNA-expressing iCas9-AGS cells. **C** ChIP-seq intensities of Ser2P and H3K36me3 in the bodies of SETD1A-bound genes in sgRNA-expressing iCas9-AGS cells. The gene body was defined as the region from 400 bp downstream of TSS to TES. **D** MA plot of RNA-seq data shows differentially expressed genes after *SETD1A* KO in AGS cells after 6 days of Dox treatment. Total, TPM > 1, *n* = 10697; upregulated, Log_2_ FC > 0.5, *n* = 1030; downregulated, Log_2_ FC < −0.5, *n* = 3449; FC fold change. **E** Overlap between SETD1A-downregulated genes in three GC cell lines and SETD1A ChIP-seq-annotated genes in AGS cells. **F**, **G** ChIP-seq intensities of Ser2P or H3K36me3 at SNRs and SDRs bodies in sgRNA-expressing iCas9-AGS cells. **H** Gene Ontology analysis of SDRs using MsigDB 2020 software. Relative genes are listed, and bars of E2F targets are indicated in red.

To further investigate the role of SETD1A in the transcriptional regulation of GC, we performed ChIP-seq of SETD1A, RNAP2, phosphorylated RNAP2 at Ser2 (Ser2P), and H3K36me3. We confirmed that defective SETD1A leads to RNAP2 accumulation at transcription start sites (TSSs) and reductions in Ser2P and H3K36me3 levels in the gene bodies of SETD1A-bound genes in AGS cells, without changing H3K4 methylation (Figs. 3A–C and S4A). To identify the gene set downregulated by *SETD1A* KO in GC cells, we performed RNA-seq assays (Figs. 3D and S4B) and extracted the downregulated (Log_2_ FC < −0.5) genes from the three SETD1A-deficient GC cell lines (Fig. 3E). To investigate the direct targets of SETD1A, we overlapped these downregulated genes with the SETD1A-associated genes that were annotated based on the SETD1A ChIP-seq data (Fig. 3E). We identified 99 genes as SETD1A-associated and downregulated genes (SDRs) and 7236 genes as SETD1A non-regulated genes (SNRs). We observed a more pronounced decline in Ser2P and H3K36me3 levels at the gene bodies of SDRs than in SNRs (Fig. 3F, G). The H3K36me3 levels began declining as early as day 2 after SETD1A depletion and progressively declined over time (Fig. S4C). This result suggests that the dysregulation of RNAP2 elongation is a direct and early consequence of SETD1A loss. In addition to the enrichment of DNA repair genes, we found that E2F target genes were significantly enriched in the SDRs (Fig. 3H). Despite the inhibition of cell cycling in *SETD1A* KO cells, we did not observe any upregulation of senescence-associated genes, such as senescence-associated secretory phenotype (SASP) or cell surface markers (Fig. S4D). These data suggest that aberrant E2F pathway activity directly causes cell cycle arrest in *SETD1A* KO GC cells.

### TAF6 is a crucial downstream target of SETD1A's non-catalytic function

**Fig. 4:**
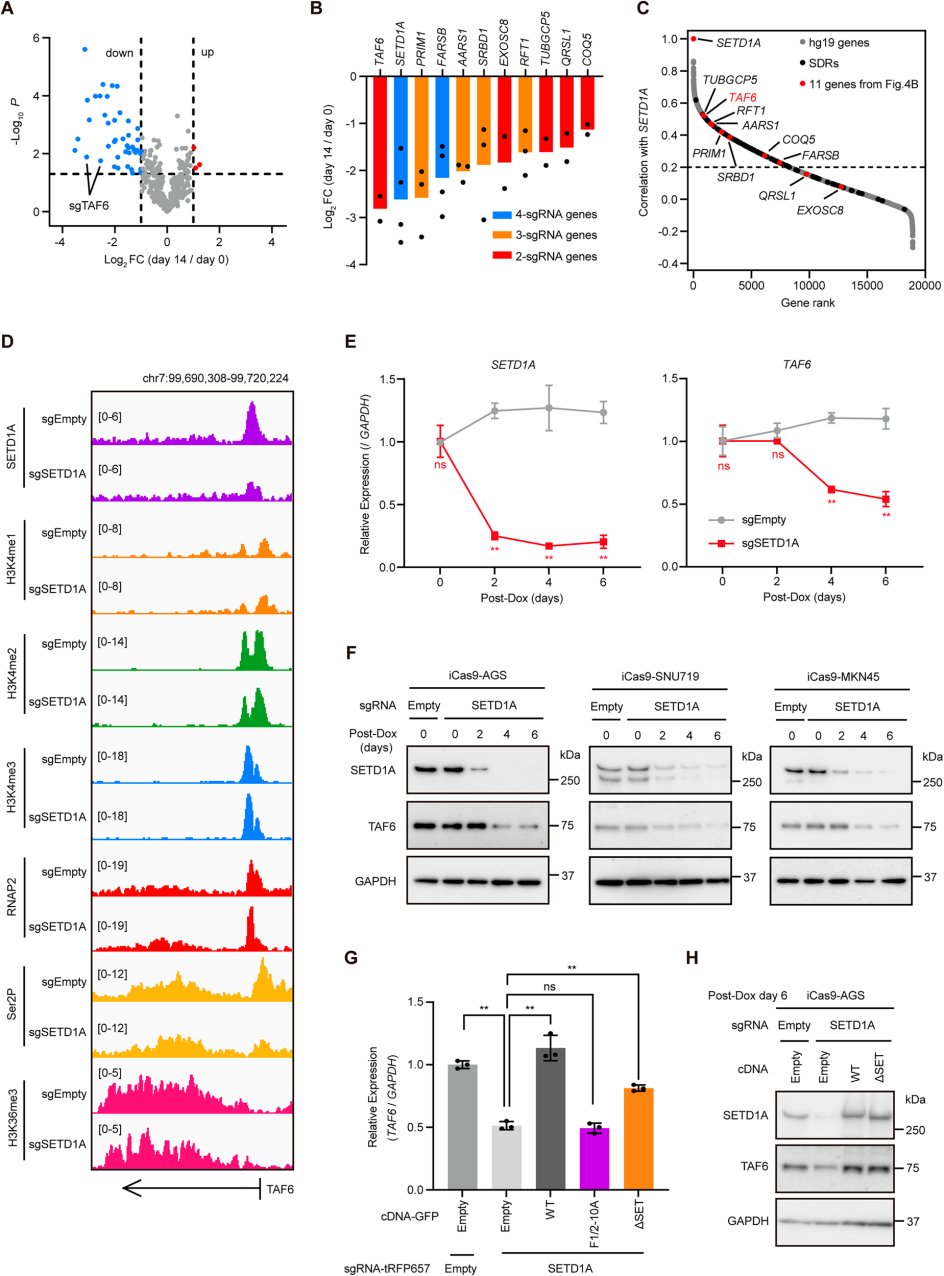
TAF6 is a crucial downstream target of SETD1A's non-catalytic function. **A** Volcano plot of CRISPR screening in pooled sgRNA-expressing iCas9-AGS cells after 14 days of Dox treatment. Total, 396 pooled sgRNAs; upregulated, Log_2_ FC > 1 and *P* < 0.05, *n* = 3; downregulated, Log_2_ FC < −1 and *P* < 0.05, *n* = 43. Three biological replicates were used. **B** sgRNA levels that decreased were aligned to each gene. Each dot represents an sgRNA. Genes containing 4, 3, and 2 efficient sgRNAs are indicated in blue, orange, and red, respectively. **C** Correlation of gene expression between SETD1A and other human genes in clinical GC samples (TCGA, *n* = 371). Total hg19 genes (gray), SDRs (black), and 11 genes selected from Fig. 4B (red) are shown. **D** IGV view of SETD1A, H3K4me1/2/3, RNAP2, Ser2P, and H3K36me3 ChIP-seq signals at TAF6 loci in sgRNA-expressing iCas9-AGS cells. **E** RT-qPCR of *SETD1A* and *TAF6* mRNA levels in sgRNA-expressing iCas9-AGS cells every 2 days after Dox treatment. Results are presented as mean ± SD from three biological replicates. Asterisks indicate statistical significance compared with sgEmpty at each time point. **F** Western blotting results of SETD1A and TAF6 protein levels in sgRNA-expressing iCas9-AGS cells every 2 days after Dox treatment. **G** RT-qPCR results for TAF6 in cDNA-transduced iCas9-AGS cells expressing sgRNAs after 6 days of Dox treatment. Results are presented as mean ± SD from three biological replicates. **H** Western blotting results of SETD1A and TAF6 in cDNA-transduced iCas9-AGS cells expressing sgRNA after 6 days of Dox treatment.

To investigate the functional targets of SETD1A in GC cells, we performed CRISPR screening, targeting the SDRs in AGS cells. We designed four sgRNAs per gene and monitored their depletion over a 14-day culture period. Using thresholds of Log_2_ FC < −1 and *P* < 0.05, we identified 43 sgRNAs whose levels were significantly reduced during cell proliferation (Figs. 4A and S5A). Based on these results, we identified 11 genes containing more than two effective sgRNAs without observing DNA repair genes, which are listed in Fig. 3H (Fig. 4B), suggesting that these DNA repair genes are not responsible for GC cell proliferation. Subsequently, *TAF6* emerged as the primary functional target of SETD1A. *TAF6* expression strongly and positively correlated with *SETD1A* expression in the clinical GC tissues (Fig. 4C). We observed the dysregulation of RNAP2/Ser2P/H3K36me3 rather than H3K4 methylation in the correlated genes, especially at the *TAF6* and *RFT1* loci (Figs. 4D and S5B, C). The mRNA and protein levels of TAF6 promptly decreased following SETD1A depletion without a reduction in the sgEmpty-expressing GC cell levels (Figs. 4E, F and S5D–F). TAF6 appeared to be regulated by the FLOS domain but not by the SET domain (Fig. 4G, H). To assess whether the Cyclin K axis regulates *TAF6* expression in GC cells, we monitored *TAF6* mRNA levels using the Cyclin K degrader CR-8 [26] or sgRNAs targeting CDK12/13 in AGS cells. The disruption of Cyclin K and CDK12/13 resulted in the downregulation of *TAF6* expression (Fig. S6A–C). These results indicate that TAF6 is regulated by the FLOS domain of SETD1A in GC cells.

### SETD1A–TAF6–p21 axis regulates GC cell G1/S phase transition

**Fig. 5:**
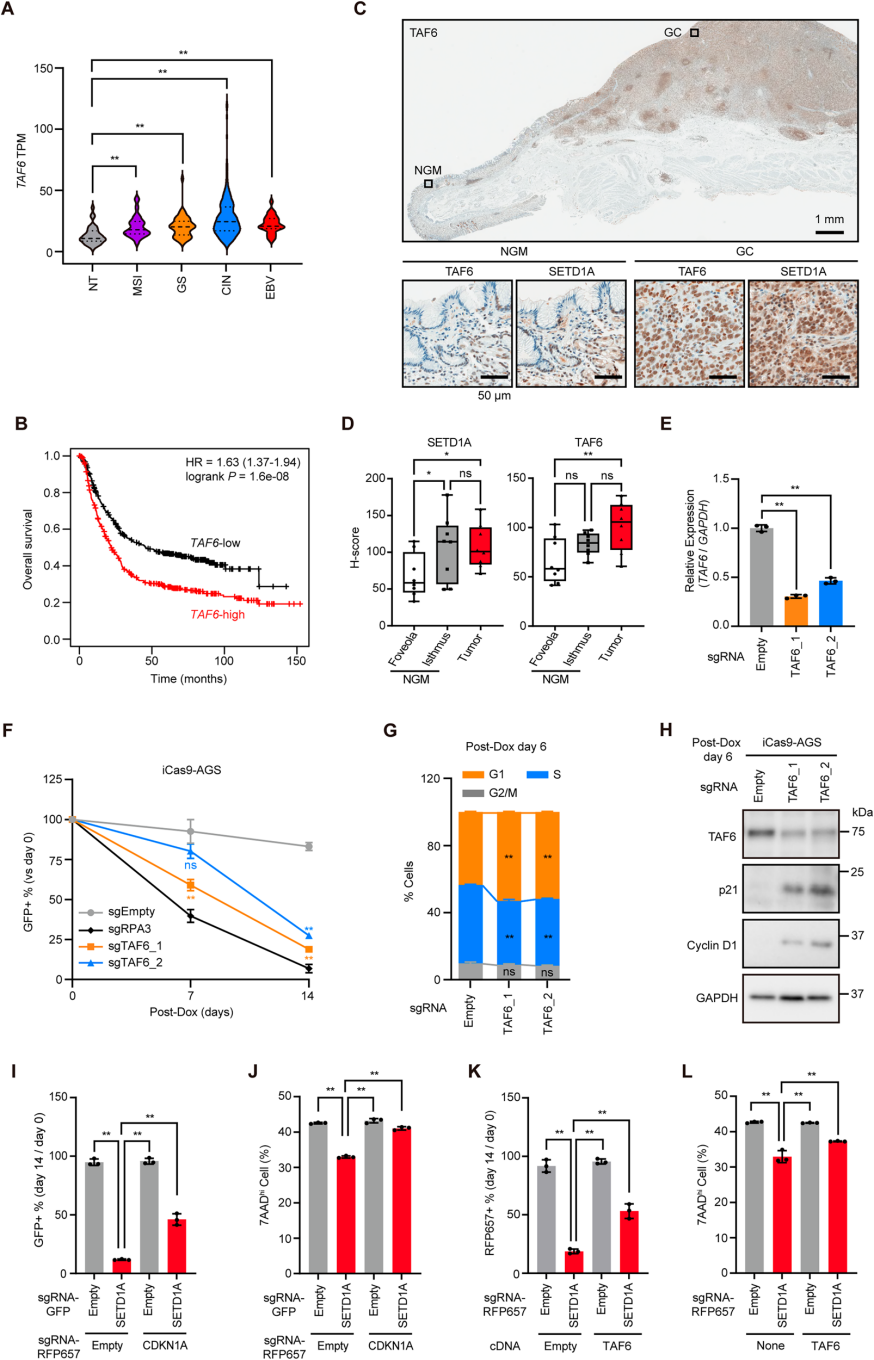
SETD1A–TAF6–p21 axis regulates G1/S phase transition in GC cells. **A** *TAF6* mRNA expression in clinical GC subtypes from the TCGA dataset. **B** Overall survival analysis of patients with *TAF6*-high vs *TAF6*-low GC (*n* = 875). **C** Representative IHC images of TAF6 and SETD1A in normal gastric mucosa (NGM) and tumor tissues. **D** *H*-scores of SETD1A and TAF6 in seven clinical samples. **E** RT-qPCR of *TAF6* mRNA in sgRNA-expressing iCas9-AGS cells after 6 days of Dox treatment. Results are presented as mean ± SD from three biological replicates. **F** Competitive growth assay results of sgRNA-expressing iCas9-AGS cells after Dox treatment every 7 days. Results are presented as mean ± SD from three biological replicates. Asterisks indicate statistical significance compared with sgEmpty at each time point. **G** Cell cycle analysis via EdU incorporation into sgRNA-expressing iCas9-AGS cells after 6 days of Dox treatment. Results are presented as mean ± SD from three biological replicates. **H** Western blotting results of G1/S-arrest markers in sgRNA-expressing iCas9-AGS cells after 6 days of Dox treatment. Representative images of three biological replicates are shown. **I** Competitive growth assay of p21-deficient AGS cells expressing Empty or SETD1A sgRNA after 14 days of Dox treatment. Results are presented as mean ± SD from three biological replicates. **J** DNA content results in p21-deficient AGS cells expressing Empty or SETD1A sgRNA after 6 days of Dox treatment. Results are presented as mean ± SD from three biological replicates. 7AAD^hi^, 7AAD high. **K** Competitive growth assay results of cDNA-transduced iCas9-AGS cells expressing sgRNA after 14 days of Dox treatment. Results are presented as mean ± SD from three biological replicates. **L** DNA content results in TAF6-overexpressing AGS cells expressing Empty or SETD1A sgRNA after 6 days of Dox treatment. Results are presented as mean ± SD from three biological replicates.

The function of TAF6 in GC had not yet been examined. We observed that *TAF6* was highly expressed in GC tissues (Fig. 5A). The overexpression of *TAF6* correlated with poor prognosis in patients with GC (Fig. 5B). The TAF6 and SETD1A proteins were expressed in the isthmus rather than in the pits of the normal gastric mucosa, and this expression was extensively observed throughout the GC tissue (Figs. 5C, D and Fig. S7A). The high SETD1A/TAF6 expression in the isthmus, a pool of stem/progenitor cells, suggested a role in GC stemness; however, the stemness marker CD44 showed comparable expression levels in SETD1A-deficient cells (Fig. S8A). To investigate the role of TAF6 in GC cell cycle progression, we used two independent sgRNAs targeting *TAF6* that efficiently reduced *TAF6* mRNA levels (Fig. 5E). *TAF6* KO significantly suppressed the proliferation of GC cells for all three subtypes (Figs. 5F and S8B). The percentage of G1-phase cells increased in TAF6-deficient AGS cells, which was followed by an increase in the levels of G1 arrest markers (Figs. 5G, H and S8C). p21 deletion significantly rescued the proliferation and G1 arrest of *SETD1A* KO AGS cells (Figs. 5I, J and S8D), suggesting that p21 is involved in SETD1A-mediated GC cell growth. Moreover, the exogenous expression of TAF6 restored the impaired cell growth and alleviated G1 cell cycle arrest caused by SETD1A deletion (Figs. 5K, L and S8E). These results suggest that the SETD1A–TAF6–p21 axis is required for cell proliferation and G1/S phase transition in GC cells.

### E2F4 cooperates with SETD1A to promote TAF6 expression

**Fig. 6:**
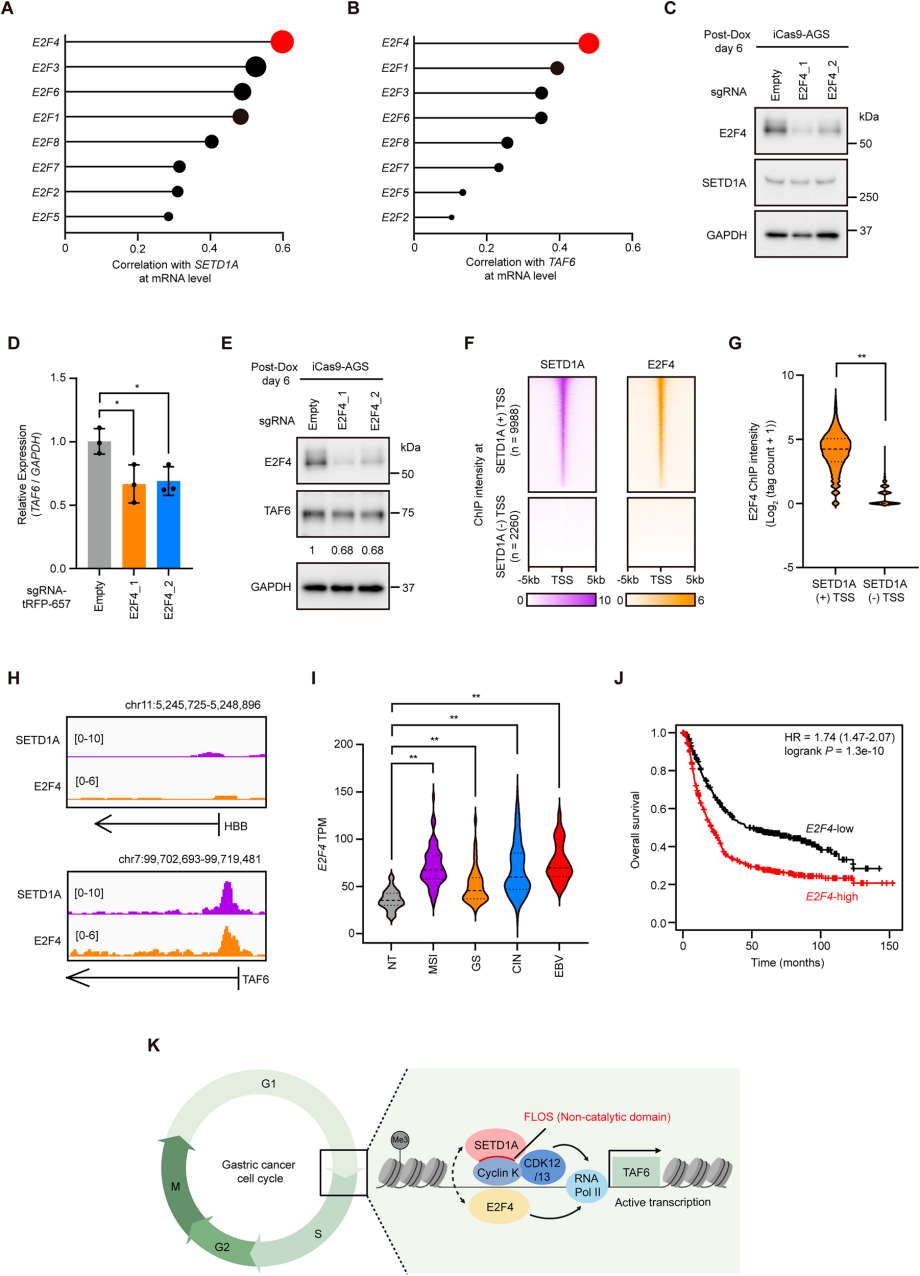
SETD1A and E2F4 cooperate to promote TAF6 expression. **A** Correlation between *SETD1A* and *E2Fs* mRNA levels in clinical GC tissue samples (TCGA, *n* = 371). *E2F4* is indicated in red. **B** Correlation between *TAF6* and *E2Fs* mRNA levels in clinical GC tissue samples (TCGA, *n* = 371). *E2F4* is indicated in red. **C** Western blotting results of E2F4 and SETD1A in sgRNA-expressing iCas9-AGS cells after 6 days of Dox treatment. **D** RT-qPCR of *TAF6* mRNA in sgRNA-expressing iCas9-AGS cells after 6 days of Dox treatment. Results are presented as mean ± SD from three biological replicates. **E** Western blotting results of E2F4 and TAF6 in sgRNA-expressing iCas9-AGS cells after 6 days of Dox treatment. **F** Heatmaps of ChIP-seq signals for SETD1A (purple) and E2F4 (orange) at SETD1A-positive and -negative TSSs in AGS cells. **G** ChIP-seq signal intensities of E2F4 at SETD1A-positive and -negative TSSs in AGS cells. **H** IGV browser views of SETD1A and E2F4 ChIP-seq signals at HBB or TAF6 loci in AGS cells. **I** *E2F4* mRNA expression in clinical GC subtypes from the TCGA dataset. **J** Overall survival analysis of patients with *E2F4*-high vs *E2F4*-low GC (*n* = 875). **K** Graphical abstract of SETD1A–TAF6 axis in GC cell cycle regulation.

G1 arrest and enrichment of E2F target genes in SETD1A-deficient GC cells suggested that SETD1A cooperates with E2Fs, which are well-known transcription factors involved in the G1/S phase transition. To evaluate the relationship between these molecules, we analyzed the mRNA levels of *SETD1A, TAF6*, and *E2Fs* in GC clinical tissues using the TCGA dataset. The expression levels of *E2F4* and *SETD1A* were positively correlated (Fig. 6A). *E2F4* expression appeared to be related to *TAF6* expression, followed by *E2F1* expression (Fig. 6B). This result suggests that E2F4 is involved in the regulation of the SETD1A–TAF6 axis. To address this possibility, we used two independent sgRNAs targeting *E2F1/4* and observed a reduction in E2F1/4 expression without the regulation of SETD1A (Figs. 6C and S9A). Interestingly, disruption of E2F4, rather than E2F1, significantly reduced *TAF6* expression, although both inhibited GC cell proliferation (Figs. 6D, E and S9B–D). These findings suggest that E2F4 serves as a SETD1A co-activator in the regulation of TAF6. We performed ChIP-seq against E2F4 in AGS cells and observed the co-localization of SETD1A and E2F4 at the SETD1A-positive TSS (Fig. 6F, G). SETD1A and E2F4 signals were enriched at the *TAF6* promoter compared to those at the negative control locus *HBB* (Fig. 6H). These results demonstrated that TAF6 is a target of SETD1A and E2F4. Consistently, we observed high expression of *E2F4* in GC clinical tissues, which was associated with poor prognosis in patients with GC (Fig. 6I, J). These results suggest that E2F4 plays a tumor-promoting role in GC and is a coregulator of the SETD1A non-catalytic function of supporting the expression of TAF6.

## Discussion

Our study revealed the function of the non-catalytic domain of SETD1A in GC. Previous studies proposed that SETD1A-dependent H3K4 methylation promotes the transcription of genes related to glycolysis and epithelial-mesenchymal transition in GC [8, 9]. These studies used two cell lines, BGC-823 and AGS; however, BGC-823 was recently reported to be a problematic cell line [27]. Therefore, it remains unclear whether SETD1A is essential for multiple GC subtypes. In this study, we confirmed the critical role of SETD1A and its non-catalytic domain and identified DNA repair and E2F pathway-associated genes as common targets of SETD1A in GC. The non-catalytic function of SETD1A is to promote the expression of genes related to DNA repair in leukemia [18]. Therefore, DNA repair-associated genes could be considered cell-context-independent targets of SETD1A. Although the E2F pathway is also regulated in leukemia cells, it has not yet been identified, possibly because of the predominant induction of apoptosis in leukemia cells [18]. Importantly, intact genome-wide H3K4 methylation was observed in GC following SETD1A deletion, as observed in ES and leukemia cells [15, 18]. Conversely, KMT2B/G primarily regulates H3K4me3 in ES cells [28]. KMT2B is also involved in regulating global H3K4me3 in leukemia [29]. Thus, KMT2B or other KMT2 family members may compensate for the loss of SETD1A regarding H3K4 methylation in GC. In contrast, the FLOS domain of SETD1A has a nonredundant function in KMT2 family members [18]. Therefore, the FLOS domain function via Cyclin K represents a unique therapeutic target for GC and other cancers. The role of the FLOS domain in immune cells and the GC microenvironment has not yet been evaluated, and we did not perform functional assays in vivo. Consequently, further in vivo studies should be conducted to examine the effectiveness of SETD1A/Cyclin K-targeting therapy in GC.

Related to cell cycle arrest prior to apoptosis in *SETD1A* KO GC cells, SETD1A depletion induces cell senescence in breast cancer cells [30]. However, gene expression profiling indicated no significant effects on cell senescence in GC. In the context of cell cycle control, we identified TAF6 as a downstream target of SETD1A. TAF6 is a subunit of TFIID that recognizes core promoters and supports the assembly of the pre-initiation complex of RNA polymerase II [31]. TAF1, the largest subunit of TFIID, is required for the transcription of Cyclin A/D1 to facilitate the G1/S progression [32]. Mouse Taf10, a homolog of human TAF10, is required for G1/S phase cell cycle progression in embryonic carcinoma cells [33]. Notably, Taf10 is indispensable for inner cell mass cell survival and fetal skin morphogenesis but is dispensable for the survival of trophoblast cells and adult keratinocytes [34, 35]. These observations suggest that TAFs play a cell context-dependent role. TAF6 upregulation by SETD1A may play a crucial role in initiating S-phase progression by activating a positive transcriptional feedback loop. While SETD1A would not be required for maintaining GC stemness, its co-expression with TAF6 in the isthmus region suggests a potential cell-context-dependent role in the adult stomach. Further studies on the role of the SETD1A–TAF6 axis will be important for understanding the role of SETD1A in both the normal stomach and GC.

We also identified E2F4 as a transcriptional activator, coupled with SETD1A, that regulates TAF6 expression. Although E2F4 is generally considered a transcriptional repressor, E2F4 promotes the proliferation and migration of GC cells by transcriptionally activating DNA replication and sister chromatid cohesion 1 (DSCC1), supporting DNA replication and repair, as well as genomic integrity during the S phase of the cell cycle [36]. E2F4 is highly expressed in ES cells and plays a transcriptional role [37]. E2F4 likely functions as a transcriptional activator in GC. Proximal labeling results showed that SETD1A and E2F4 closely localized to the chromatin in leukemia; however, their transcriptional activation functions have not been determined [22]. E2F4 has multiple functions, also forming heterodimers [38, 39]. Thus, identifying the partner proteins of the E2F4 coupled to SETD1A may provide important insights into the role of the SETD1A/E2F4–TAF6 axis in the cell cycle. Moreover, the potential roles of other E2F family members in regulating TAF6 deserve further investigation, as TAF6 expression was partially reduced upon *E2F4* KO. Further studies on the hierarchical transcriptional cascade must be conducted to comprehensively understand the SETD1A/E2F4–TAF6 axis and the effects of SETD1A inhibition in cancer. Our study findings together reveal the non-canonical roles of SETD1A in cell cycle progression in GC. These findings provide insights into the therapeutic opportunities for patients with GC or other cancers.

## Supplementary information

Supplemental Material

Original Data

## Data Availability

The accession number for the RNA-seq and ChIP-seq data reported in this study is NCBI GEO GSE273826. All other data are available in the main text or “Supplemental Material”.
