# Supplementary material for: Non-catalytic role of SETD1A promotes gastric cancer cell proliferation through the E2F4–TAF6 axis in the cell cycle
Source: Cell Death Dis. 2025 Aug 23;16(1):639. doi: 10.1038/s41419-025-07976-4 (PMC12373860; doi:10.1038/s41419-025-07976-4)
Supplement: Supplementary file 1 — Supplemental Material [file 41419_2025_7976_MOESM1_ESM.pdf]

Non-catalytic role of SETD1A promotes gastric cancer cell proliferation through the E2F4–TAF6 axis in the cell cycle

Meng Ning[1], Takayuki Hoshii[1,*], Takuya Nakagawa[2,3], Genki Usui[1], Shintaro Izumi[1], Kanako Hayashi[1], Makoto Matsumoto[1], Bahityar Rahmutulla[1], Masaki Fukuyo[1], Hiroyuki Abe[4], Tetsuo Ushiku[4], Atsushi Kaneda[1,3]

[1]Department of Molecular Oncology, Graduate School of Medicine, Chiba University, Chiba-shi, Chiba, 260-8670, Japan

[2]Department of Otorhinolaryngology, Head and Neck Surgery, Graduate School of Medicine, Chiba University, Chiba-shi, Chiba, 260-8670, Japan

[3]Health and Disease Omics Center, Chiba University, Chiba-shi, Chiba, 260-8670, Japan

[4]Department of Pathology, Graduate School of Medicine, The University of Tokyo, Tokyo, 113-0033, Japan

[*]Correspondence: hoshiit@chiba-u.jp

This file includes:

Figs. S1 to S9 and legends

Tables S1 to S3

Cell line STR profiles

Figs. S1 to S9 and legends

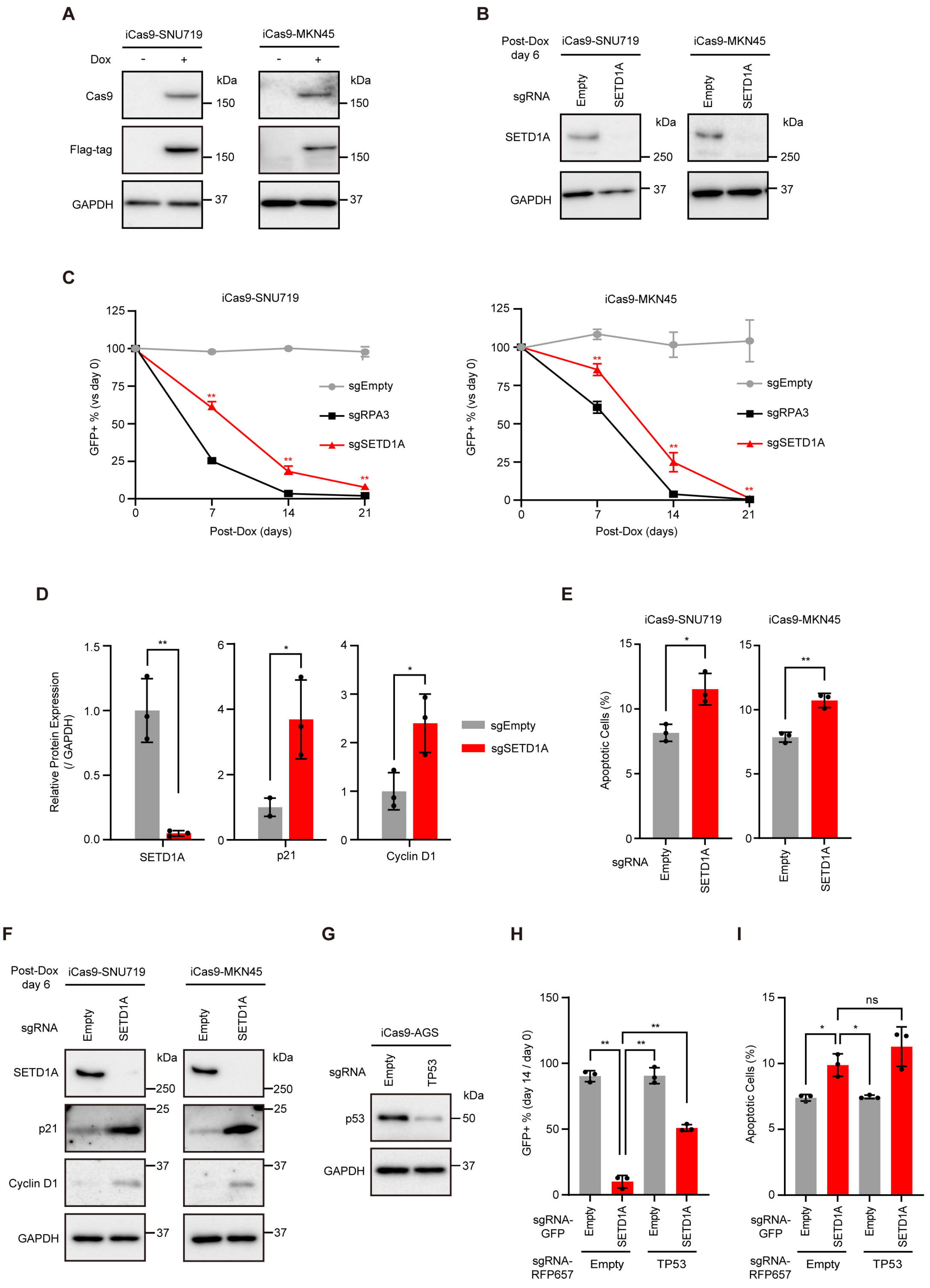

Fig. S1. SETD1A is critical for GC cell proliferation.

(A) Flag-tagged Cas9 expression in iCas9-SNU719 and iCas9-MKN45 cells with or without 24 h of Dox treatment. (B) Western blotting results of SETD1A in sgRNA-expressing iCas9-SNU719 and iCas9-MKN45 cells after 6 days of Dox treatment. (C) Competitive growth assay results of sgRNA-expressing iCas9-SNU719 and iCas9-MKN45 cells after Dox treatment. Results are presented as mean ± SD from three biological replicates. Asterisks indicate statistical significance compared with sgEmpty at each time point. (D) Quantification of Western blot band intensities normalized to those of GAPDH (Fig. 1I). Data are shown as mean ± SD from three biological replicates. (E) Apoptosis was measured using Annexin V/7AAD staining after 6 days of Dox treatment in sgRNA-expressing iCas9-SNU719 and iCas9-MKN45 cells. Results are presented as mean ± SD from three biological replicates. (F) Western blotting results of G1 arrest markers in sgRNA-expressing iCas9-SNU719 and iCas9-MKN45 cells after 6 days of Dox treatment. (G) p53 protein levels were quantified using Western blotting of sgRNA-expressing iCas9-AGS cells. (H) Competitive growth assay results of p53-deficient AGS cells expressing Empty or SETD1A sgRNA after 14 days of Dox treatment. Results are presented as mean ± SD from three biological replicates. (I) Apoptosis assay in p53-deficient AGS cells expressing Empty or SETD1A sgRNAs after 6 days of Dox treatment. Results are presented as mean ± SD from three biological replicates.

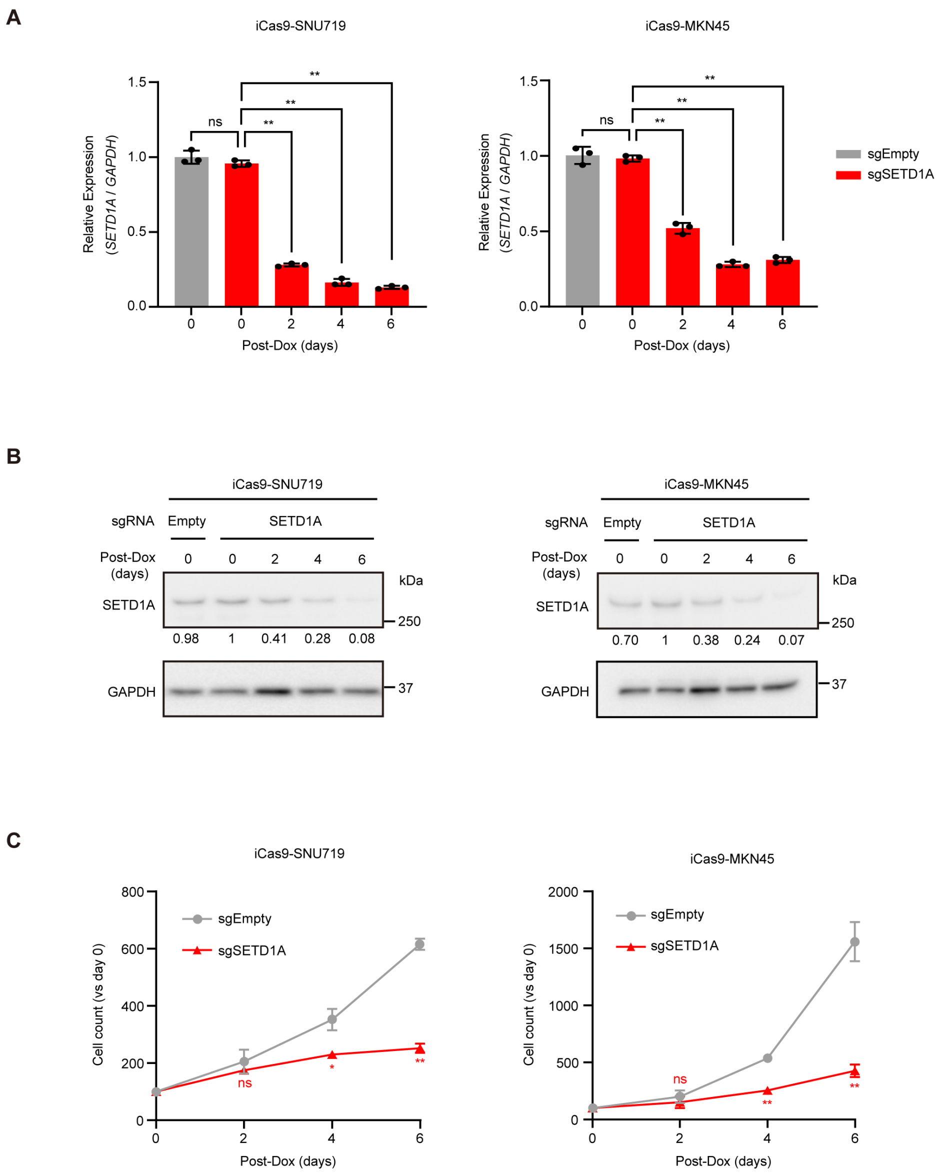

Fig. S2. Estimating *SETD1A* KO time point.

(A) RT-qPCR results of *SETD1A* mRNA levels in sgRNA-expressing iCas9-SNU719 and iCas9-MKN45 cells every 2 days after Dox treatment. Results are presented as mean ± SD from three biological replicates. (B) Western blotting

results of SETD1A protein levels in sgRNA-expressing iCas9-SNU719 and iCas9-MKN45 cells every 2 days after Dox treatment. (C) Cell numbers were counted every 2 days in sgRNA-expressing iCas9-SNU719 and iCas9-MKN45 cells after Dox treatment. Results are presented as mean ± SD from three biological replicates. Asterisks indicate statistical significance compared with sgEmpty at each time point.

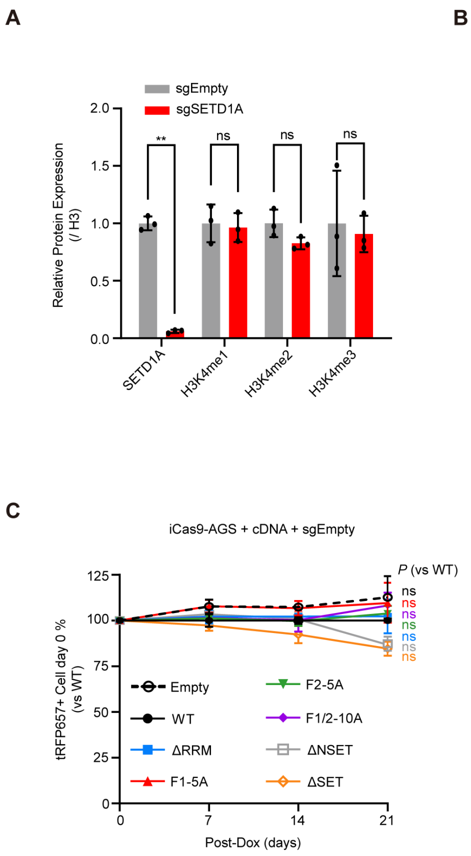

Fig. S3. Non-catalytic FLOS domain of SETD1A is essential for GC cell proliferation.

(A) Relative protein intensities were normalized to those of histone H3 (Fig. 2D). Results are presented as mean ± SD from three biological replicates. (B) Exogenous cDNA expression was detected using Western blotting. (C) Competitive growth assay results of cDNA-transduced iCas9-AGS cells expressing Empty sgRNA after Dox treatment. Results are presented as mean ± SD from three biological replicates.

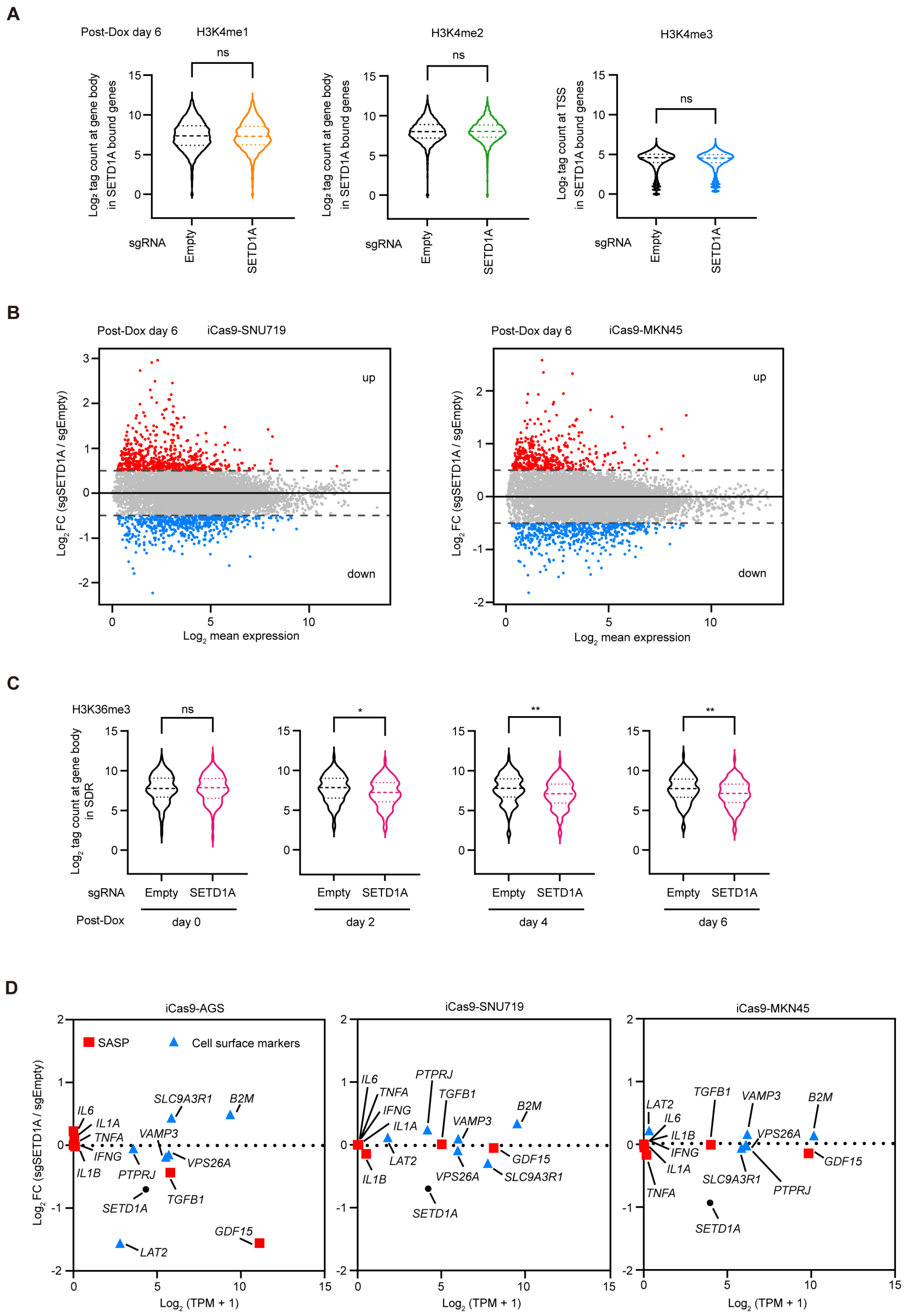
A
Post-Dox day 6
H3K4me1
H3K4me2
H3K4me3
ns
Log₂ tag count at gene body in SETD1A bound genes
Log₂ tag count at TSS in SETD1A bound genes
sgRNA
Empty
SETD1A
B
Post-Dox day 6
iCas9-SNU719
iCas9-MKN45
up
down
Log₂ FC (sgSETD1A / sgEmpty)
Log₂ mean expression
C
H3K36me3
Log₂ tag count at gene body in SDR
ns
*
**
sgRNA
Empty
SETD1A
Post-Dox
day 0
day 2
day 4
day 6
D
iCas9-AGS
iCas9-SNU719
iCas9-MKN45
SASP
Cell surface markers
Log₂ FC (sgSETD1A / sgEmpty)
Log₂ (TPM + 1)
IL6
IL1A
TNFA
IFNG
IL1B
PTPRJ
SLC9A3R1
VAMP3
VPS26A
B2M
TGFB1
SETD1A
GDF15
LAT2

Fig. S4. Loss of SETD1A induces dysregulation of RNAP2 elongation in SDRs.

(A) ChIP-seq signals of H3K4me1/2/3 in SETD1A-bound genes (n = 9988) in sgRNA-expressing iCas9-AGS cells on after 6 days of Dox treatment. (B) MA plot of RNA-seq data showing differentially expressed genes after *SETD1A* KO in SNU719 and MKN45 cells on after 6 days of Dox treatment. (C) ChIP-seq intensities of H3K36me3 in bodies of SDRs. (D) mRNA levels of senescence-associated secretory phenotype (SASP) and cell-surface-marker-related genes in *SETD1A* KO GC cells. Log_2 FC (sgSETD1A vs. sgEmpty) and Log_2 (TPM + 1) in sgEmpty-expressing iCas9-cells are described for three cell lines. *SETD1A*, SASP, and cell-surface-marker-related genes are marked in black, red, and blue, respectively.

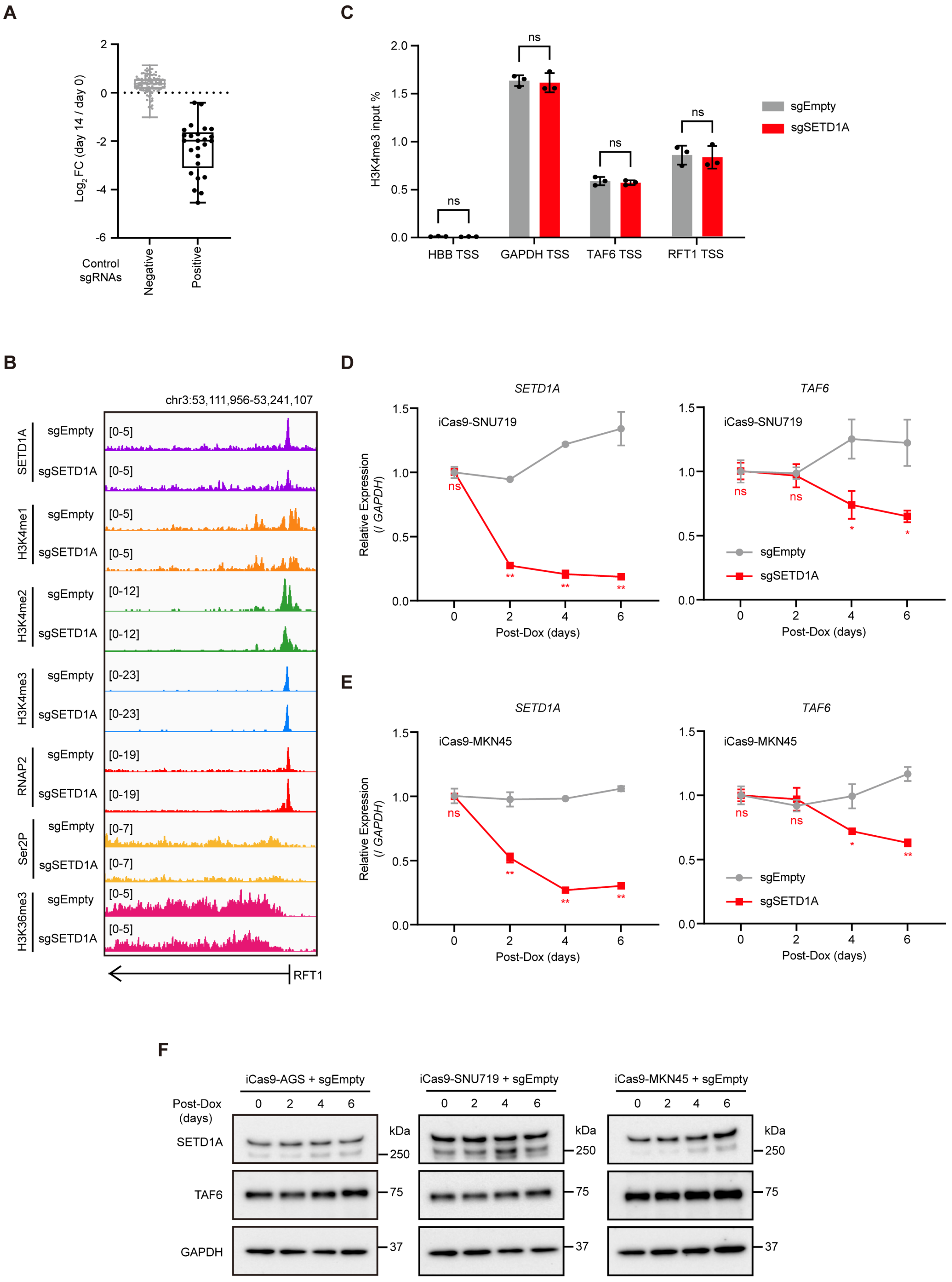

A
Log2FC (day 14 / day 0)
Control sgRNAs
Negative
Positive
B
chr3:53,111,956-53,241,107
SETD1A
H3K4me1
H3K4me2
H3K4me3
RNAP2
Ser2P
H3K36me3
sgEmpty
sgSETD1A
[0-5]
[0-12]
[0-23]
[0-19]
[0-7]
RFT1
C
H3K4me3 input %
ns
HBB TSS
GAPDH TSS
TAF6 TSS
RFT1 TSS
sgEmpty
sgSETD1A
D
SETD1A
TAF6
iCas9-SNU719
Relative Expression (/ GAPDH)
Post-Dox (days)
E
iCas9-MKN45
F
iCas9-AGS + sgEmpty
iCas9-SNU719 + sgEmpty
iCas9-MKN45 + sgEmpty
Post-Dox (days)
SETD1A
TAF6
GAPDH
kDa
250
75
37

Fig. S5. TAF6 is a crucial downstream target of SETD1A.

(A) Log_2 FC of negative (n = 110) and positive (n = 25) control sgRNAs in CRISPR screen. Three biological replicates were used. (B) IGV browser view of SETD1A, H3K4me1/2/3, RNAP2, Ser2P, and H3K36me3 ChIP-seq signals at RFT1 loci in sgRNA-expressing iCas9-AGS cells. (C) H3K4me3 ChIP-qPCR at indicated target loci using specific primers in sgRNA-expressing iCas9-AGS cells after 6 days of Dox treatment. (D,E) RT-qPCR of *SETD1A* and *TAF6* mRNA levels in sgRNA-expressing iCas9-SNU719 and iCas9-MKN45 cells every 2 days after Dox treatment. Results are presented as mean ± SD from three biological replicates. Asterisks indicate statistical significance compared with sgEmpty at each time point. (F) Western blotting results of SETD1A and TAF6 protein levels in sgEmpty-expressing GC cells every 2 days after Dox treatment.

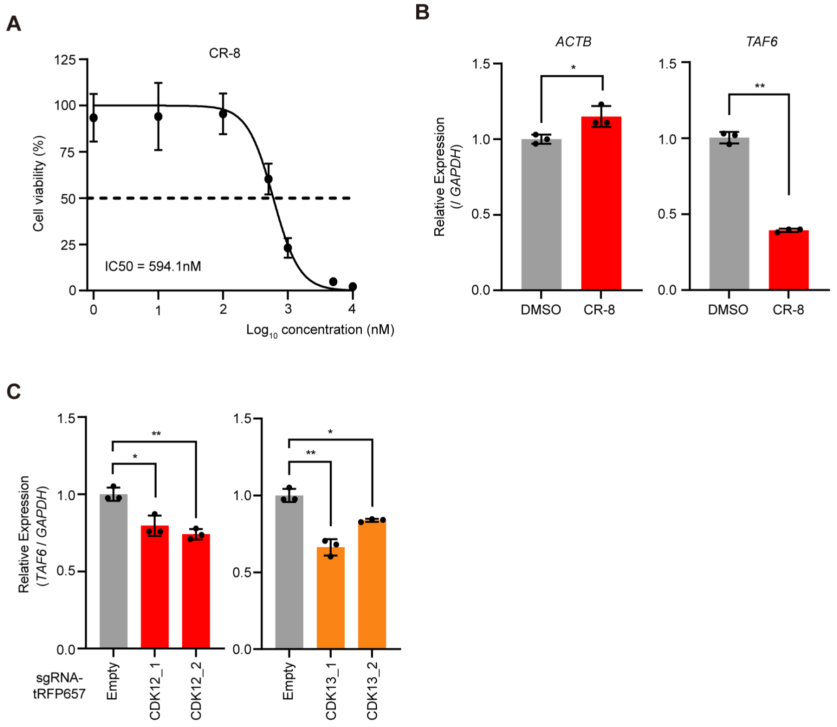

Fig. S6. *TAF6* expression is regulated by cofactors of SETD1A FLOS domain.

(A) AGS cells were treated with various doses of CR-8 for 72 h. Dotted black line indicates Half-maximal inhibitory concentration (IC50). Results are presented as mean ± SD from three biological replicates. (B) RT-qPCR results of *ACTB* and *TAF6* mRNA levels in AGS cells treated with 0.1% DMSO or 1 μM CR-8 for 24 h. Results are presented as mean ± SD from three biological replicates. (C) RT-qPCR results of *TAF6* mRNA levels in sgRNA-expressing iCas9-AGS cells after 6 days of Dox treatment. Results are presented as mean ± SD from three biological replicates.

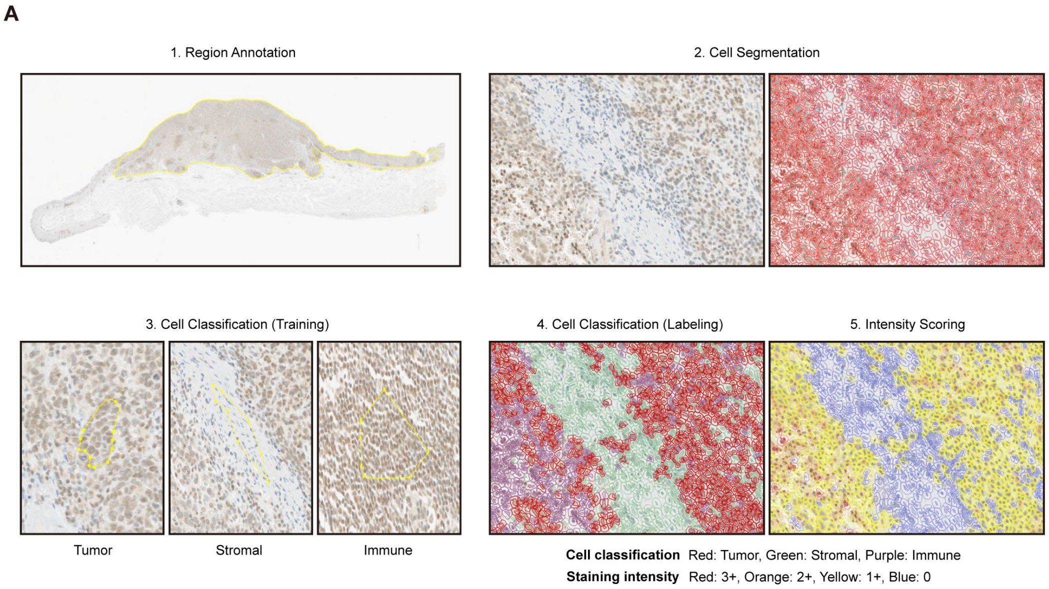
A
1. Region Annotation
2. Cell Segmentation
3. Cell Classification (Training)
4. Cell Classification (Labeling)
5. Intensity Scoring
Tumor
Stromal
Immune
Cell classification Red: Tumor, Green: Stromal, Purple: Immune
Staining intensity Red: 3+, Orange: 2+, Yellow: 1+, Blue: 0

Fig. S7. Workflow for cell type – specific immunostaining analysis using QuPath.

(A) This figure illustrates the stepwise procedure for quantitative image analysis of immunohistochemically stained gastric cancer tissue using QuPath software. 1. Tumor Region Annotation: The tumor region (outlined in yellow) was manually annotated based on histological morphology by a board-certified pathologist. 2. Cell Segmentation: The left panel shows the raw image, while the right panel displays the result after cell detection. Segmentation outlines shown in red indicate detected nuclei and cytoplasm. 3. Cell Classification (Training): Representative cells (outlined in yellow) were manually annotated by a pathologist to serve as training data for an object classifier using the Random Trees (RTrees) algorithm. Cells were annotated as tumor (left), stromal (middle), or immune (right) cells. 4. Cell Classification (Labeling): The trained classifier was applied to label all segmented cells (Red: Tumor cells, Green: Stromal cells, Purple: Immune cells). 5. Intensity Scoring: The “Positive Cell Detection” function was used to assign nuclear DAB intensity scores (0–3) based on optical density measurements.

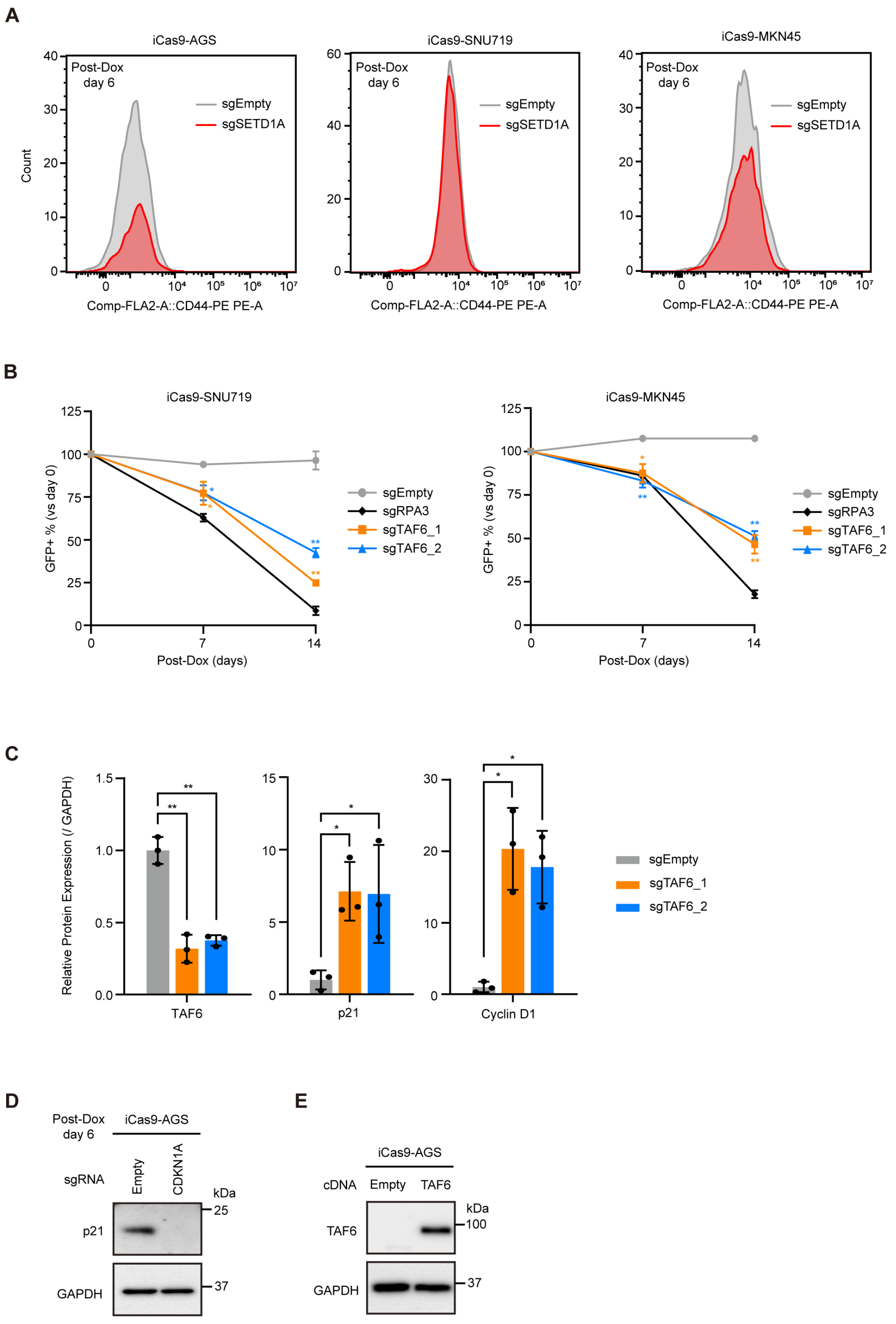
A
iCas9-AGS
iCas9-SNU719
iCas9-MKN45
Post-Dox day 6
sgEmpty
sgSETD1A
Count
Comp-FLA2-A::CD44-PE PE-A
B
iCas9-SNU719
iCas9-MKN45
GFP+ % (vs day 0)
Post-Dox (days)
sgEmpty
sgRPA3
sgTAF6_1
sgTAF6_2
C
Relative Protein Expression (/ GAPDH)
TAF6
p21
Cyclin D1
sgEmpty
sgTAF6_1
sgTAF6_2
D
Post-Dox day 6
iCas9-AGS
sgRNA
Empty
CDKN1A
kDa
p21
GAPDH
25
37
E
iCas9-AGS
cDNA
Empty
TAF6
kDa
TAF6
GAPDH
100
37

Fig. S8. TAF6 is essential for GC cell proliferation.

(A) CD44 staining of sgRNA-expressing GC cell lines treated with Dox for 6 days. (B) Competitive growth assay of sgRNA-expressing iCas9-SNU719 and iCas9-MKN45 cells after Dox treatment. Results are presented as mean ± SD from three biological replicates. Asterisks indicate statistical significance compared with sgEmpty at each time point. (C) Relative protein intensities were normalized to those of GAPDH (Fig. 5H). Results are presented as mean ± SD from three biological replicates. (D) Western blotting results of exogenous TAF6 in cDNA-transduced iCas9-AGS cells after 7 days of blasticidin selection. (E) p21 protein levels were quantified using Western blotting in sgRNA-expressing iCas9-AGS cells.

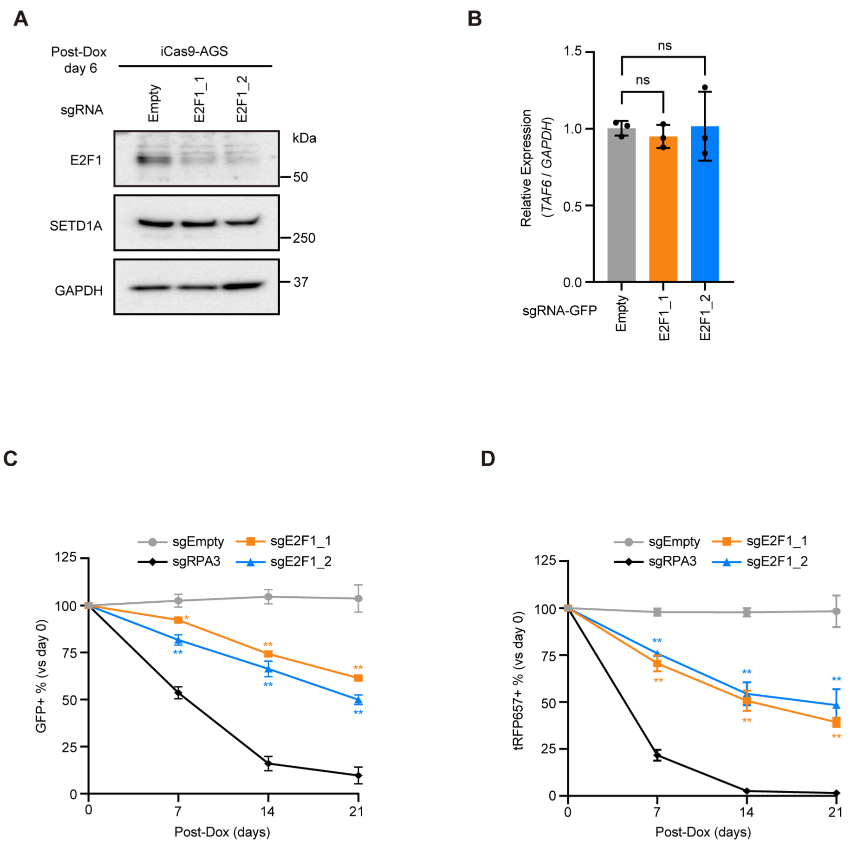

Fig. S9. SETD1A and E2F4 cooperate to promote TAF6 expression.

(A) Western blotting results of E2F1 and SETD1A protein levels in sgRNA-expressing GC cells after 6 days of Dox treatment. (B) RT-qPCR results of *TAF6* mRNA levels in sgRNA-expressing iCas9-AGS cells after 6 days of Dox treatment. (C,D) Competitive growth assay results of sgRNA-expressing iCas9-AGS cells after Dox treatment. Results are presented as mean ± SD from three biological replicates. Asterisks indicate statistical significance compared with sgEmpty at each time point.

Table S1. sgRNAs.

Name	sgRNA sequence	Source and Notes
sgSETD1A	AAGAGCATGGAGAAGCCTGC	Hoshii T et al. *Cell Reports*, 2022
sgRPA3	GATGAATTGAGCTAGCATGC	Hoshii T et al. *Cell Reports*, 2022
sgCDK12_1	AACACTTAATATCCCGATGC	Hoshii T et al. *Cell Reports*, 2022
sgCDK12_2	CGACCTCCAGAACTACTGCT	Hoshii T et al. *Cell Reports*, 2022
sgCDK13_1	ATTTATGAGACAGCTCATGG	Hoshii T et al. *Cell Reports*, 2022
sgCDK13_2	TTTTAGTGAAGAGTTCGCCA	Hoshii T et al. *Cell Reports*, 2022
sgTAF6_1	GAGATAAAGGTACTGAACCG	
sgTAF6_2	GAGCCATAACGAGTCGTCCA	
sgE2F1_1	TCATAGCGTGACTTCTCCCC	
sgE2F1_2	AGTCCAAGAACCACATCCAG	
sgE2F4_1	CCCTAGCTGTACGCCAGAAG	Hoshii T et al. *Nucleic Acids Res*, 2024
sgE2F4_2	GATGTCCTCATGAGTGACGT	Hoshii T et al. *Nucleic Acids Res*, 2024
sgCDKN1A	GTCACCGAGACACCACTGGA	
sgTP53	GCATGGGCGGCATGAACCGG	
HGLibA_64384	ACGGAGGCTAAGCGTCGCAA	Non-targeting control
HGLibA_64385	CGCTTCCGCGGCCCGTTCAA	Non-targeting control
HGLibA_64386	ATCGTTTCCGCTTAACGGCG	Non-targeting control
HGLibA_64387	GTAGGCGCGCCGCTCTCTAC	Non-targeting control
HGLibA_64388	CCATATCGGGGCGAGACATG	Non-targeting control
HGLibA_64389	TACTAACGCCGCTCCTACAG	Non-targeting control
HGLibA_64390	TGAGGATCATGTCGAGCGCC	Non-targeting control
HGLibA_64391	GGGCCCGCATAGGATATCGC	Non-targeting control
HGLibA_64392	TAGACAACCGCGGAGAATGC	Non-targeting control
HGLibA_64393	ACGGGCGGCTATCGCTGACT	Non-targeting control
HGLibA_64394	CGCGGAAATTTTACCGACGA	Non-targeting control
HGLibA_64395	CTTACAATCGTCGGTCCAAT	Non-targeting control
HGLibA_64396	GCGTGCGTCCCGGGTTACCC	Non-targeting control
HGLibA_64397	CGGAGTAACAAGCGGACGGA	Non-targeting control
HGLibA_64398	CGAGTGTTATACGCACCGTT	Non-targeting control
HGLibA_64399	CGACTAACCGGAAACTTTTT	Non-targeting control
HGLibA_64400	CAACGGGTTCTCCCGGCTAC	Non-targeting control
HGLibA_64401	CAGGAGTCGCCGATACGCGT	Non-targeting control
HGLibA_64402	TTCACGTCGTCTCGCGACCA	Non-targeting control
HGLibA_64403	GTGTCGGATTCCGCCGCTTA	Non-targeting control

HGLibA_64404	CACGAACTCACACCGCGCGA	Non-targeting control
HGLibA_64405	CGCTAGTACGCTCCTCTATA	Non-targeting control
HGLibA_64406	TCGCGCTTGGGTTATACGCT	Non-targeting control
HGLibA_64407	CTATCTCGAGTGGTAATGCG	Non-targeting control
HGLibA_64408	AATCGACTCGAACTTCGTGT	Non-targeting control
HGLibA_64409	CCCGATGGACTATACCGAAC	Non-targeting control
HGLibA_64410	ACGTTCGAGTACGACCAGCT	Non-targeting control
HGLibA_64411	CGCGACGACTCAACCTAGTC	Non-targeting control
HGLibA_64412	GGTCACCGATCGAGAGCTAG	Non-targeting control
HGLibA_64413	CTCAACCGACCGTATGGTCA	Non-targeting control
HGLibA_64414	CGTATTCGACTCTCAACGCG	Non-targeting control
HGLibA_64415	CTAGCCGCCCAGATCGAGCC	Non-targeting control
HGLibA_64416	GAATCGACCGACACTAATGT	Non-targeting control
HGLibA_64417	ACTTCAGTTCGGCGTAGTCA	Non-targeting control
HGLibA_64418	GTGCGATGTCGCTTCAACGT	Non-targeting control
HGLibA_64419	CGCCTAATTTCCGGATCAAT	Non-targeting control
HGLibA_64420	CGTGGCCGGAACCGTCATAG	Non-targeting control
HGLibA_64421	ACCCTCCGAATCGTAACGGA	Non-targeting control
HGLibA_64422	AAACGGTACGACAGCGTGTG	Non-targeting control
HGLibA_64423	ACATAGTCGACGGCTCGATT	Non-targeting control
HGLibA_64424	GATGGCGCTTCAGTCGTCGG	Non-targeting control
HGLibA_64425	ATAATCCGGAAACGCTCGAC	Non-targeting control
HGLibA_64426	CGCCGGGCTGACAATTAACG	Non-targeting control
HGLibA_64427	CGTCGCCATATGCCGGTGGC	Non-targeting control
HGLibA_64428	CGGGCCTATAACACCATCGA	Non-targeting control
HGLibA_64429	CGCCGTTCCGAGATACTTGA	Non-targeting control
HGLibA_64430	CGGGACGTCGCGAAAATGTA	Non-targeting control
HGLibA_64431	TCGGCATACGGGACACACGC	Non-targeting control
HGLibA_64432	AGCTCCATCGCCGCGATAAT	Non-targeting control
HGLibA_64433	ATCGTATCATCAGCTAGCGC	Non-targeting control
HGLibA_64434	TCGATCGAGGTTGCATTCGG	Non-targeting control
HGLibA_64435	CTCGACAGTTCGTCCCGAGC	Non-targeting control
HGLibA_64436	CGGTAGTATTAATCGCTGAC	Non-targeting control
HGLibA_64437	TGAACGCGTGTTTCCTTGCA	Non-targeting control
HGLibA_64438	CGACGCTAGGTAACGTAGAG	Non-targeting control
HGLibA_64439	CATTGTTGAGCGGGCGCGCT	Non-targeting control
HGLibA_64440	CCGCTATTGAAACCGCCCAC	Non-targeting control
HGLibA_64441	AGACACGTCACCGGTCAAAA	Non-targeting control
HGLibA_64442	TTTACGATCTAGCGGCGTAG	Non-targeting control
HGLibA_64443	TTCGCACGATTGCACCTTGG	Non-targeting control

HGLibA_64444	GGTTAGAGACTAGGCGCGCG	Non-targeting control
HGLibA_64445	CCTCCGTGCTAACGCGGACG	Non-targeting control
HGLibA_64446	TTATCGCGTAGTGCTGACGT	Non-targeting control
HGLibA_64447	TACGCTTGCGTTTAGCGTCC	Non-targeting control
HGLibA_64448	CGCGGCCCACGCGTCATCGC	Non-targeting control
HGLibA_64449	AGCTCGCCATGTCGGTTCTC	Non-targeting control
HGLibA_64450	AACTAGCCCGAGCAGCTTCG	Non-targeting control
HGLibA_64451	CGCAAGGTGTCGGTAACCCT	Non-targeting control
HGLibA_64452	CTTCGACGCCATCGTGCTCA	Non-targeting control
HGLibA_64453	TCCTGGATACCGCGTGGTTA	Non-targeting control
HGLibA_64454	ATAGCCGCCGCTCATTACTT	Non-targeting control
HGLibA_64455	GTCGTCCGGGATTACAAAAT	Non-targeting control
HGLibA_64456	TAATGCTGCACACGCCGAAT	Non-targeting control
HGLibA_64457	TATCGCTTCCGATTAGTCCG	Non-targeting control
HGLibA_64458	GTACCATACCGCGTACCCTT	Non-targeting control
HGLibA_64459	TAAGATCCGCGGGTGGCAAC	Non-targeting control
HGLibA_64460	GTAGACGTCGTGAGCTTCAC	Non-targeting control
HGLibA_64461	TCGCGGACATAGGGCTCTAA	Non-targeting control
HGLibA_64462	AGCGCAGATAGCGCGTATCA	Non-targeting control
HGLibA_64463	GTTCGCTTCGTAACGAGGAA	Non-targeting control
HGLibA_64464	GACCCCCGATAACTTTTGAC	Non-targeting control
HGLibA_64465	ACGTCCATACTGTCGGCTAC	Non-targeting control
HGLibA_64466	GTACCATTGCCGGCTCCCTA	Non-targeting control
HGLibA_64467	TGGTTCCGTAGGTCGGTATA	Non-targeting control
HGLibA_64468	TCTGGCTTGACACGACCGTT	Non-targeting control
HGLibA_64469	CGCTAGGTCCGGTAAGTGCG	Non-targeting control
HGLibA_64470	AGCACGTAATGTCCGTGGAT	Non-targeting control
HGLibA_64471	AAGGCGCGCGAATGTGGCAG	Non-targeting control
HGLibA_64472	ACTGCGGAGCGCCCAATATC	Non-targeting control
HGLibA_64473	CGTCGAGTGCTCGAACTCCA	Non-targeting control
HGLibA_64474	TCGCAGCGGCGTGGGATCGG	Non-targeting control
HGLibA_64475	ATCTGTCCTAATTCGGATCG	Non-targeting control
HGLibA_64476	TGCGGCGTAATGCTTGAAAG	Non-targeting control
HGLibA_64477	CGAACTTAATCCCGTGGCAA	Non-targeting control
HGLibA_64478	GCCGTGTTGCTGGATACGCC	Non-targeting control
HGLibA_64479	TACCCTCCGGATACGGACTG	Non-targeting control
HGLibA_64480	CCGTTGGACTATGGCGGGTC	Non-targeting control
HGLibA_64481	GTACGGGGCGATCATCCACA	Non-targeting control
HGLibA_64482	AAGAGTAGTAGACGCCCGGG	Non-targeting control
HGLibA_64483	AAGAGCGAATCGATTTCGTG	Non-targeting control

AAVS1_1	GTCCCCTCCACCCCACAGTG	Negative control
AAVS1_2	GGGGCCACTAGGGACAGGAT	Negative control
AAVS1_3	AGAGCCACATTAACCGGCCC	Negative control
AAVS1_4	GGGACCACCTTATATTCCCA	Negative control
AAVS1_5	TAAGGAATCTGCCTAACAGG	Negative control
CCR5_255	GCAGCATAGTGAGCCCAGAA	Negative control
CCR5_312	CAATGTGTCAACTCTTGACA	Negative control
CCR5_372	TCATCCTCCTGACAATCGAT	Negative control
CCR5_454	GGTGACAAGTGTGATCACTT	Negative control
CCR5_600	CATTAAAGATAGTCATCTTG	Negative control
RPA3_21	TGGACATGATGGACTTGCCC	Positive control
RPA3_46	GATGAATTGAGCTAGCATGC	Positive control
RPA3_160	TACGGGTTCCATCAACTCGA	Positive control
RPA3_196	TGAAGAAATCTCTGGAATTG	Positive control
RPA3_223	GGTTGGAAGAGTAACCGCCA	Positive control
PCNA_82	GCTGGAGCTAATATCCCAGC	Positive control
PCNA_157	GTCGAAGCCCTCAGACCGCA	Positive control
PCNA_283	ACTAAGGGCCGAAGATAACG	Positive control
PCNA_374	GATTTAGATGTTGAACAACT	Positive control
PCNA_423	ATACGTGCAAATTCACCAGA	Positive control
POLR2A_1220	CTTCAAGAACTAGTGCGCAG	Positive control
POLR2A_1569	TGGGGGGTGACAATCATGCG	Positive control
POLR2A_2377	CGTTGGACAGCAGAACGTCG	Positive control
POLR2A_3145	CATGCGGCGGGAACACAACG	Positive control
POLR2A_3482	TCAACGTTGTATGCTCCAGA	Positive control
POLR2L_15	CCACAAGTGAAGCAGCGTAC	Positive control
POLR2L_26	CCTGTACGCTGCTTCACTTG	Positive control
POLR2L_38	TTCACTTGTGGCAAGATCGT	Positive control
POLR2L_49	CAAGATCGTCGGCAACAAGT	Positive control
POLR2L_90	TGCAGGCCGAGTACACCGAG	Positive control
SF3B3_375	TCTTAGCTGTGGATCCCAAA	Positive control
SF3B3_589	AGCAGACAATGATCCAACAG	Positive control
SF3B3_1266	CCACAGGCCACATACAACTG	Positive control
SF3B3_1935	TGTGTATCGTGGAAATGGGT	Positive control
SF3B3_2156	AAGAATAGCTCAACCATGAG	Positive control
AARS_1487	TTGGACTCCAGTGGTAGCTA	
AARS_1803	GCTGTGTGGTTGCTCATGAT	
AARS_631	CCAGGACGACCCTAATGTGC	
AARS_837	CAGGTGCCCGACCATACACT	
ADCK1_266	GAGCTCTGCTGTGCCAACCG	

ADCK1_459	GCTTCGATGACACCCCTCTG
ADCK1_826	GGTCAATGACAGAGACTACA
ADCK1_935	TCCGCACCAGTACATTGCCG
AIMP2_342	TTCAGCGCCCCGTAATCCTG
AIMP2_472	TTCAGGCACGCTCTTGACCG
AIMP2_539	TGAATCCCAGCTGATAGTCT
AIMP2_70	GTTGGGGAGCCGGTACATGC
AMZ2_153	GATGGAGAATGCAAGGTAAT
AMZ2_278	AGCATTTATATACAGTCCAT
AMZ2_606	TGGGGATATTCAGCTTTGCC
AMZ2_62	CATACTGTGATACAAGCACT
ANKRD26_1457	GGCATGCCAGTAGCCCACAT
ANKRD26_1922	AAGGCCAGTTTACTAACTGG
ANKRD26_2289	ACAAGGTTAATGTACTACAA
ANKRD26_2624	AATGCCAGAATGTTACAAGA
ATG10_211	ACAGACATGTCTTCCCATGG
ATG10_330	CTTGCCCTAAAGTAAAGTAC
ATG10_41	TGAATTCTGCACAATAACGT
ATG10_430	GCGACTGCTACAGGGACCAT
BCDIN3D_132	AGGAGGCGGAGCCGTTGCTC
BCDIN3D_215	CCGATTCTGGGGCTCGACGT
BCDIN3D_46	GAGTGTTAAGGAGACCGCAG
BCDIN3D_535	TCATGGAGACCATGGCCTAT
BOLA1_164	GAGCTTCGCAACGAGAGCGG
BOLA1_230	GTGGTGAGCTCTCGTTTCGA
BOLA1_255	TGAGCCCCCTACAACGACAC
BOLA1_88	GGGATCCGGGGCCATCGGTC
BRCA2_2782	TGTGTCTCCATATAAAACCA
BRCA2_3514	GTCTACCTGACCAATCGATG
BRCA2_573	GAACTTGACCAAGACATATC
BRCA2_5956	AGCAAGTGGAAAATCTGTCC
C11orf1_113	AATCTGTCCACACTTCACCA
C11orf1_158	TGGTGCATCGCCATCCATAC
C11orf1_193	CATCAGGGTACGGTTTGAAT
C11orf1_267	TAGATTACCTGGGAAGGCAA
C12orf66_632	GTAAATTAACCAGAGATGGG
C12orf66_736	GTTTGGAGGGCAGTCTCAGA
C12orf66_77	TGTCGTAAGAGAAGATACCC
C12orf66_805	ATGAAAGTAAAAGCTAAACT
C1orf74_113	GCACCTCTCCAGCTAAGTGA

C1orf74_307	TGAGCATGTATGTCAGCACT	
C1orf74_438	AGATCATCACACATTTGCAG	
C1orf74_516	GGAATCTGTGTACTGTATTT	
CCDC71_197	CATAGACATCACCACTGCGC	
CCDC71_357	AGTCTGCCAGATAGCGGCAT	
CCDC71_493	GTAGACACCTGGATAAAGGT	
CCDC71_862	GGCCAAGGTAGCTCGAACAC	
CETN3_168	AGGTGGCAATGAGAGCCTTG	
CETN3_230	GATTATGACAGAGAAGCCAC	
CETN3_280	TGTAGTGACAGACTGGATAT	
CETN3_310	TCCCCATGAAGAAATACTCA	
CHAC1_158	GCCTACAGCGACAGCCGTGT	
CHAC1_212	CAGGGAGACACCTTCCATCG	
CHAC1_296	GGCGTGGCATACCAAGTGCA	
CHAC1_367	GCTTGGTGGCTACGATACCA	
COQ5_113	ACAAGAGCCGAGCACTTAGT	
COQ5_175	CTTTGGGTTTGAGACTGTGT	
COQ5_371	ACTGAACATAATTAAGGAAC	
COQ5_487	CACGACACGAGACCCGCCCA	
CPSF3_1077	TAGCGGGATACTGTGTAGAA	
CPSF3_379	GCTGTATACCGAGACAGATT	
CPSF3_506	ATGTTCATGATTGAGATCGC	
CPSF3_804	AAAGATGATGCATAGTATAT	
CRADD_143	GAAATCAATGCTCAAACCAC	
CRADD_187	GCTGGATATCCTACCTTCCA	
CRADD_226	TACATTCCTAGATTCCCTAC	
CRADD_348	TCAACAGCTCCCCATCAGAC	
DHRS12_235	GATCAATAATGCAGGTTGCA	
DHRS12_317	GGTGTGTACATTCTCACGAC	
DHRS12_361	ATAAGGTCAACTCACCACTC	
DHRS12_412	TCTTTCGGACTGGAGATCAT	
DNAJA3_173	TCAATGTCAGCAGCGCTCGG	
DNAJA3_290	GAAGATTATTATCAGATATT	
DNAJA3_373	GTATCACCCTGACACAAATA	
DNAJA3_608	GAGGCTGATCAAACACGGTC	
DUS4L_252	GCAGCAAACTGAACAATCAA	
DUS4L_338	AATGGAATAGACATTAACTG	
DUS4L_405	ACCATGTCTTGAACAAGCTC	
DUS4L_591	TTAATGGAATCATAGTGCAC	
ELAC1_290	TAAAGTCCCGAAGCCCTACA	

ELAC1_353	CCAGTTCATGAACCACATAA
ELAC1_558	ACGACTGAAAACCCAAATGA
ELAC1_67	ACACCGAAGGACCACAGCAG
ERCC4_1141	AGAAAGCAACCCAAAGTGGG
ERCC4_1691	GTACCCTTGTCAGAGCATAG
ERCC4_647	TGGTAGGTGTCATAGAAACA
ERCC4_830	ATTTAGTCTTGGCTCCAAGC
ERI2_1212	TCCTCCCAATCAGCCAACAC
ERI2_556	TGATCTCAGAGCAACTTACA
ERI2_727	AATTACAAGGTCGTTGAACA
ERI2_838	GGGTCCCAGCATATATAATA
ERLIN2_205	GACCACACTCCAGACAGATG
ERLIN2_293	GTTAAGACGTACCTGCGTTC
ERLIN2_365	AGAACTGGTTCAGTTCGTGG
ERLIN2_80	GCTGTGCACAAGATAGAAGA
ETAA1_1081	ATGTGTGACTTCCTGTACTA
ETAA1_1636	AAAAAGATCAGTATTAACAG
ETAA1_218	TGCAGTAAAAGTAACCCCGA
ETAA1_909	CTGGTGCTCCAAAAAGCCTC
EXOSC8_113	AGAACCACAACTGTCAACAT
EXOSC8_176	GGAAATACTACAGTAATCTG
EXOSC8_246	AGGGGTGGTAGATCCACATT
EXOSC8_310	AATGAACTGGCTAGCCACTT
FAM131A_131	GAGTGGATCGAACTTCCTCG
FAM131A_286	CCGGCGAGCCCTAACTATCC
FAM131A_466	GCCCTCGCTGAGGTCTGAAT
FAM131A_559	GCTCCGAGCATGGTCTTCGG
FAM72A_267	GAAAGAAGACAGGAACTACA
FAM72A_300	CCTGCAACAACGGACACTTC
FAM72A_369	CAGGTGTAAACGTCCTACTT
FAM72A_371	CTGGCAAGTTGCCCCAAAGT
FAM72D_277	GTTGTTGCAGGAAAGAAGAC
FAM72D_300	CCTGCAACAACAGACACTTC
FAM72D_350	ATTAACAGACTAGACTCCAC
FAM72D_371	GGTGTAAACGTCCTACTTCG
FAM86C1P_21	CTGAGGCCCCGCCCCCGTCA
FAM86C1P_417	GACCAGGACTGTGATGCCGT
FAM86C1P_643	TGATATCTGCACCTAATGAG
FAM86C1P_844	TGTAGAATTGAGGAGCCCGC
FAN1_1254	CTGGTCAGAAGTTATATGTA

FAN1_1441	CAAGTGGAAGGTCTTGGCTA
FAN1_159	TGCCGGTTTAAGTCATATCT
FAN1_1806	AAGACAGAGATGATCTTATC
FARSB_289	AAAGGCTCCAGTGTATAAAC
FARSB_463	AACAATTAGGAAAAGAGCAC
FARSB_659	TATCATAGATAACTGGATAC
FARSB_781	CACGGGAACTGACTTTACTA
FBXO4_142	GGTGGCGCGTACGACCTCAC
FBXO4_291	CTGTAAGAGATCCAATTCTG
FBXO4_479	AAATCCAGCCGTCCTATGTA
FBXO4_641	TTGCCTCAGAGGCAGATTGA
GALE_306	TTAACTCTGTAATAATCCAG
GALE_405	GCAGCTCAGCCACTGTGTAC
GALE_467	GGTGGTTGTACCAACCCTTA
GALE_90	TGGAAGTTATCGATGACCAC
GSTZ1_226	CTCCTCTAGATACTCAATGA
GSTZ1_317	ATGATTTCTGACCTCATCGC
GSTZ1_49	AATTCGAACTCTCCATGAGC
GSTZ1_94	GAAAGGCATCGACTACGAGA
GTF2H5_30	TGAAAGGAGTGCTTATAGAA
GTF2H5_49	GTACAGCAGAAACTGCTTCA
GTF2H5_82	CTTGGATGAGTCCAATGCCC
GTF2H5_84	TGGATGAGTCCAATGCCCTG
HINT2_161	GGATCCGGGAGAAGATGGTT
HINT2_223	ATCACGGAACACAAGACACT
HINT2_261	GGAATGACCAGGAAGTGCAC
HINT2_291	GCCTGGCTAATCCGAGGAAT
HMGCL_275	GACCACACTGAAGTCTTGAA
HMGCL_460	GAGGTTTGACGCAATCCTGA
HMGCL_527	GGGAGATCTTCCCTTCATAA
HMGCL_607	CACACCAATGGTGTCCCCCA
HMMR_1306	AAGTAGTGCTGCTCATACCC
HMMR_275	CCTGTAGAAGAACACGAATC
HMMR_457	TAATGAACTACTAAAATCTA
HMMR_566	GGTATGATGGCTAAGCAAGA
IFT27_109	TTTCCAGAAAAGCTACACCC
IFT27_158	ACAGTGCCAGTTCCTGACAC
IFT27_250	ATAGACGAGACATAAGACAT
IFT27_64	ACTGCGGAAGATCTGTGCCA
IFT88_1043	AGTTAGTATGAGGATCATCC

IFT88_1593	TTACCTATGAGAAACTAAAT
IFT88_195	ACCCCATATCCAGTAGCTAT
IFT88_773	GAACTTGGTCTAATGCCATT
IL15RA_391	TGAGCTGGGAGATGAAGCTG
IL15RA_440	ACAACAGCAGCTATTGTCCC
IL15RA_512	AGCAGTCATGAGTCCTCCCA
IL15RA_81	AGCTCCCTACCCCGCGTCGC
IMPACT_180	GCTGTACCTGGGTATTCATT
IMPACT_516	GGAATGCCATGATCAATCGG
IMPACT_579	ACCTGTTTGGGACAAACCAC
IMPACT_67	ACACCACTCCTCGCCATAAA
ITGB3BP_104	ATAACTTATTCTCCAACAAC
ITGB3BP_167	GAAGAGCAAAAGCACAGAAA
ITGB3BP_298	GAAATTGTCAGAAGAAATCA
ITGB3BP_338	ACTTTCTTGTAGGCTTTGGA
KCTD18_182	ATTTAAATAGACGTCCATCA
KCTD18_297	TCAGACAGGCTGTATGGATA
KCTD18_663	ACCCGCCAGTAGCTAACACC
KCTD18_742	ACTTGGAAGCCTGCGTCACA
KIF21B_1423	GTTCTGGATCAGCGCACCAA
KIF21B_2398	ACAGTTTCAGATCCGAGCTC
KIF21B_652	TGAGCTCTGCACGTTCATCT
KIF21B_964	GGTGGTGCACGTTCCCTACA
LRRC23_327	AGGTGGGTGAGGTAGTTGAG
LRRC23_388	GTAGGGCAGTTCATTCATCT
LRRC23_488	GAAACCCTGAATCTCAAAGG
LRRC23_587	GCTTAGGAAGATTGATTCCC
MBTPS2_301	AAAAACGCTGATGCAGACTT
MBTPS2_537	GACATGGGATAGCAGCTATT
MBTPS2_731	AAAATGGCAAGAGAATTACT
MBTPS2_965	AAATATACACACCTCTAACT
MCTS1_159	ATCCTGTCAAAATAGTCCGA
MCTS1_262	AAGATTACTTCACAAATGTA
MCTS1_338	GGAGCAAATATCATGTGTCC
MCTS1_98	CAATTGATAGAGCAATTTCC
METTL21A_154	CAGGTATGTGGAAAGAACGA
METTL21A_274	AGGTGCTCATGTGACTATCA
METTL21A_328	TTGGATATGAGGAGGTAAGT
METTL21A_96	CAAACCACACGATCCAGATC
MIF4GD_192	GCTACGCCATCATTCAGGTT

MIF4GD_240	TCCGACGTGGACTCCTCAAC
MIF4GD_376	GTCATAGACAGGGTTCACCA
MIF4GD_82	CCTATCTCTACCCCTAGATC
MRPL24_250	AGTTATCCGGCAGCGAAACT
MRPL24_290	GGACAGCATTACCGCTACAT
MRPL24_359	TAGGATCCACAAGTTTGACC
MRPL24_393	AATCTCCACTCGATCTCAGT
MRPL40_132	TCTGGGAACTCATTCCCATG
MRPL40_274	TTTATCCAAGAACTTTAGAG
MRPL40_370	ACGCTCTTGCTGCTTGTACA
MRPL40_98	AAGACAACAATGACGCTCGC
MRPS10_186	AAACAGGGAGTACATACCAC
MRPS10_226	CATATTATATAAGCGCCTCT
MRPS10_259	AGGTCACGATAAGGCTGTAT
MRPS10_302	GTGCTTGCTGCTAAAGAACT
MTHFSD_117	TATGTCAGTACCTTAAAGTT
MTHFSD_205	AGTGGACCCTGATAAACCAC
MTHFSD_531	TCGTGGACGATGGTGACCAC
MTHFSD_676	GCCGTGACCCACCTTGAACC
MTIF2_281	AAGGTGGTAGAAGTATGGAT
MTIF2_427	GATCAAAGAAGTGATAACGA
MTIF2_849	TTATTTACGGCAAGGATAAT
MTIF2_976	AGTGCCTGTCTCCGCACTTA
NDUFV3_186	ATGTAGTGGAACCAAAGGAG
NDUFV3_306	GTGAATAGCACGCTGCCACT
NDUFV3_428	GATTCAGAAGCTCGTCAGGT
NDUFV3_908	GGGTTGTCTGCGCCACCGAA
NEK9_1295	GCTATCCGTCAGGTGTCATG
NEK9_1808	AAGATCCGTACCATTGCCCC
NEK9_392	ATTGAGCTGGAATATTGTAA
NEK9_799	GAAGATCGTGCAAGGAATTC
NFKBIB_168	TCGGCTACGTCACTGAGGAT
NFKBIB_344	GTGGAGAAGCTGTACGCAGC
NFKBIB_530	AATCGGGGTACAAGGCGACA
NFKBIB_632	GGATAACGGCCACGTGGAGT
NSMCE1_151	GAAGTCCTCCAACTTATCTA
NSMCE1_253	TGGGAGACCCATTTATGCGT
NSMCE1_338	AGTCAATAATCAGTTCCAGC
NSMCE1_88	CCATGGCGTGCTAGAGGAAT
NUDT12_165	TAATGTATGCGGCAAGGAAT

NUDT12_378	TTAGCAAAACACTACTGGAC
NUDT12_478	ATTGCCACCTAGAGTAACCA
NUDT12_682	ATCTATACCTAGAGCAAACC
NUDT6_119	TACGTGCGGAATCCGCCAGT
NUDT6_260	GTACAGCAATGGCGATCAGA
NUDT6_311	AAGCAGCAGGGGCAATAAAT
NUDT6_428	GGATATGCTTCACATCAAGT
NUP37_113	GGAAACCTAATTGCATATGG
NUP37_209	ACACTTCGAACATTTCACCA
NUP37_448	AACATAAGCAACATACCTGC
NUP37_595	AAGAGATAAAATAGCCTGTT
OMA1_294	TTACCAGCAAATGTACTGTA
OMA1_656	CTGGAAGTAAGTCCAATCAC
OMA1_850	AGGGATCTCTCAGATCAATT
OMA1_999	AAATAGCACATGCAGTACTT
PAAF1_252	ATTCTGGAAAAAGTAGTATA
PAAF1_477	TGGATGCCCAGCTGAAGATA
PAAF1_553	ATTCCTCCCCCGATCAACGA
PAAF1_701	GCTGACAACTCCATAAACCT
PAN2_1575	ATGCAGTTACAGTAGGCGTT
PAN2_1780	GTCAGCCAGGATTAGACCGA
PAN2_2167	CTGTGAAAAGTACCAGCCCA
PAN2_513	GCAGCACTCTACTCGTTGGT
PARP3_1027	GCTGCCAGTCTGTTCTAAGT
PARP3_463	GTACACACTTATCGAAGTAC
PARP3_791	ACCGTCATCCCGCACAACTT
PARP3_945	AGCTGGTAGTCTCGGTCCAG
PIGK_163	TCGATAATTAAACCAGAATC
PIGK_370	AGTGGATTATAGAAGTTACG
PIGK_541	AGCATCCGCGAGTTCTATGT
PIGK_92	GCCGCTAGTCATATCGAGGT
PIGO_1121	TATGAAGAAATCGGGACACC
PIGO_1839	CAGCCACAACAAACCCCCCA
PIGO_2020	GTGGTATGGAGCTTGTGTGG
PIGO_368	TAGGAGGGTCAACCTGAGAT
PNPLA4_117	AGGATGTCAAAGCCTTCGCT
PNPLA4_270	ATGACTTCATGGCCCGACTA
PNPLA4_325	GGATACGTGCAGTCGGTTCT
PNPLA4_472	GCTAGTGGAATACAAAGGGC
POC1A_273	AAGTGTACGACTCACACATT

POC1A_431	AGCGGACCCAGTTGATATGC
POC1A_533	AATACGAGTGGACACATTCC
POC1A_719	TGATCAGGTAGTTTCCCGAC
PRIM1_284	CAACACAATACAGTGAAGCT
PRIM1_390	GTCATGAGGGTCCAGCACTT
PRIM1_499	AAGGAGAGGTGTTCATTGTT
PRIM1_92	TACTATCGCTGGCTCAACTA
QRSL1_297	ACTACTGTAGCATTATAAGG
QRSL1_559	TGTATCTGATCCTAAAGCCC
QRSL1_669	ACATCCATCGAATTCACCAG
QRSL1_844	ACTATGTATAGGAATTCCAA
RAB23_183	AAGATGTCAGACTAATGTTA
RAB23_236	ATTACAAAGGCCTACTATCG
RAB23_277	CGTGTTCTCTACCACAGATA
RAB23_89	ATGATTCAGCGATATTGCAA
RAD51B_160	TGTCCATGAACTTCTATGTA
RAD51B_243	AGGGTAGTAGATAAGAATGC
RAD51B_310	GGCTTGTGGATCCCTCACAG
RAD51B_391	TCCTTCTAATCCTCCCATGT
RFT1_1051	ATCCAGAGCCAGCTGAGAAT
RFT1_144	TCGTTGGCGTAGTAAATGTA
RFT1_336	TGAGGGACAACATTAGGATC
RFT1_574	ATAGCAGAGCACCAGAACTG
SETD1A_1762	TGAGCCACCCCGGTCGTCGT
SETD1A_2179	TAGAGCATACGGCAAGCCGT
SETD1A_2878	GCACCGCAAGTCCTTTGCTC
SETD1A_352	TATGTGCCGTAAGTACGGTG
SPA17_203	ATGCATGATTGTTATAGAAG
SPA17_280	AGAGTCTCAGATATCTGGGA
SPA17_38	ACCCACTACCGAATTCCACA
SPA17_99	GCAAAAGCTGGTATATTGTC
SRBD1_1053	TCAGTCTGCTATCGTACATT
SRBD1_1490	TACAGAGAAGAGGATAAATA
SRBD1_1726	TTGTGGACAAGGCTTCCGAG
SRBD1_675	AGAGAACTAATATTGAACCT
STOML1_164	AACTGATGAGGCCATGACAG
STOML1_252	ACAATCATCCGCTCGTAGGT
STOML1_433	CCAGATGCGAAACTGGACAT
STOML1_593	ATCAGCGACCAGCTTCTGGT
SUMF1_325	AGATGATCCTCAGATAAAGC

SUMF1_388	TTCAGTATTACTGACTTCAT
SUMF1_558	TACCTGTGAAAGGCGCTAAC
SUMF1_681	TATTCCCACTCAGCTTCCGT
TAF6_1110	GAGCCATAACGAGTCGTCCA
TAF6_1253	GCACATGGTCTGCTCCAATC
TAF6_774	GAGATAAAGGTACTGAACCG
TAF6_966	GCCCAGTGATTGTCCACATC
TATDN3_106	TGCCACAAGGGCCACAACAT
TATDN3_198	ACTGGATGAACACCCAAGCA
TATDN3_449	AATGTGCACTCACGCTCTGC
TATDN3_535	TGGTCGGCCATCTGTAGCCA
TAZ_123	ATCTTCTTCCGCCACGAGCT
TAZ_205	CGGGTGGCCGCCCGACGAGT
TAZ_563	GCAGTATCCCAGCCAAATCT
TAZ_751	AAGGATTCGAATGCGCCAAG
TCTN1_1142	GGAGCCCCACGACATAACCA
TCTN1_483	GGATTAATAAAGGATAATGC
TCTN1_707	TGCACTGATAATAACCCTGC
TCTN1_851	CGATGGACTGAACAGTGATA
TMEM143_257	CAGGCGGTACCTGTATTAGG
TMEM143_376	AGGGTTGATGGGGTCATATA
TMEM143_706	CCTCAGGAGATACTTTAAGC
TMEM143_884	GTGGCGATCTTCGTCAACGT
TMEM179B_123	AGGGTGGCCACACCATACAG
TMEM179B_187	CCCAGCTACAAAGTAGCACA
TMEM179B_283	AGGGTTAGGCAGTCACCTGT
TMEM179B_365	TCTGCCTGTATCCTTCGATT
TMEM209_194	TACTGGCCCCTCTGGTATAT
TMEM209_539	CGGGCGAGTAGGTCACTCCA
TMEM209_834	GGAACTATAGTCGTTCTATG
TMEM209_97	AGATACATTTAGGAGTCCCC
TMEM42_110	AGTTGAATACGCCCCAAAAG
TMEM42_134	GTATTCAACTGTCTGTGCGC
TMEM42_169	GCCGAAGGCCAGCTTGGCGG
TMEM42_244	GAAGGTCCACATCAGAGAAT
TRIM41_1158	ACATCAAGGAGACTTTCAAT
TRIM41_125	ACAACTGGGTTACACAAACT
TRIM41_256	GTAGTCTTCATCCCGCATGG
TRIM41_367	GGACAACATGGACTATGTGT
TRMT1L_1178	AAGTCACTGACACTATGCCA

TRMT1L_252	AGAGACACATCTCAATTCAA
TRMT1L_606	CTGATATGCTAGGACATGTT
TRMT1L_831	AAAGCATCTAGACATTCCAA
TTC12_1207	GAACAGTCCCATAGCAGTGT
TTC12_1318	CATTTAGAAGACAGATCCCA
TTC12_389	GCTATCCTGCGCTACAGTGA
TTC12_998	GGTGTATCACTAGCACACGC
TTC8_109	GGAGAAGTCCCCTTATGACC
TTC8_466	CAGCCTGACAAATCTTCCGG
TTC8_705	AGATTGGAAAATGTTACTAC
TTC8_988	GTTGCTTCCAATGCATGCGA
TUBGCP5_1291	TGATACTCGAAATGTCGTCC
TUBGCP5_1601	GATAACGCTAGTGCGAGTTC
TUBGCP5_1814	GTACAGTCCCGTCTTCGACA
TUBGCP5_262	TAGTTGGAAGAGATTAACGG
UROS_104	AAAACTCAAACGATAAAACA
UROS_217	GGAAGCAGCAGAGTTATGTT
UROS_518	TATCAGACAGTTGCACACCC
UROS_58	TGGCCAGGATCCGTATATCA
USP40_1429	TTTCCGATAAAACAACATGT
USP40_262	TAAGGATAAACCCGATGCAA
USP40_460	ATAGATGAGGTCATGACCGG
USP40_812	AAAAGGGCTTGAGATTAATC
ZNF414_165	GCCCCTCCACGTTCCCACAC
ZNF414_262	GCTGGTCCCGGAGACTATGC
ZNF414_426	GAGCAGCGGAAGAGCTTGCC
ZNF414_587	CATGCTCCGCGCAAACATGC
ZSWIM7_139	CTGTCGATCAACTAGGTCCA
ZSWIM7_196	CAGTGGAAGGCGTGTTTACC
ZSWIM7_267	AGCACTGAGAATGCAAATGC
ZSWIM7_84	TACGATAACAGATATTCATC

Table S2. PCR primers.

Name	Primer sequence	Source
SETD1A-RT-fwd	TTTGAGGATGTGGCACCTACAG	Hoshii T et al. *Cell Reports*, 2022
SETD1A-RT-rev	CAGAAGAGCATGGAGAAGCCT	
GAPDH-RT-fwd	TCATCAGCAATGCCTCCTGC	Hoshii T et al. *Cell Reports*, 2022

GAPDH-RT-rev	GATGGCATGGACTGTGGTCA	
TAF6-RT-fwd	CTAACGGATGAGGTCAGCTACC	PrimerBank (ID 298676519c1)
TAF6-RT-rev	AAGGCGTAGTCAATGTCACTG	
ACTB-RT-fwd	TGTCCCCCAACTTGAGATGT	Wang M et al. *Neuron*, 2021
ACTB-RT-rev	TGTGCACTTTTATTCAACTGGTC	
HBB-TSS-fwd	CAGGGTGAGGTCTAAGTGATGA	Hoshii T et al. *Cell Reports*, 2022
HBB-TSS-rev	TTGAAGTCCAACTCCTAAGCCA	
GAPDH-TSS-fwd	CCACTAGGCGCTCACTGTTCTC	Hoshii T et al. *Cell Reports*, 2022
GAPDH-TSS-rev	GAACTCACCCGTTGACTCCGAC	
TAF6-TSS-fwd	ACGGTTGGTTGTGTGTCTGT	
TAF6-TSS-rev	CACCCCATCTCCTGCAACAT	
RFT1-TSS-fwd	AGACGGTGGCATTGATGAGG	
RFT1-TSS-rev	TCACCCGGCCTAGACTCTAAA	

Table S3. Reagents.

Antibodies	Source	Catalog Number
Rabbit monoclonal anti-SETD1A	Cell Signaling	Cat#61702
Rabbit polyclonal anti-SETD1A	Abcam	Cat#ab70378
Rabbit monoclonal anti-GAPDH	Cell Signaling	Cat#2118
Rabbit polyclonal anti-Flag-tag	GenScript	Cat#A00170-40
Rabbit monoclonal anti-H3K4me1	Cell Signaling	Cat#5326
Rabbit monoclonal anti-H3K4me2	Cell Signaling	Cat#9725
Rabbit monoclonal anti-H3K4me3	Active Motif	Cat#39159
Rabbit polyclonal anti-Histone H3	Abcam	Cat#ab1791
Mouse monoclonal anti-Myc-tag	Cell Signaling	Cat#2276
Rabbit monoclonal anti-Cyclin D1	Cell Signaling	Cat#2978
Mouse monoclonal anti-p21	Santa Cruz	Cat#sc-6246
Rabbit polyclonal anti-TAF6	Sigma-Aldrich	Cat#HPA006566
Mouse monoclonal anti-E2F1	Thermo Fisher	Cat#32-1400
Rabbit monoclonal anti-E2F4	Cell Signaling	Cat#40291
Rabbit monoclonal anti-Rpb1-NTD	Cell Signaling	Cat#14958
Rabbit monoclonal anti-phospho-Rpb1 CTD	Cell Signaling	Cat#13499
Rabbit monoclonal anti-H3K36me3	Abcam	Cat#ab9050
Spike-in antibody	Active Motif	Cat#61686
PE anti-mouse/human CD44 Antibody	BioLegend	Cat#103024
Chromatin	Source	Catalog Number

Spike-in chromatin	Active Motif	Cat#53083
Recombinant DNA	Source	Catalog Number
Plasmid: pCW-Cas9	Addgene	Cat#50661
Plasmid: pLKO5.sgRNA.EFS.GFP	Addgene	Cat#57822
Plasmid: pLKO5.sgRNA.EFS.tRFP657	Addgene	Cat#57824
Plasmid: pLEX305.degTAG.BSD	Hoshii T et al. *NAR*, 2024	

Cell line STR Profiles

July 18, 2024

BEX CO., LTD.
2-61-14 Itabashi Itabashi-ku, Tokyo, Japan
TEL : +81-3-5375-1071
FAX : +81-3-5375-5636

Cell Line Authentication

Client : Ryoji Fujiki
Department of Molecular Oncology, Graduate School of Medicine, Chiba University

Analysis conducted by Katsunori Imai

Sample : AGS

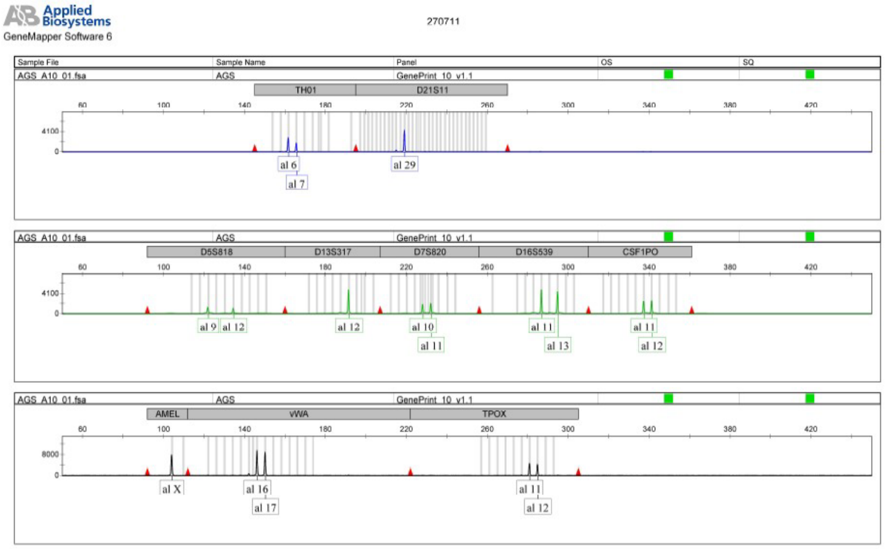

STR Profile

Locus	AGS	
TH01	6	7
D21S11	29	
D5S818	9	12
D13S317	12	
D7S820	10	11
D16S539	11	13
CSF1PO	11	12
AMEL	X	
**vWA**	**16**	17
TPOX	11	12

**AGS (CVCL_0139)**	
6	7
29	
9	12
12	
10	11
11	13
11	12
X	
16	17
11	12

STR profiles of AGS and AGS (CVCL_0139) were completely matched. It was verified that the cells analyzed were considered to be the same as the cells registered in Expasy by comparison with the database of Expasy.

Sample : SNU-719

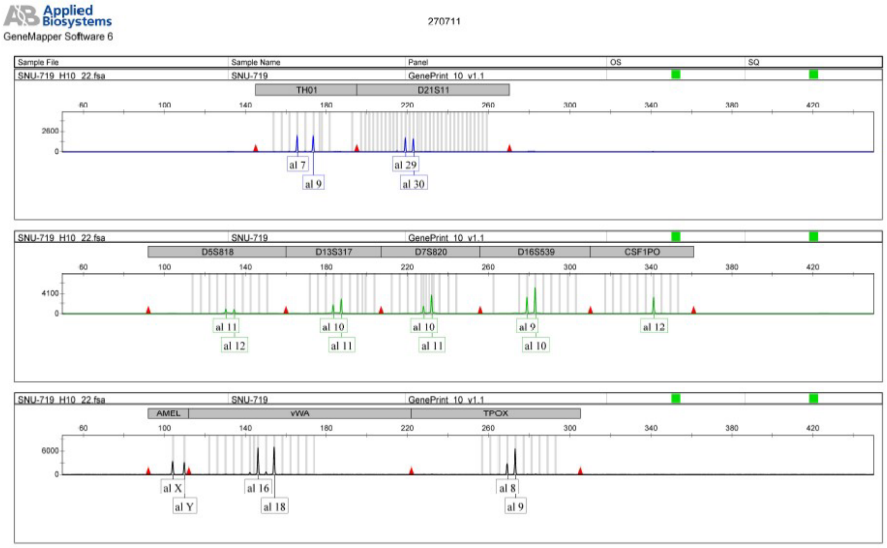

STR Profile

Locus	SNU-719		SNU-719 (CVCL_5086)	
TH01	7	9	7	9
D21S11	29	30	29	30
D5S818	11	12	11	12
D13S317	10	11	10	11
D7S820	10	11	10	11
D16S539	9	10	9	10
CSF1PO	12		12	
AMEL	X	Y	X	Y
**vWA**	**16**	18	16	18
TPOX	8	9	8	9

STR profiles of SNU-719 and SNU-719 (CVCL_5086) were completely matched. It was verified that the cells analyzed were considered to be the same as the cells registered in Expasy by comparison with the database of Expasy.

Sample : MKN45

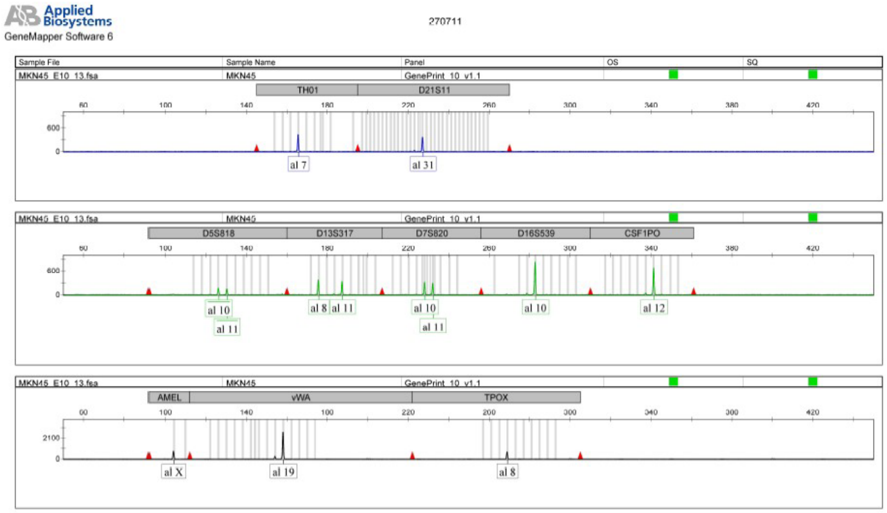

STR Profile

Locus	MKN45		MKN45 **(CVCL_0434)**	
TH01	7		7	
D21S11	31		31	
D5S818	10	11	10	11
D13S317	8	11	8	11
D7S820	10	11	10	11
D16S539	10		10	
CSF1PO	12		12	
AMEL	X		X	
**vWA**	**19**		19	
TPOX	8		8	

STR profiles of MKN45 and MKN45 (CVCL_0434) were completely matched. It was verified that the cells analyzed were considered to be the same as the cells registered in Expasy by comparison with the database of Expasy.
