# Supplementary material for: Non-catalytic role of SETD1A promotes gastric cancer cell proliferation through the E2F4–TAF6 axis in the cell cycle
Source: Cell Death Dis. 2025 Aug 23;16(1):639. doi: 10.1038/s41419-025-07976-4 (PMC12373860; doi:10.1038/s41419-025-07976-4)
Supplement: Supplementary file 2 — Original Data [file 41419_2025_7976_MOESM2_ESM.pdf]

Full and uncropped Western blots

Fig. 1C

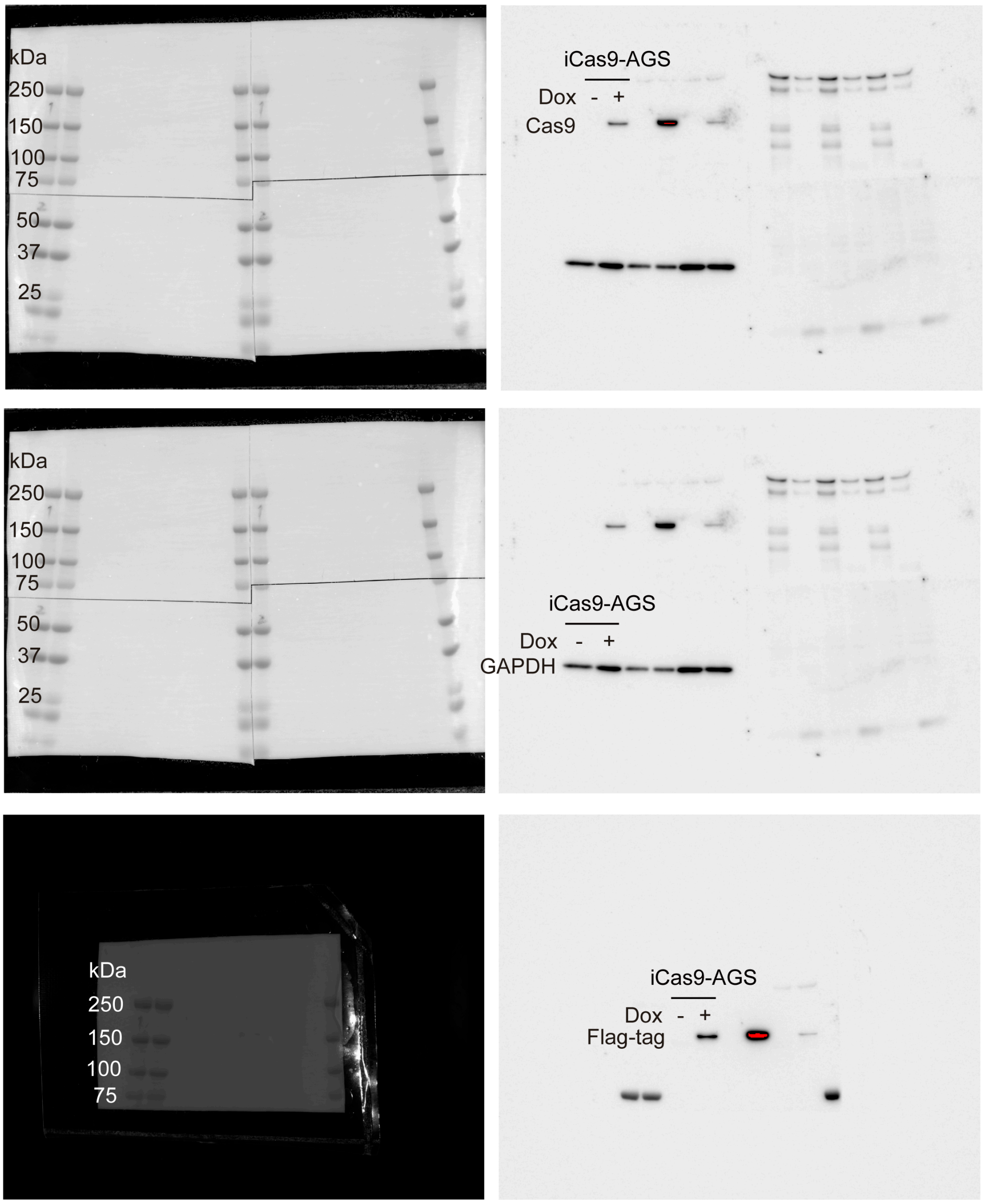

Fig. 1D

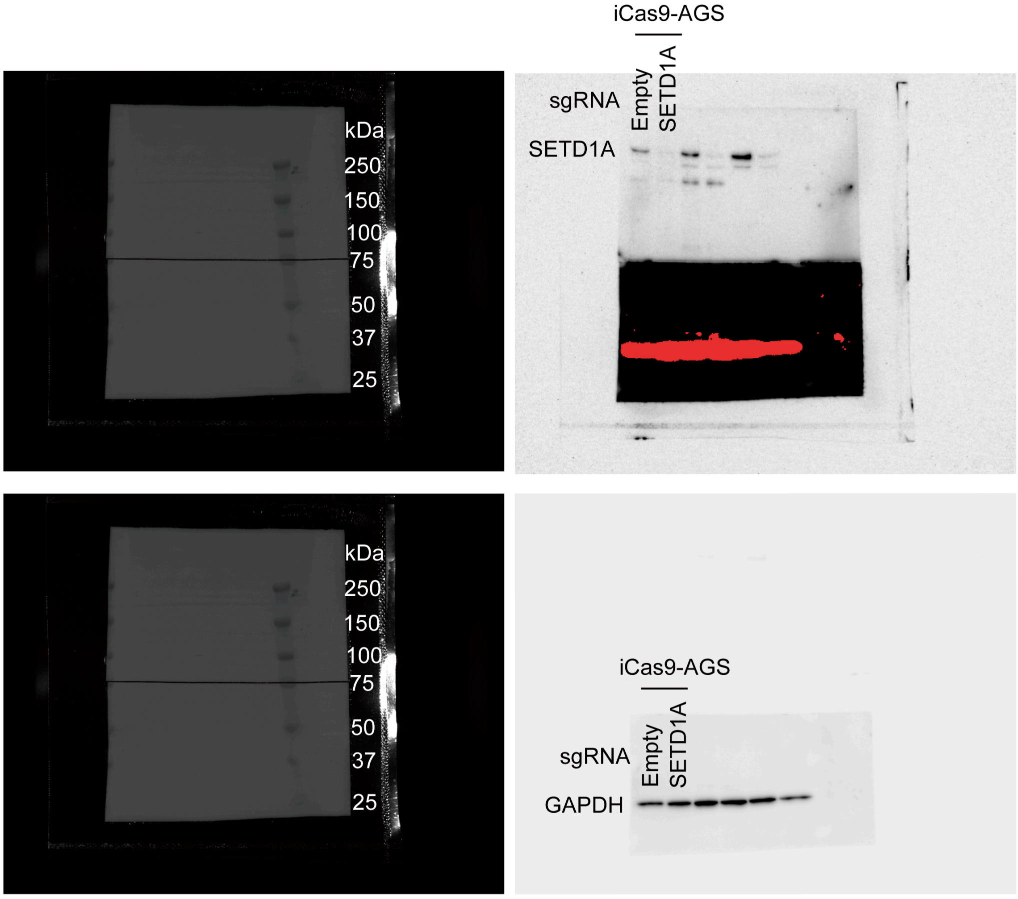
kDa
250
150
100
75
50
37
25
iCas9-AGS
sgRNA
Empty
SETD1A
SETD1A
kDa
250
150
100
75
50
37
25
iCas9-AGS
sgRNA
Empty
SETD1A
GAPDH

Fig. 1I

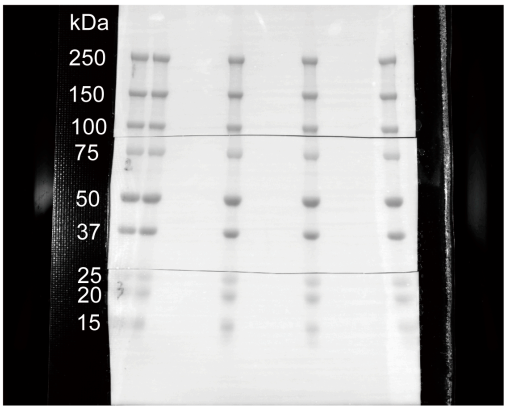
kDa
250
150
100
75
50
37
25
20
15

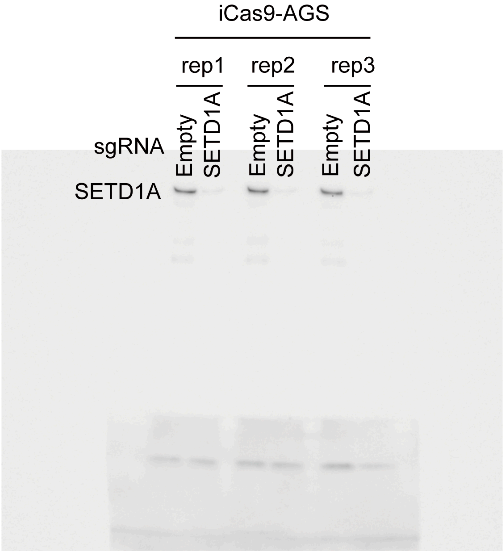
iCas9-AGS
rep1
rep2
rep3
sgRNA
Empty
SETD1A
Empty
SETD1A
Empty
SETD1A
SETD1A

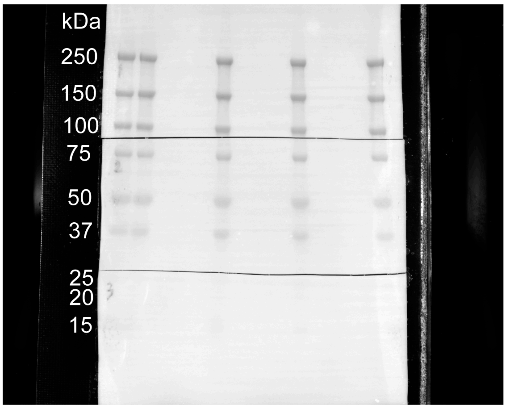
kDa
250
150
100
75
50
37
25
20
15

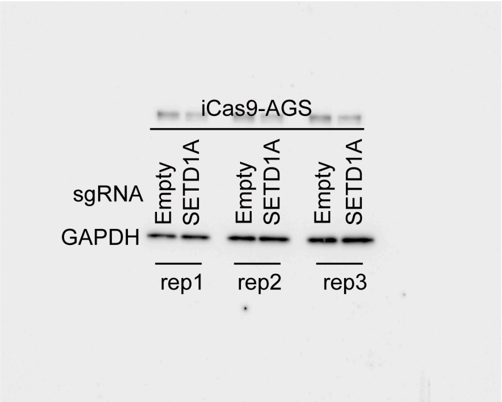
iCas9-AGS
sgRNA
Empty
SETD1A
Empty
SETD1A
Empty
SETD1A
GAPDH
rep1
rep2
rep3

Fig. 1I

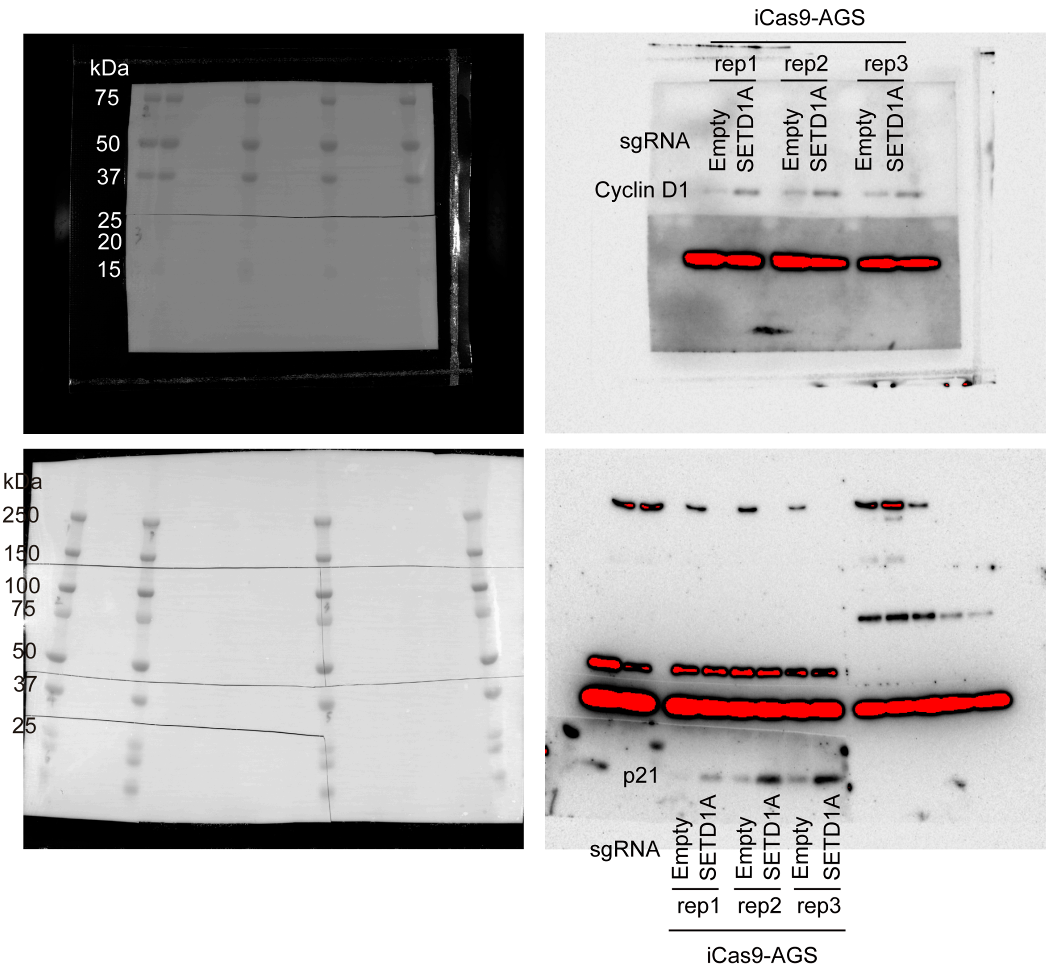
iCas9-AGS
kDa
75
50
37
25
20
15
rep1
rep2
rep3
sgRNA
Empty
SETD1A
Empty
SETD1A
Empty
SETD1A
Cyclin D1
kDa
250
150
100
75
50
37
25
p21
sgRNA
Empty
SETD1A
Empty
SETD1A
Empty
SETD1A
rep1
rep2
rep3
iCas9-AGS

Fig. 2B

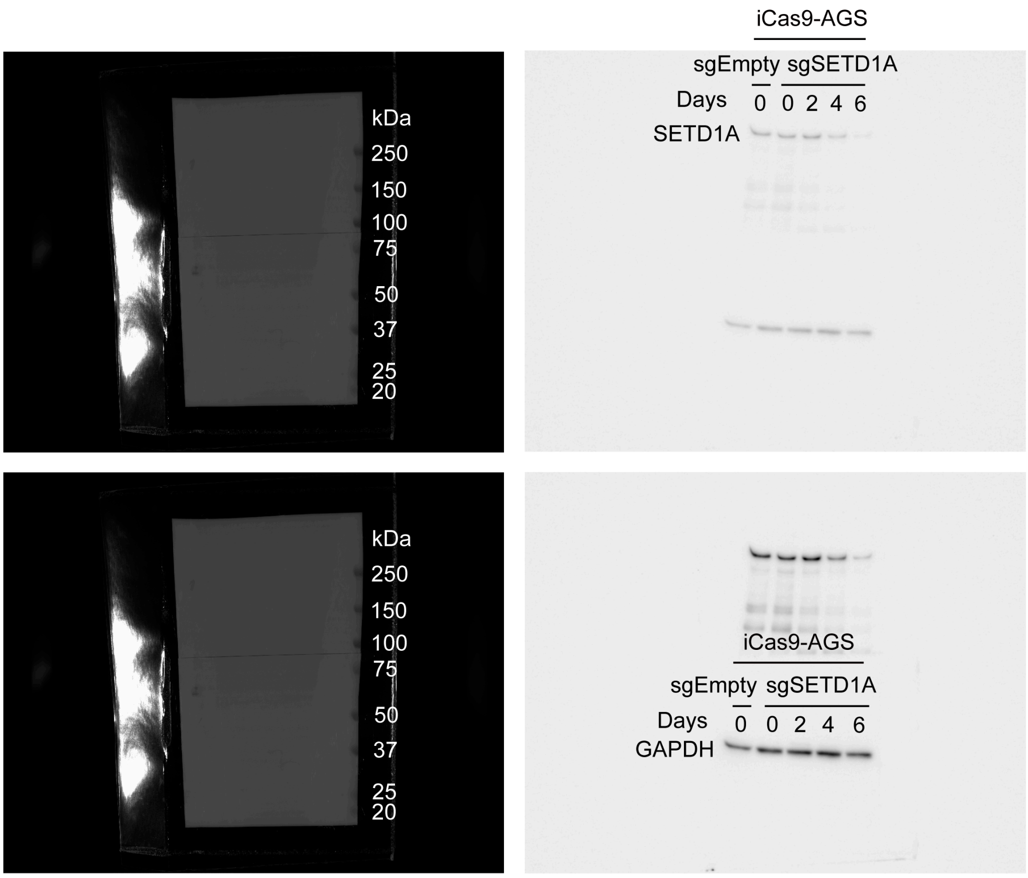
iCas9-AGS
sgEmpty sgSETD1A
Days 0 0 2 4 6
SETD1A
kDa
250
150
100
75
50
37
25
20
kDa
250
150
100
75
50
37
25
20
iCas9-AGS
sgEmpty sgSETD1A
Days 0 0 2 4 6
GAPDH

Fig. 2D

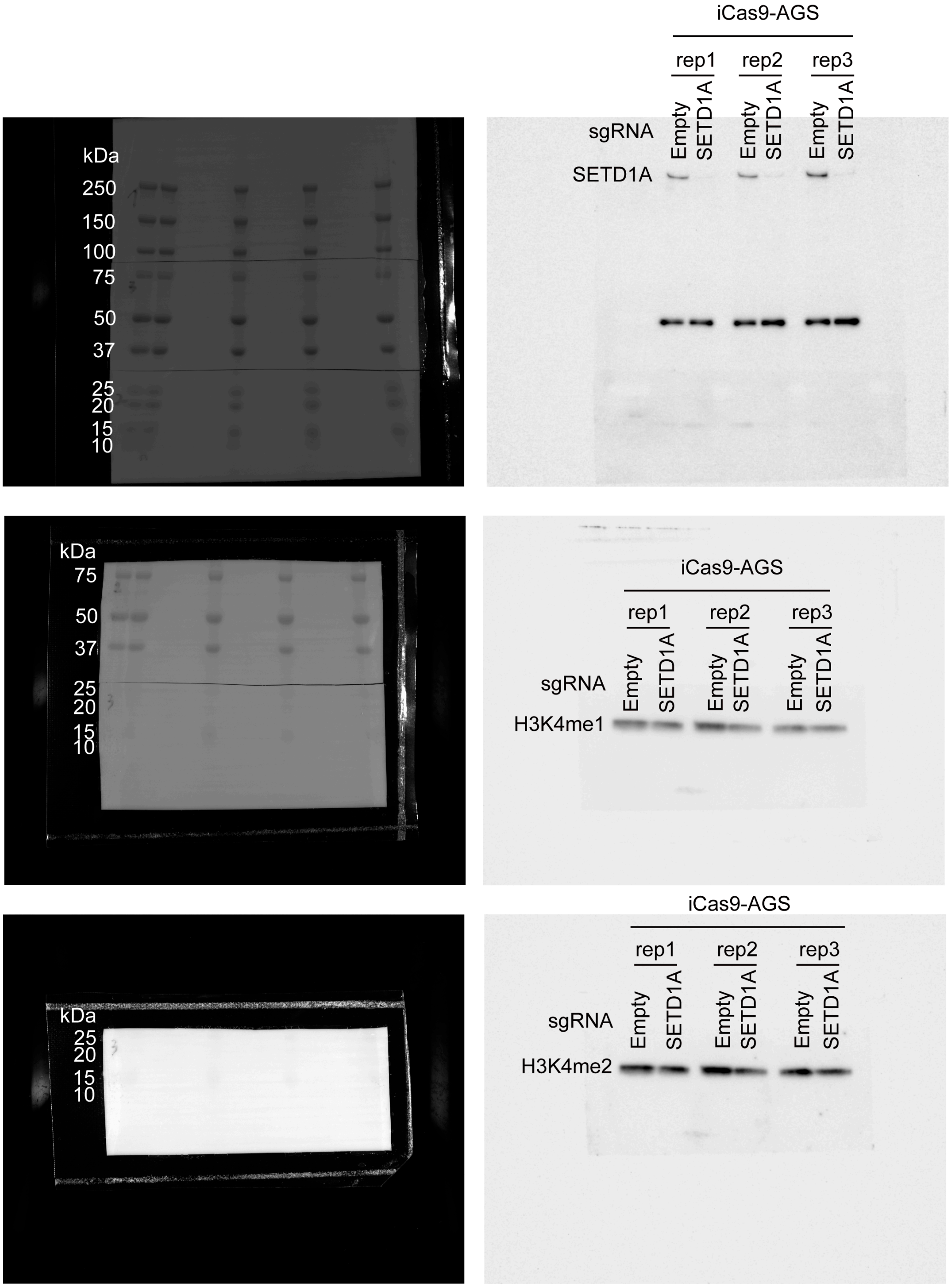
kDa
250
150
100
75
50
37
25
20
15
10
iCas9-AGS
rep1
rep2
rep3
sgRNA
Empty
SETD1A
Empty
SETD1A
Empty
SETD1A
SETD1A
kDa
75
50
37
25
20
15
10
iCas9-AGS
rep1
rep2
rep3
sgRNA
Empty
SETD1A
Empty
SETD1A
Empty
SETD1A
H3K4me1
kDa
25
20
15
10
iCas9-AGS
rep1
rep2
rep3
sgRNA
Empty
SETD1A
Empty
SETD1A
Empty
SETD1A
H3K4me2

Fig. 2D

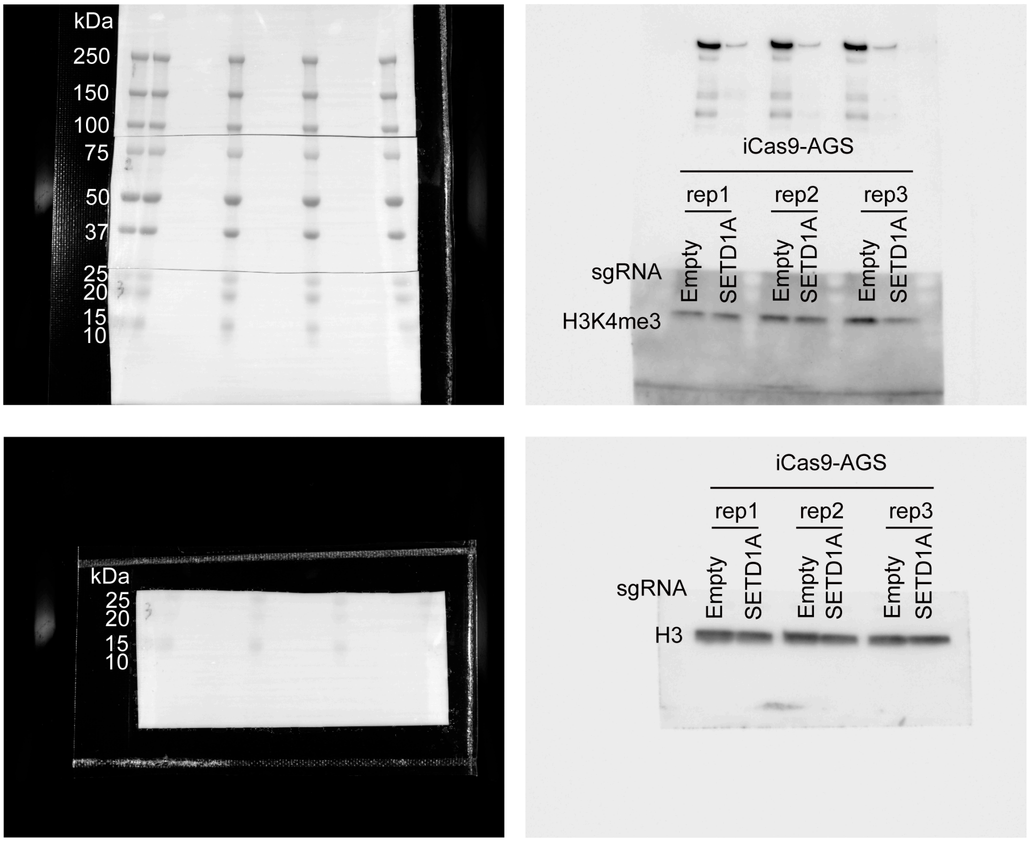
kDa
250
150
100
75
50
37
25
20
15
10
iCas9-AGS
rep1
rep2
rep3
sgRNA
Empty
SETD1A
H3K4me3
kDa
25
20
15
10
iCas9-AGS
rep1
rep2
rep3
sgRNA
Empty
SETD1A
H3

Fig. 4F

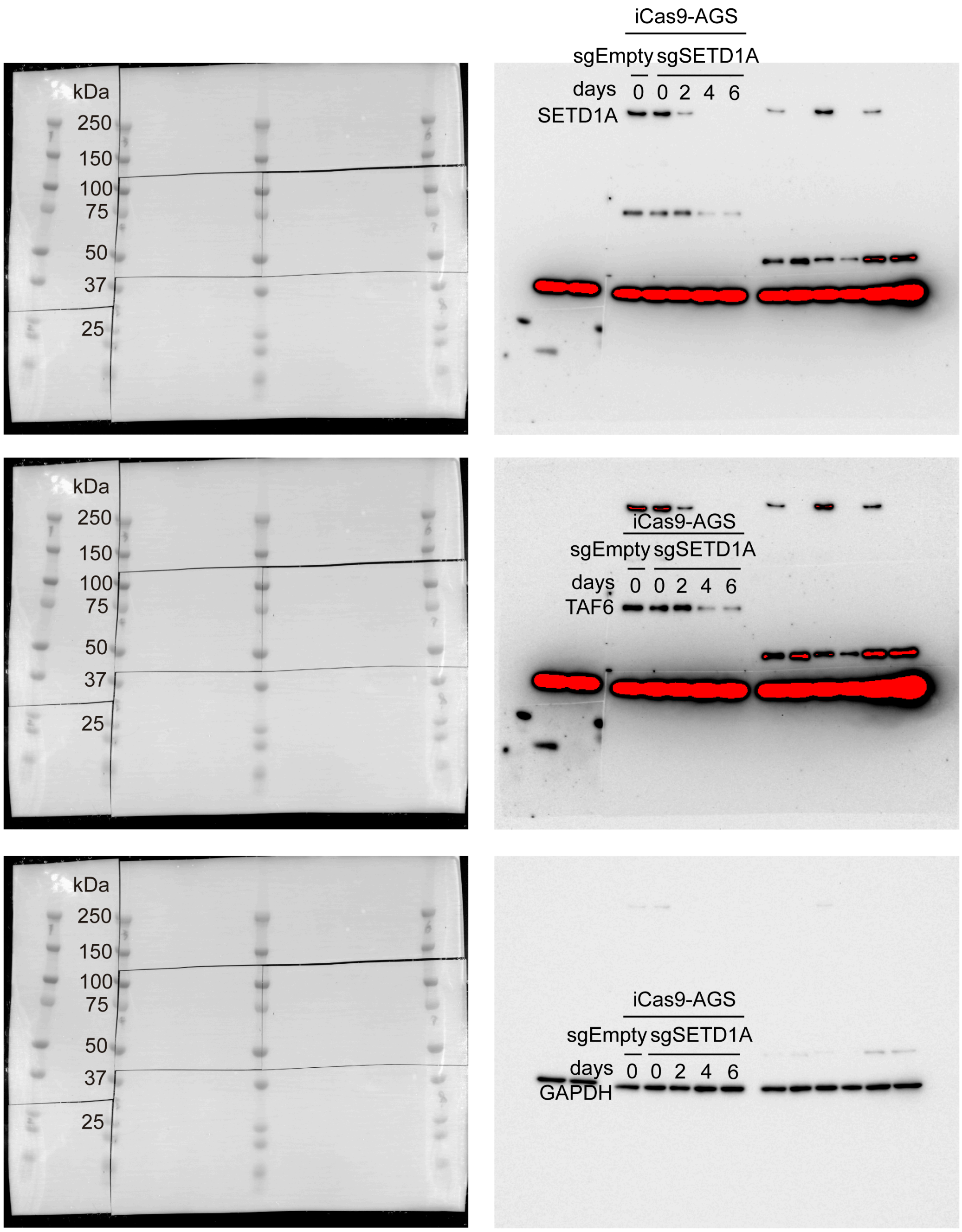
iCas9-AGS
sgEmpty sgSETD1A
days 0 0 2 4 6
SETD1A
kDa
250
150
100
75
50
37
25
iCas9-AGS
sgEmpty sgSETD1A
days 0 0 2 4 6
TAF6
iCas9-AGS
sgEmpty sgSETD1A
days 0 0 2 4 6
GAPDH

Fig. 4F

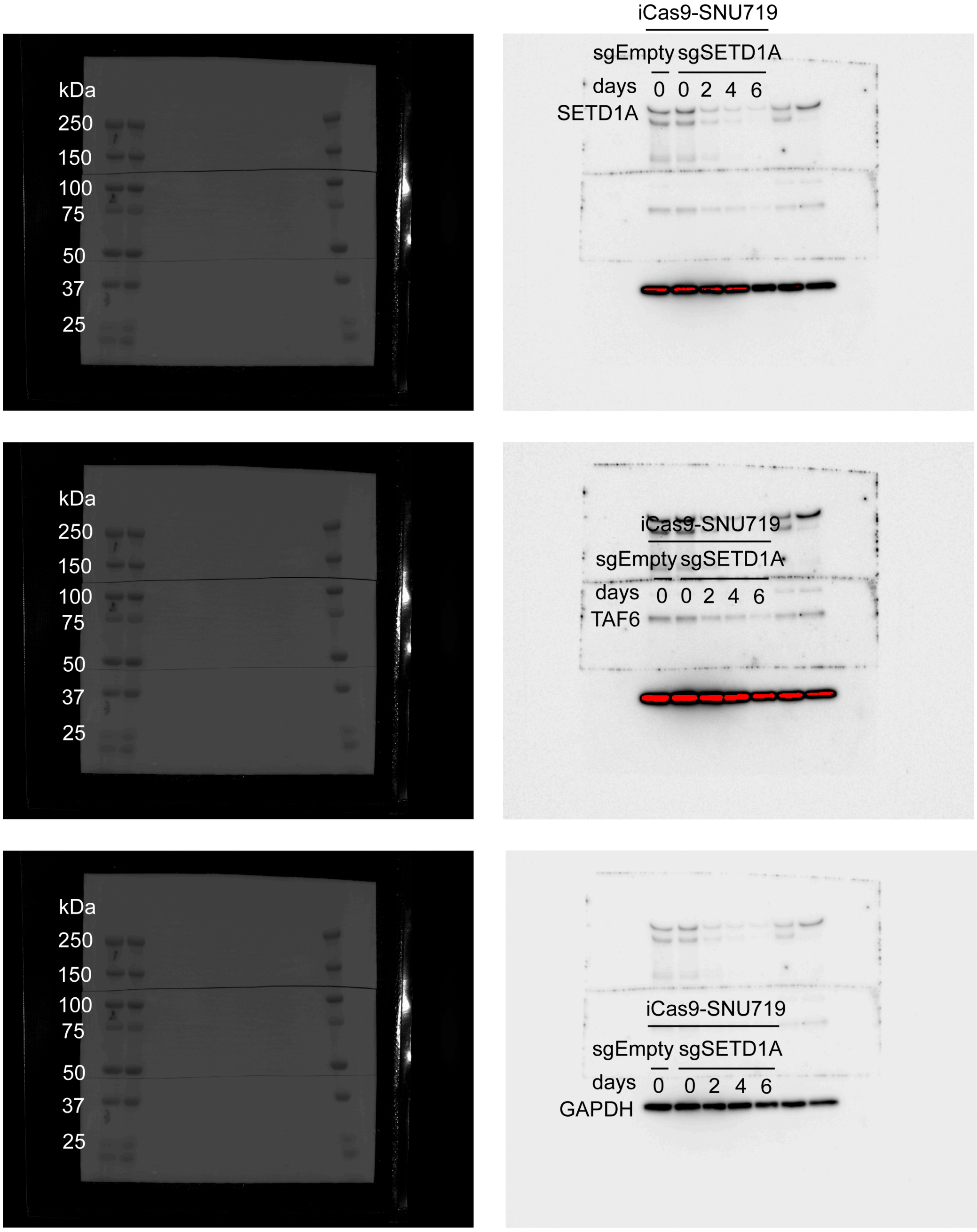
iCas9-SNU719
sgEmpty sgSETD1A
days 0 0 2 4 6
SETD1A
TAF6
GAPDH
kDa
250
150
100
75
50
37
25

Fig. 4F

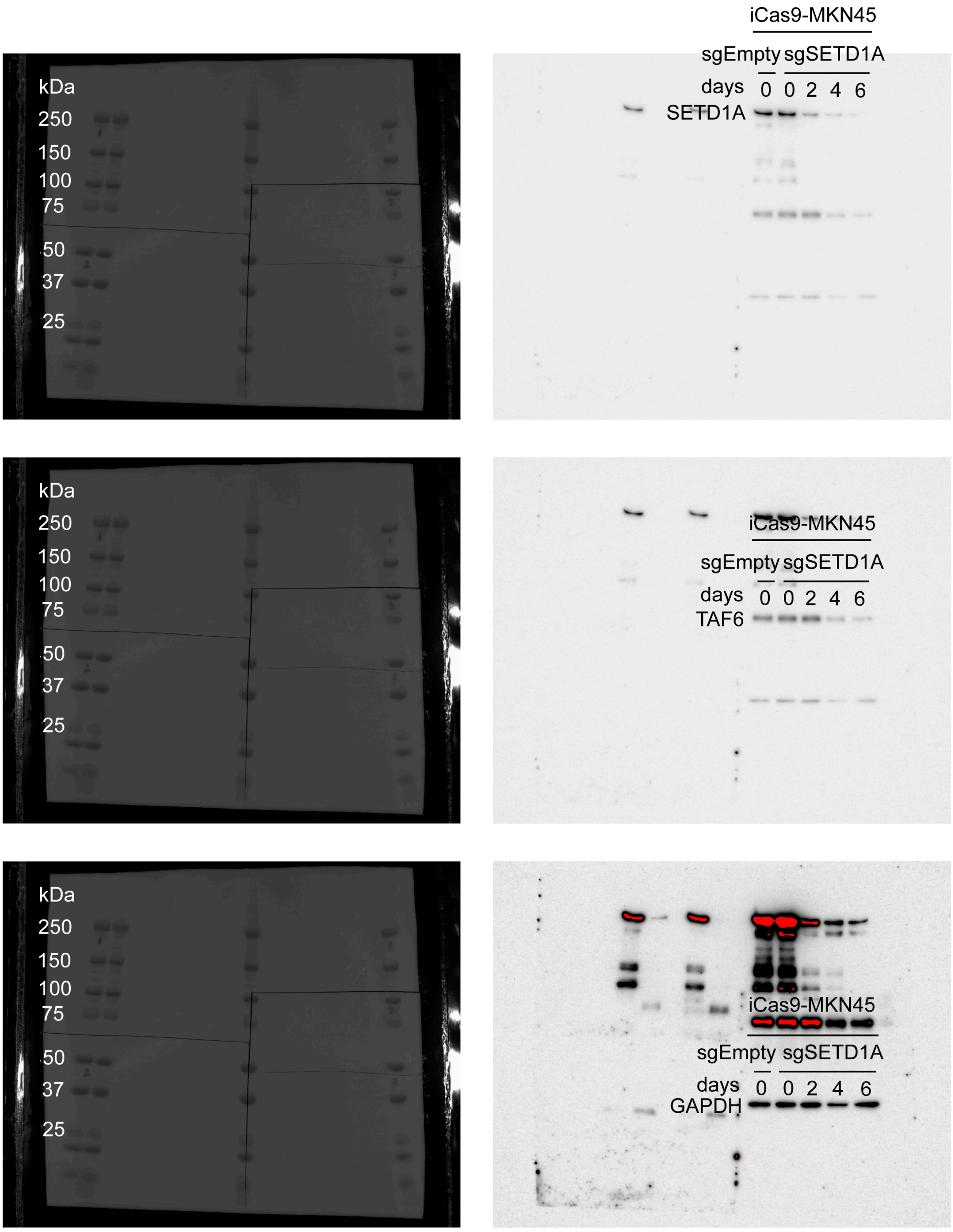
iCas9-MKN45
sgEmpty sgSETD1A
days 0 0 2 4 6
SETD1A
kDa
250
150
100
75
50
37
25
iCas9-MKN45
sgEmpty sgSETD1A
days 0 0 2 4 6
TAF6
kDa
250
150
100
75
50
37
25
iCas9-MKN45
sgEmpty sgSETD1A
days 0 0 2 4 6
GAPDH
kDa
250
150
100
75
50
37
25

Fig. 4H

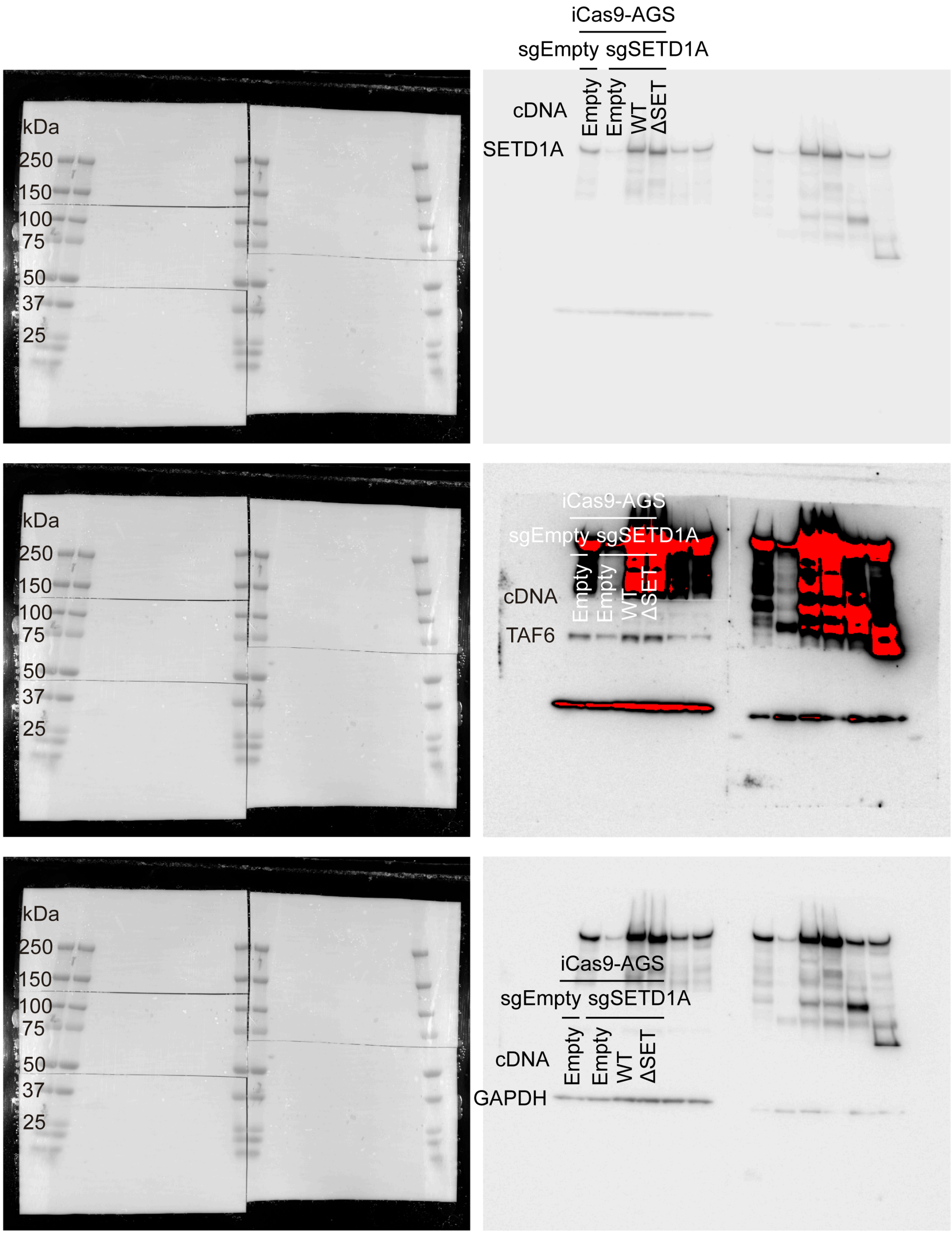
iCas9-AGS
sgEmpty sgSETD1A
cDNA
Empty
Empty
WT
ΔSET
SETD1A
TAF6
GAPDH
kDa
250
150
100
75
50
37
25

Fig. 5H

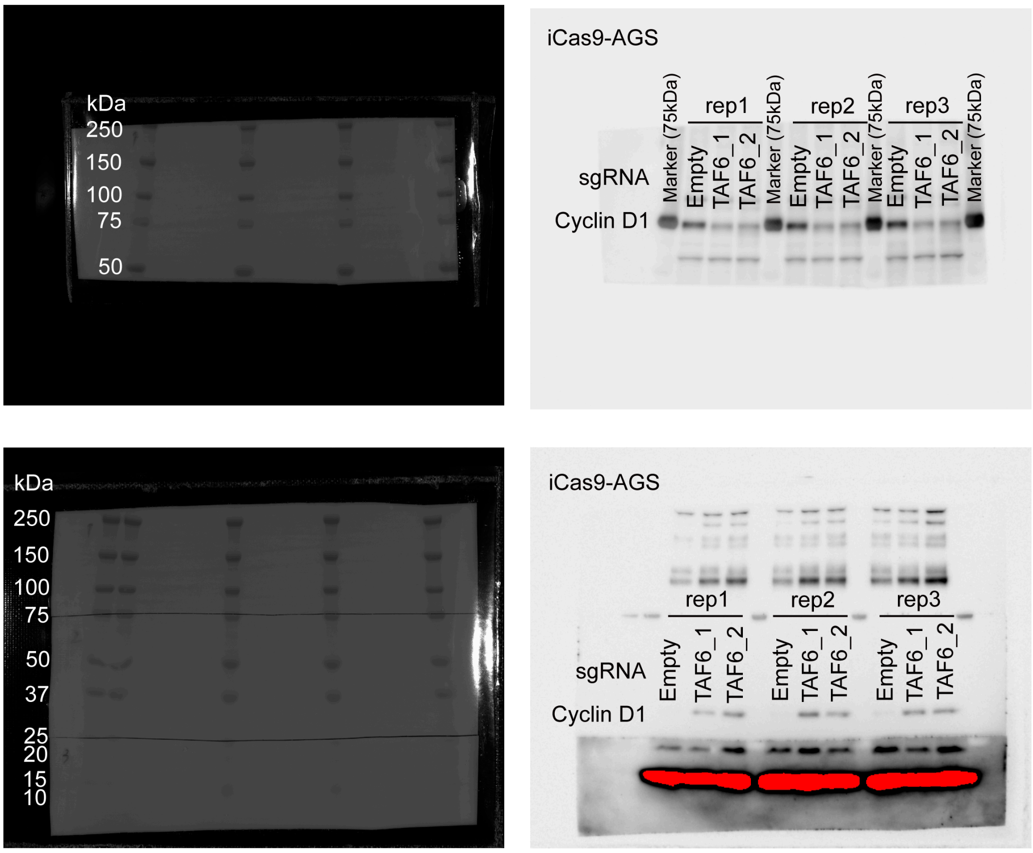

kDa
250
150
100
75
50
iCas9-AGS
rep1
rep2
rep3
sgRNA
Marker (75kDa)
Empty
TAF6_1
TAF6_2
Cyclin D1
kDa
250
150
100
75
50
37
25
20
15
10
iCas9-AGS
rep1
rep2
rep3
sgRNA
Empty
TAF6_1
TAF6_2
Cyclin D1

Fig. 5H

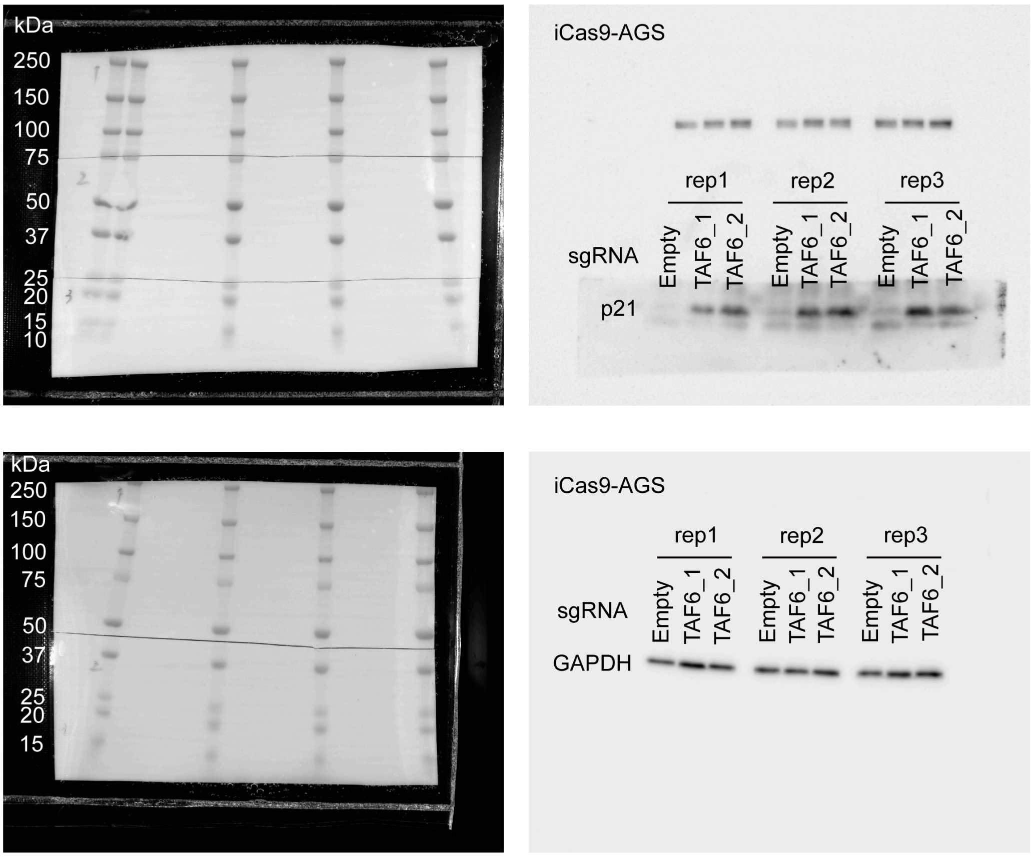
kDa
250
150
100
75
50
37
25
20
15
10
iCas9-AGS
rep1
rep2
rep3
sgRNA
Empty
TAF6_1
TAF6_2
p21
kDa
250
150
100
75
50
37
25
20
15
iCas9-AGS
rep1
rep2
rep3
sgRNA
Empty
TAF6_1
TAF6_2
GAPDH

Fig. 6C

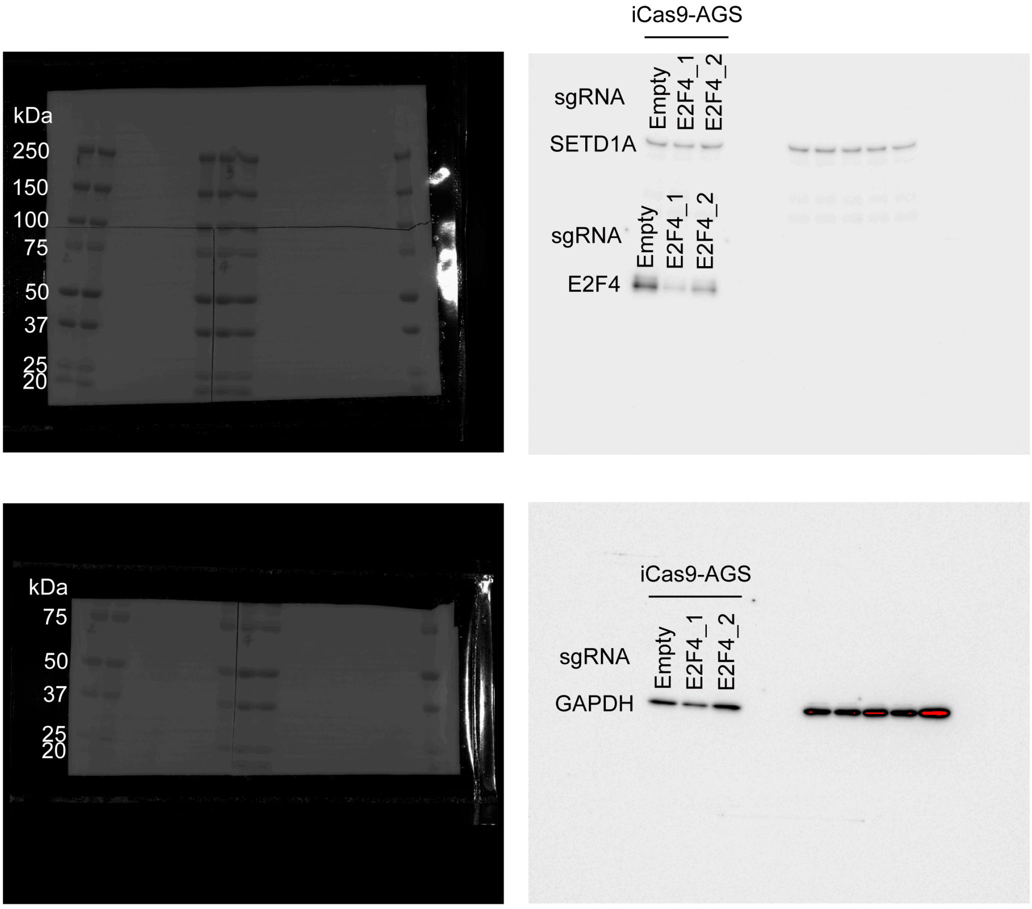
iCas9-AGS
kDa
250
150
100
75
50
37
25
20
sgRNA
Empty
E2F4_1
E2F4_2
SETD1A
sgRNA
Empty
E2F4_1
E2F4_2
E2F4
kDa
75
50
37
25
20
iCas9-AGS
sgRNA
Empty
E2F4_1
E2F4_2
GAPDH

Fig. 6E

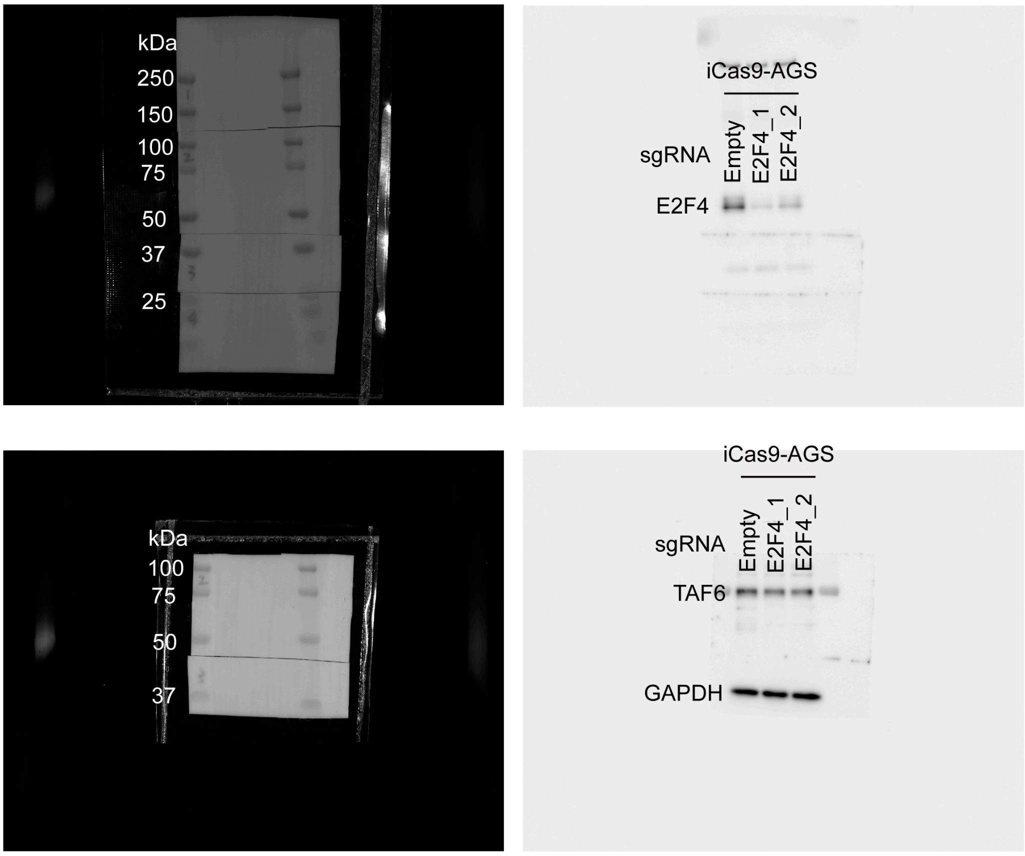
kDa
250
150
100
75
50
37
25
iCas9-AGS
sgRNA
Empty
E2F4_1
E2F4_2
E2F4
kDa
100
75
50
37
iCas9-AGS
sgRNA
Empty
E2F4_1
E2F4_2
TAF6
GAPDH

Fig. S1A

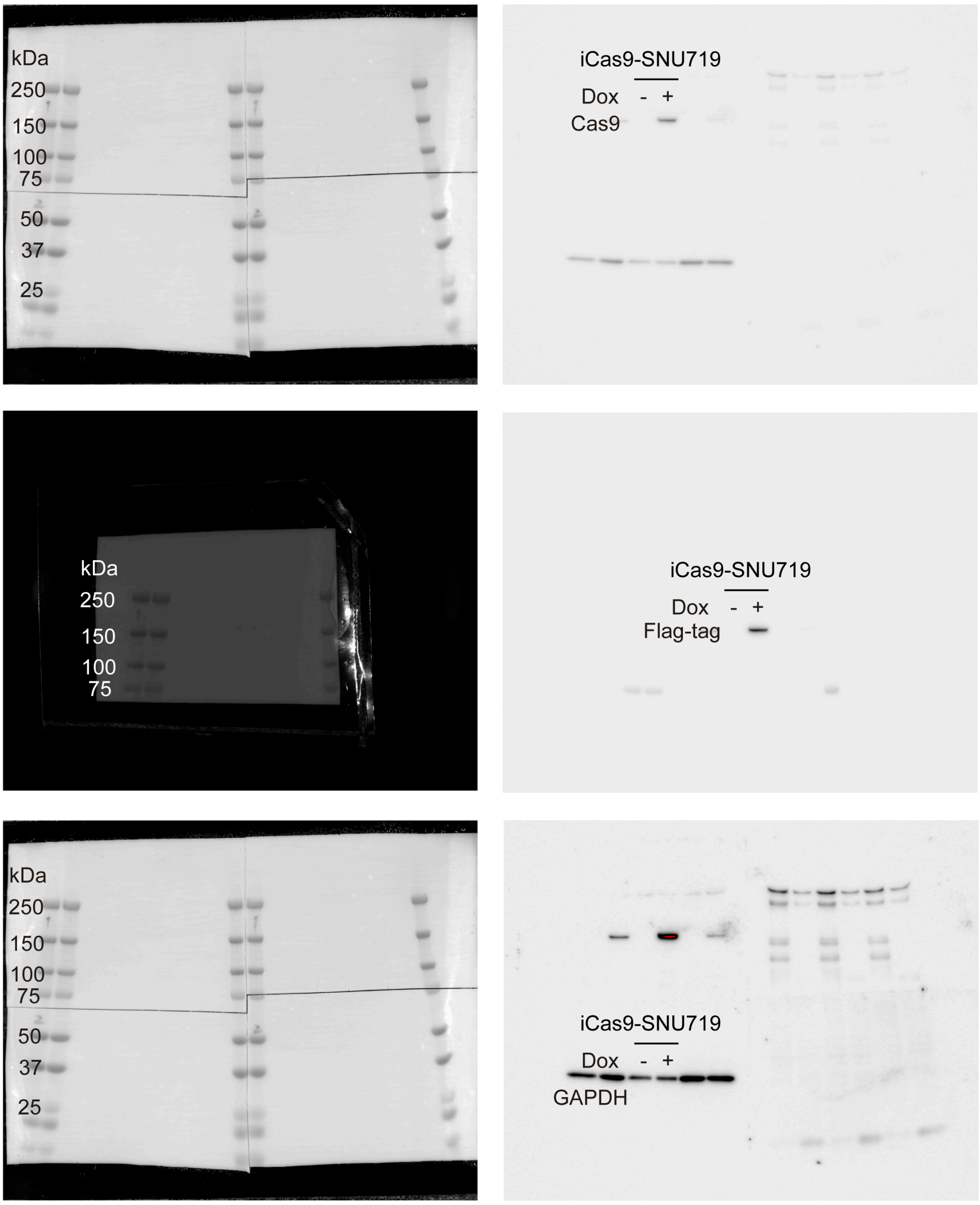
kDa
250
150
100
75
50
37
25
iCas9-SNU719
Dox - +
Cas9
kDa
250
150
100
75
iCas9-SNU719
Dox - +
Flag-tag
kDa
250
150
100
75
50
37
25
iCas9-SNU719
Dox - +
GAPDH

Fig. S1A

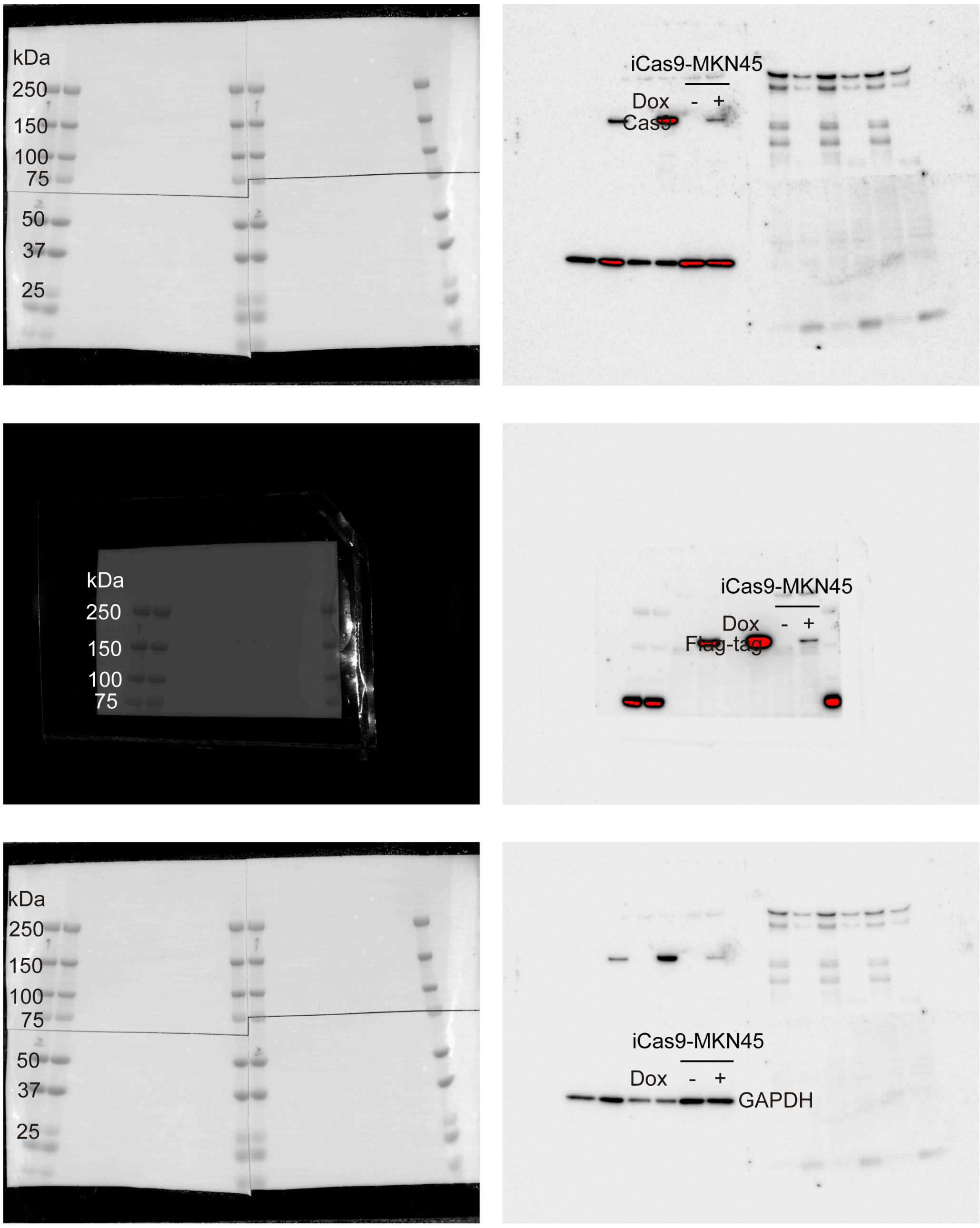
kDa
250
150
100
75
50
37
25
iCas9-MKN45
Dox
-
+
Cas9
kDa
250
150
100
75
iCas9-MKN45
Dox
-
+
Flag-tag
kDa
250
150
100
75
50
37
25
iCas9-MKN45
Dox
-
+
GAPDH

Fig. S1B

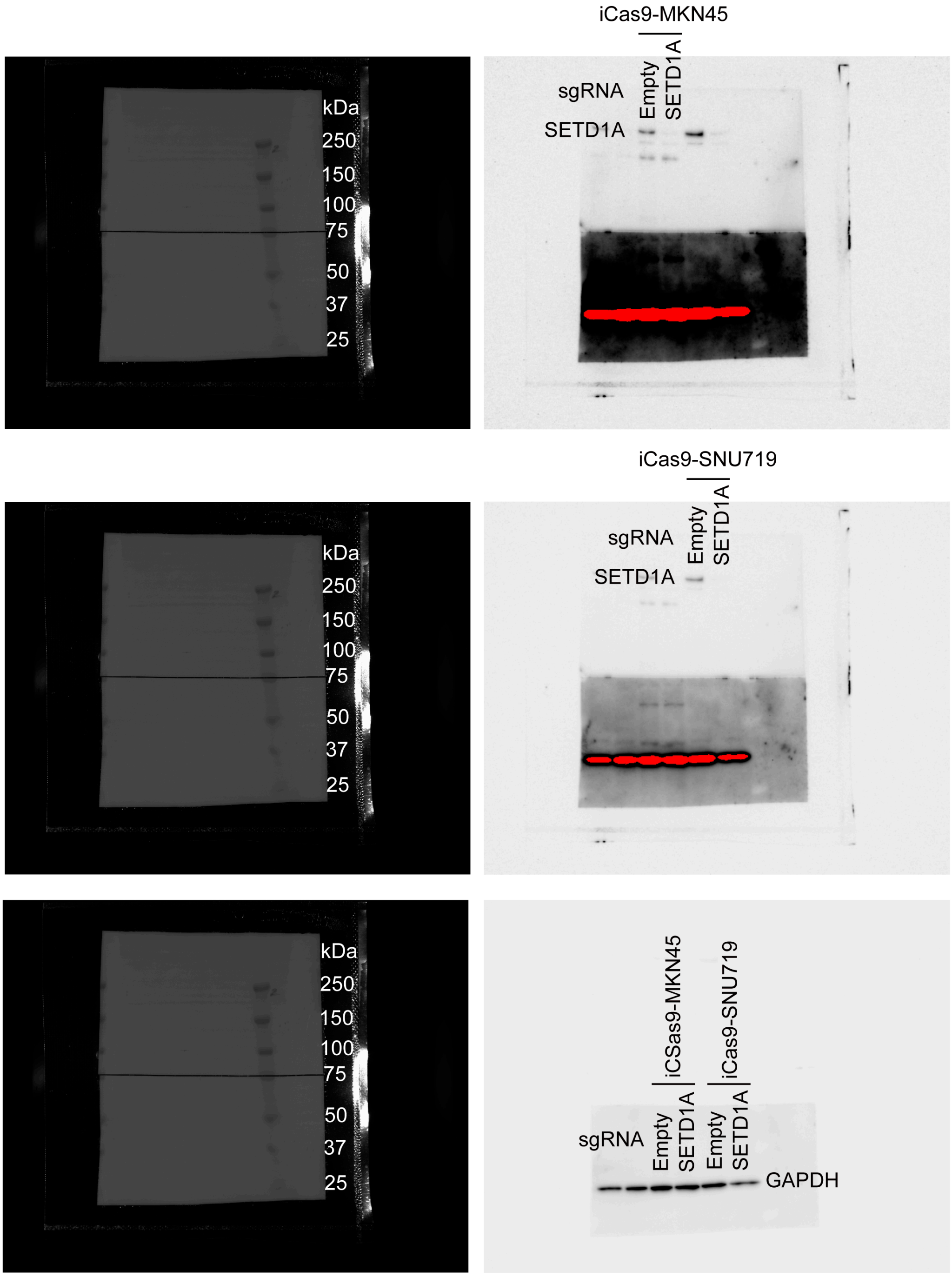
kDa
250
150
100
75
50
37
25
iCas9-MKN45
sgRNA
Empty
SETD1A
SETD1A
kDa
250
150
100
75
50
37
25
iCas9-SNU719
sgRNA
Empty
SETD1A
SETD1A
kDa
250
150
100
75
50
37
25
iCSas9-MKN45
iCas9-SNU719
sgRNA
Empty
SETD1A
Empty
SETD1A
GAPDH

Fig. S1F

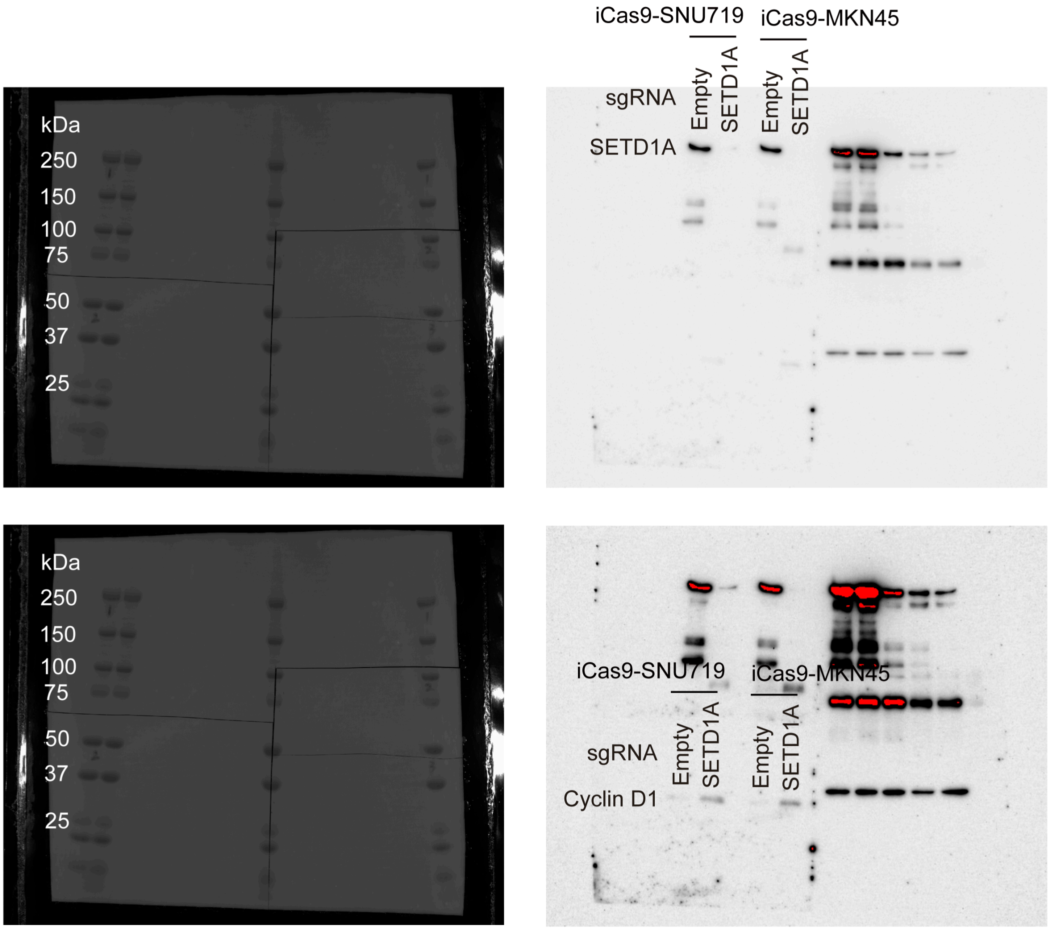
iCas9-SNU719
iCas9-MKN45
sgRNA
Empty
SETD1A
Empty
SETD1A
SETD1A
kDa
250
150
100
75
50
37
25
iCas9-SNU719
iCas9-MKN45
sgRNA
Empty
SETD1A
Empty
SETD1A
Cyclin D1
kDa
250
150
100
75
50
37
25

Fig. S1F

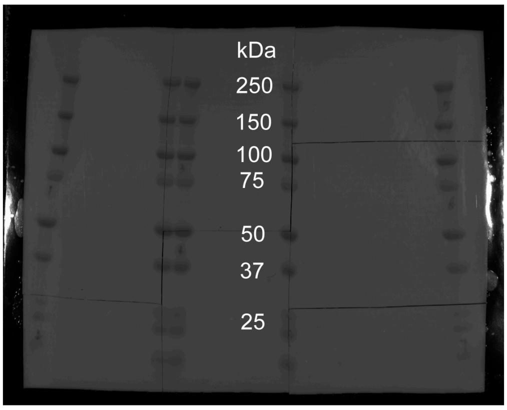

kDa
250
150
100
75
50
37
25

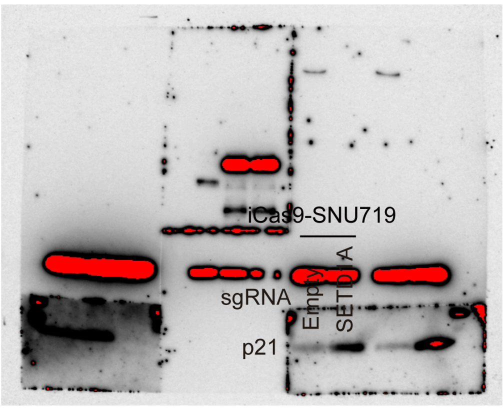

iCas9-SNU719
sgRNA
Empty
SETD1A
p21

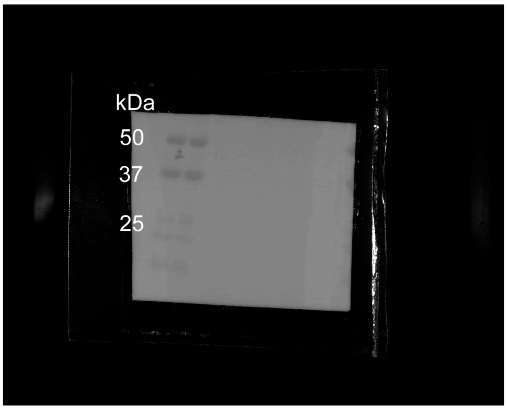

kDa
50
37
25

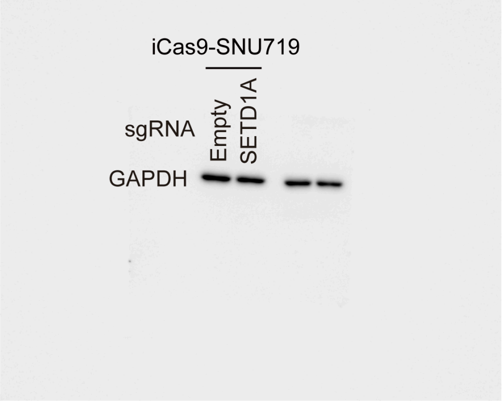

iCas9-SNU719
sgRNA
Empty
SETD1A
GAPDH

Fig. S1F

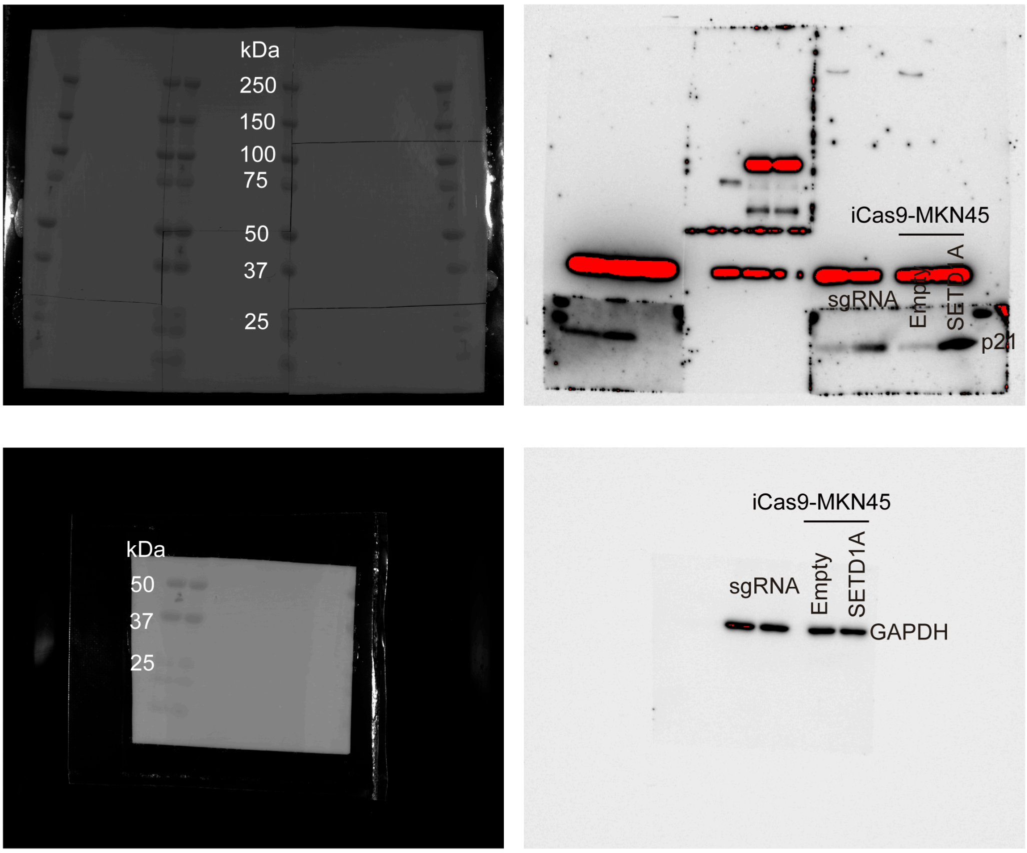

kDa
250
150
100
75
50
37
25
iCas9-MKN45
sgRNA
Empty
SETD1A
p21
kDa
50
37
25
iCas9-MKN45
sgRNA
Empty
SETD1A
GAPDH

Fig. S1G

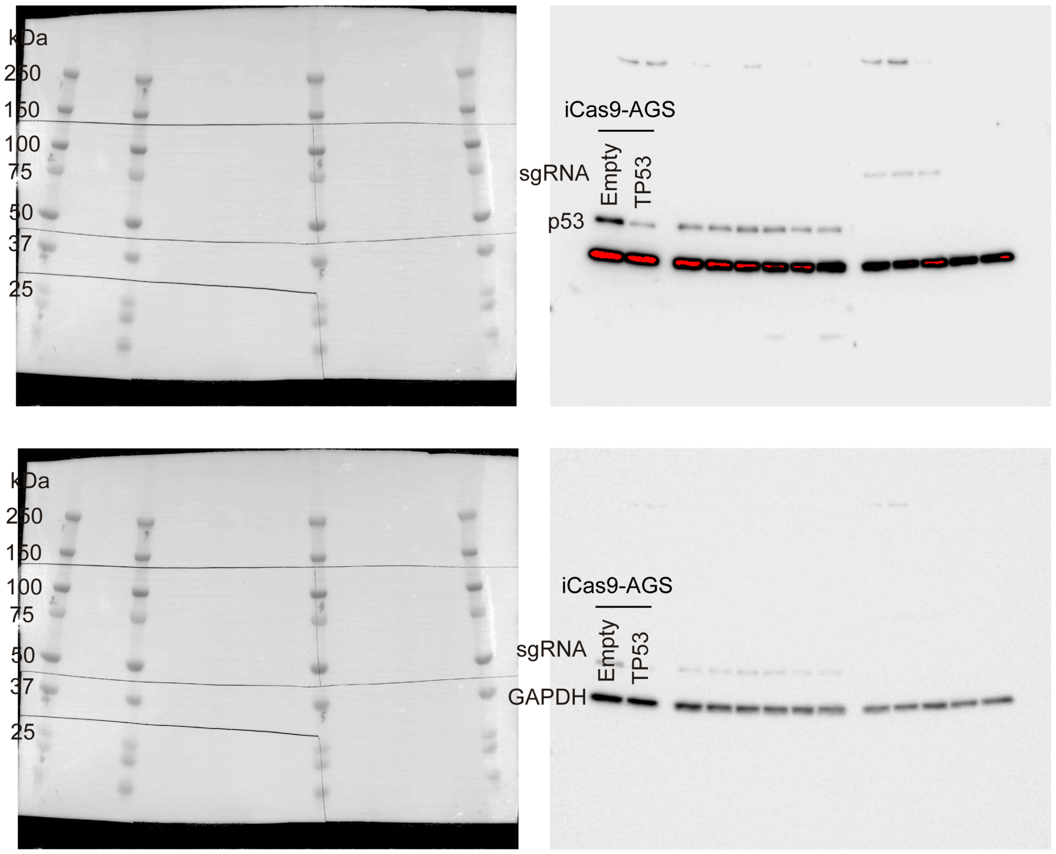

kDa
250
150
100
75
50
37
25
iCas9-AGS
sgRNA
Empty
TP53
p53
kDa
250
150
100
75
50
37
25
iCas9-AGS
sgRNA
Empty
TP53
GAPDH

Fig. S2B

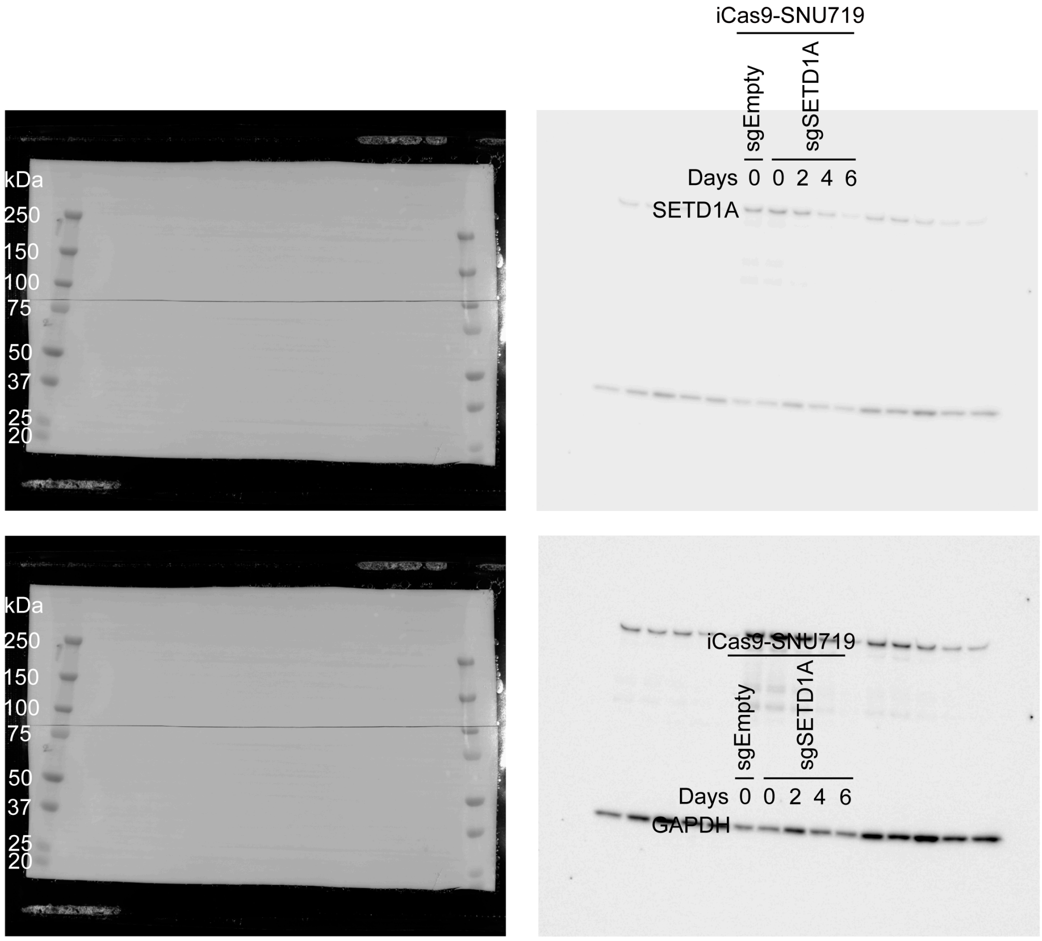
iCas9-SNU719
sgEmpty
sgSETD1A
Days 0 0 2 4 6
SETD1A
kDa
250
150
100
75
50
37
25
20
kDa
250
150
100
75
50
37
25
20
iCas9-SNU719
sgEmpty
sgSETD1A
Days 0 0 2 4 6
GAPDH

Fig. S2B

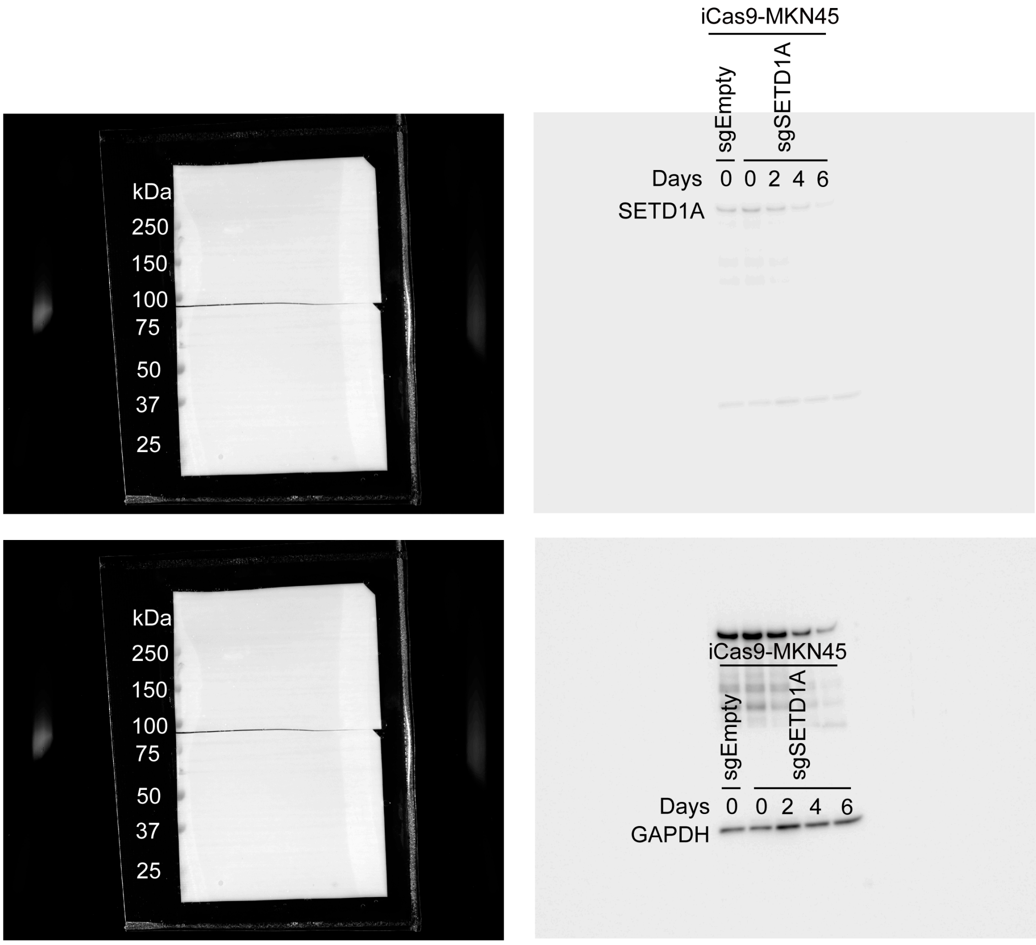

kDa
250
150
100
75
50
37
25
iCas9-MKN45
sgEmpty
sgSETD1A
Days 0 0 2 4 6
SETD1A
kDa
250
150
100
75
50
37
25
iCas9-MKN45
sgEmpty
sgSETD1A
Days 0 0 2 4 6
GAPDH

Fig. S3B

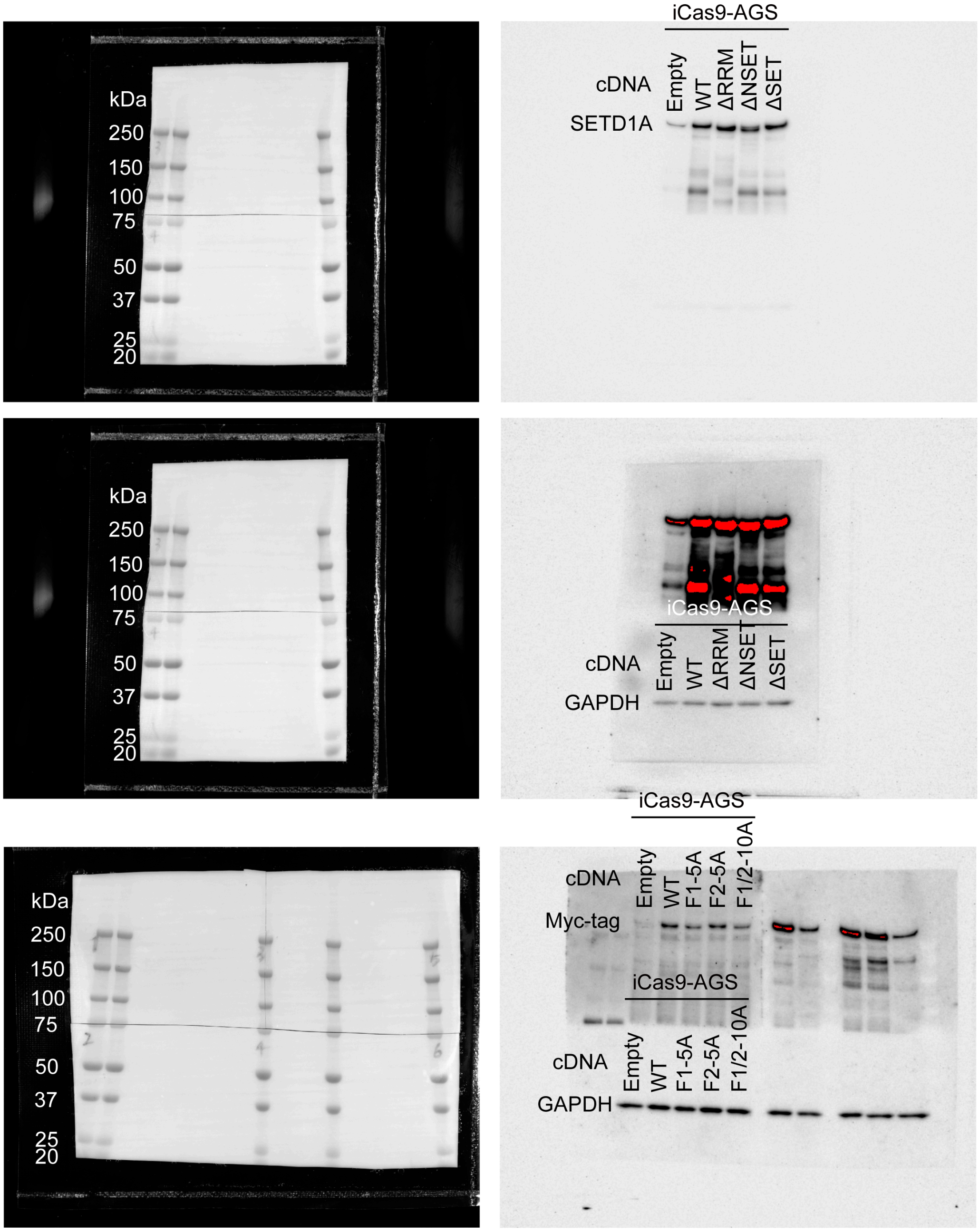
kDa
250
150
100
75
50
37
25
20
iCas9-AGS
cDNA
Empty
WT
ΔRRM
ΔNSET
ΔSET
SETD1A
GAPDH
Myc-tag
F1-5A
F2-5A
F1/2-10A

Fig. S5F

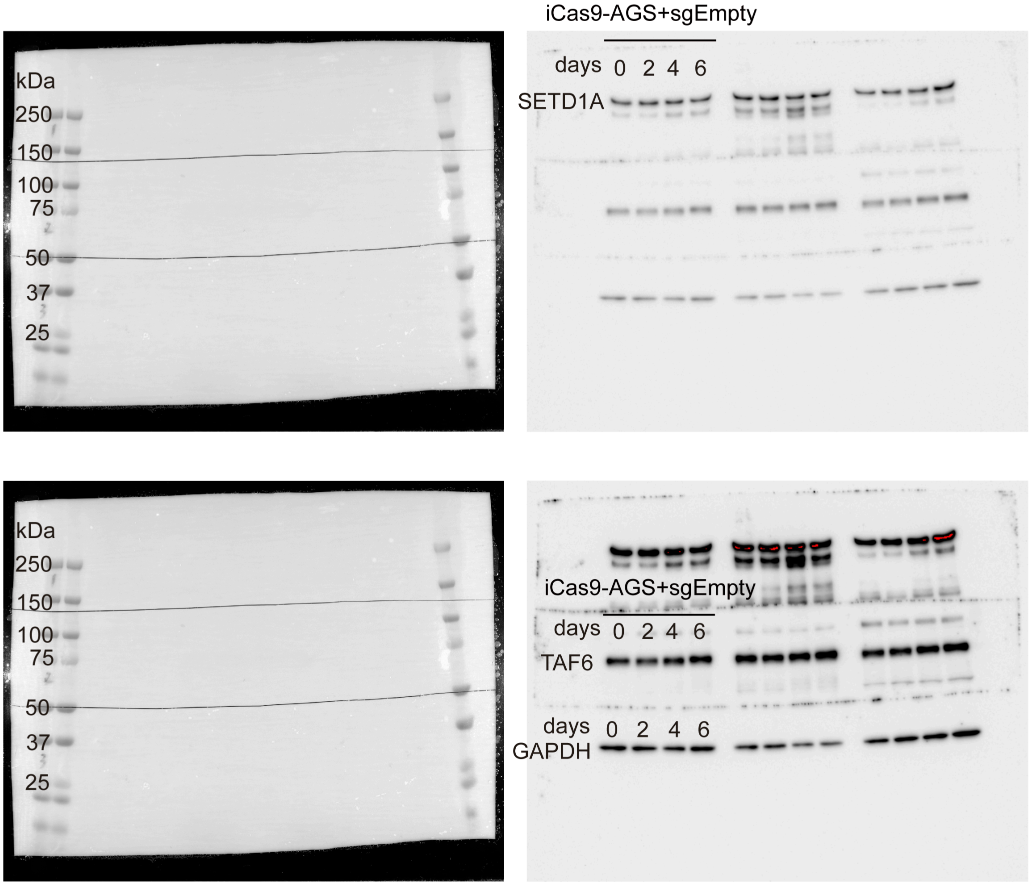
iCas9-AGS+sgEmpty
days 0 2 4 6
SETD1A
kDa
250
150
100
75
50
37
25
kDa
250
150
100
75
50
37
25
iCas9-AGS+sgEmpty
days 0 2 4 6
TAF6
days 0 2 4 6
GAPDH

Fig. S5F

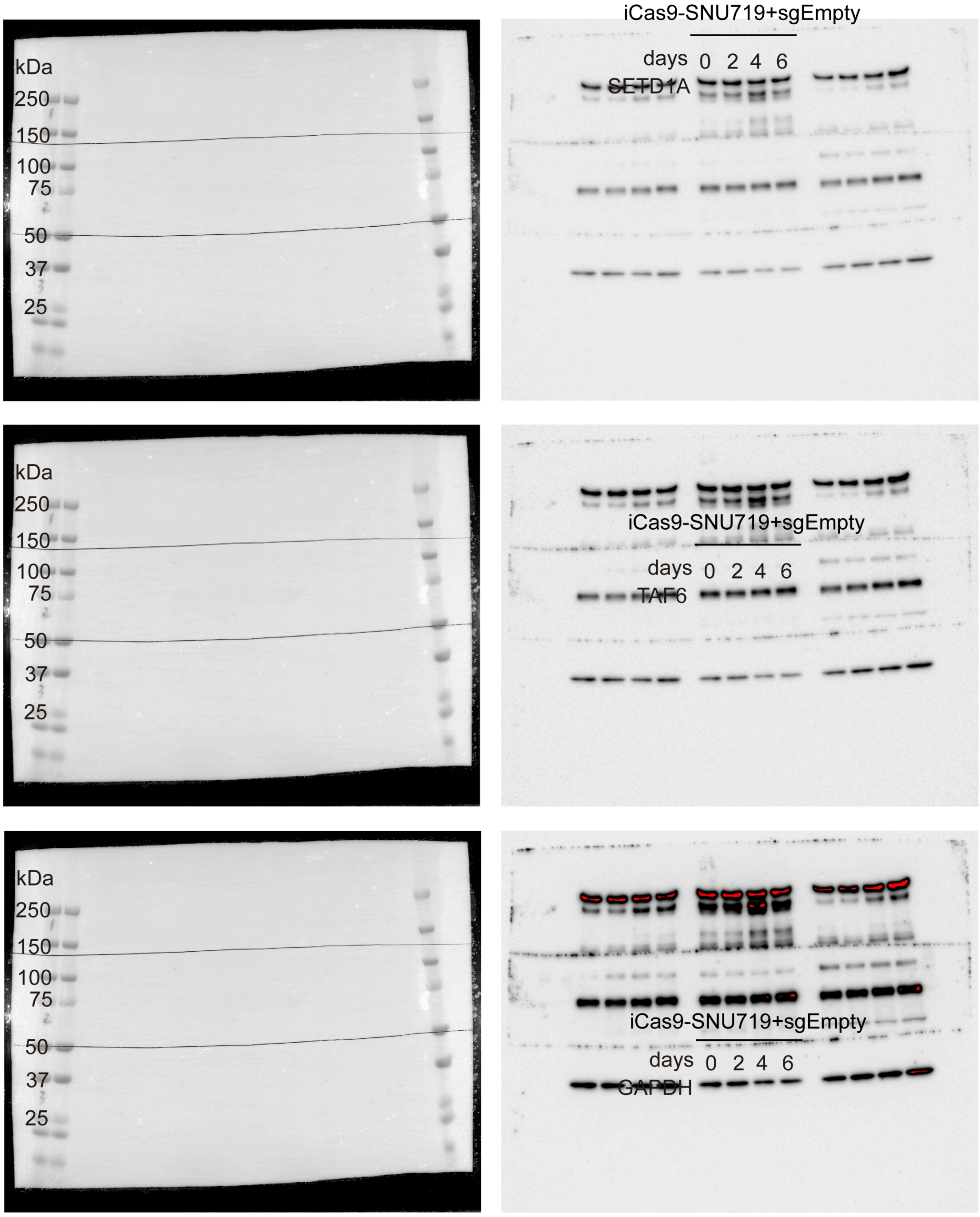

kDa
250
150
100
75
50
37
25
iCas9-SNU719+sgEmpty
days 0 2 4 6
SETD1A
kDa
250
150
100
75
50
37
25
iCas9-SNU719+sgEmpty
days 0 2 4 6
TAF6
kDa
250
150
100
75
50
37
25
iCas9-SNU719+sgEmpty
days 0 2 4 6
GAPDH

Fig. S5F

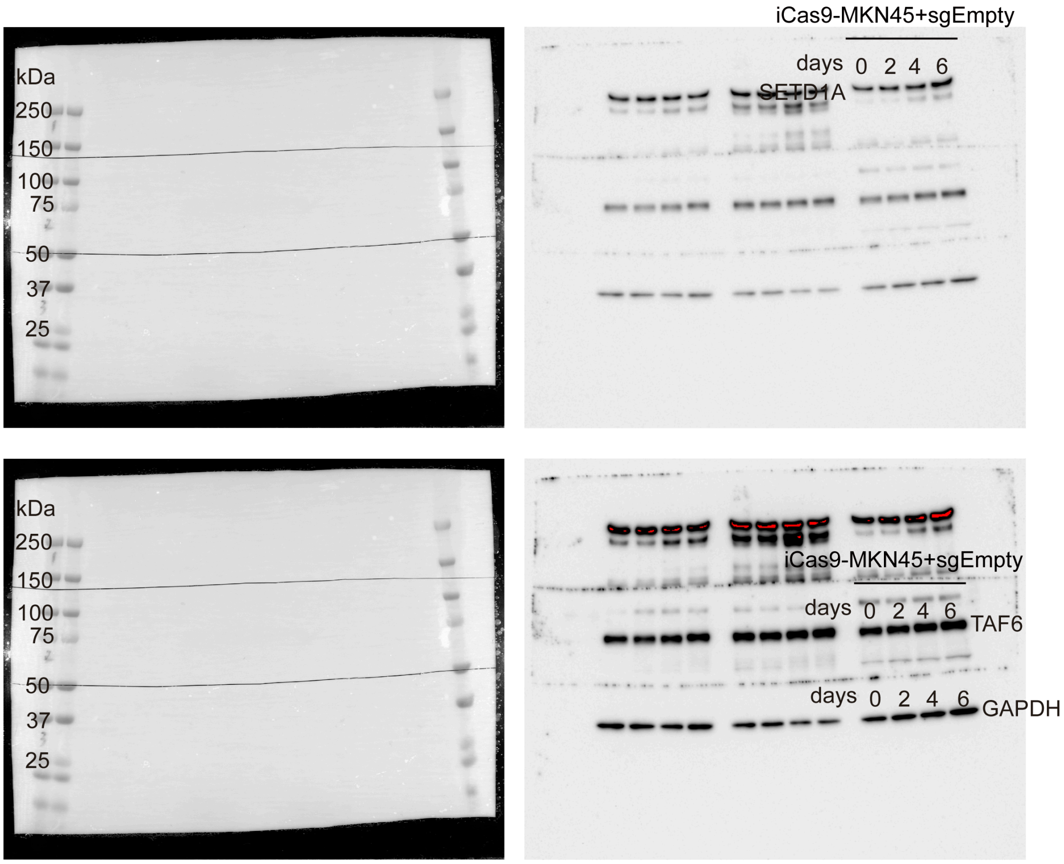

kDa
250
150
100
75
50
37
25
iCas9-MKN45+sgEmpty
days 0 2 4 6
SETD1A
kDa
250
150
100
75
50
37
25
iCas9-MKN45+sgEmpty
days 0 2 4 6
TAF6
days 0 2 4 6
GAPDH

Fig. S8D

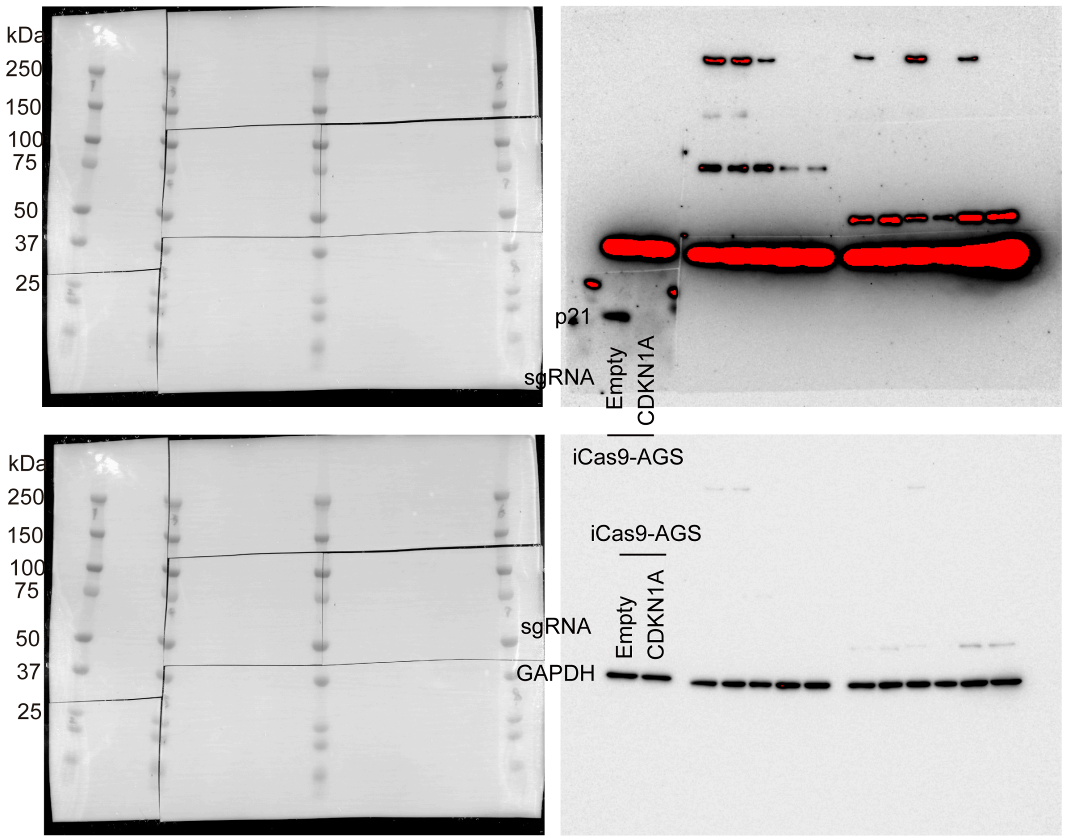
kDa
250
150
100
75
50
37
25
p21
sgRNA
Empty
CDKN1A
iCas9-AGS
kDa
250
150
100
75
50
37
25
iCas9-AGS
sgRNA
Empty
CDKN1A
GAPDH

Fig. S8E

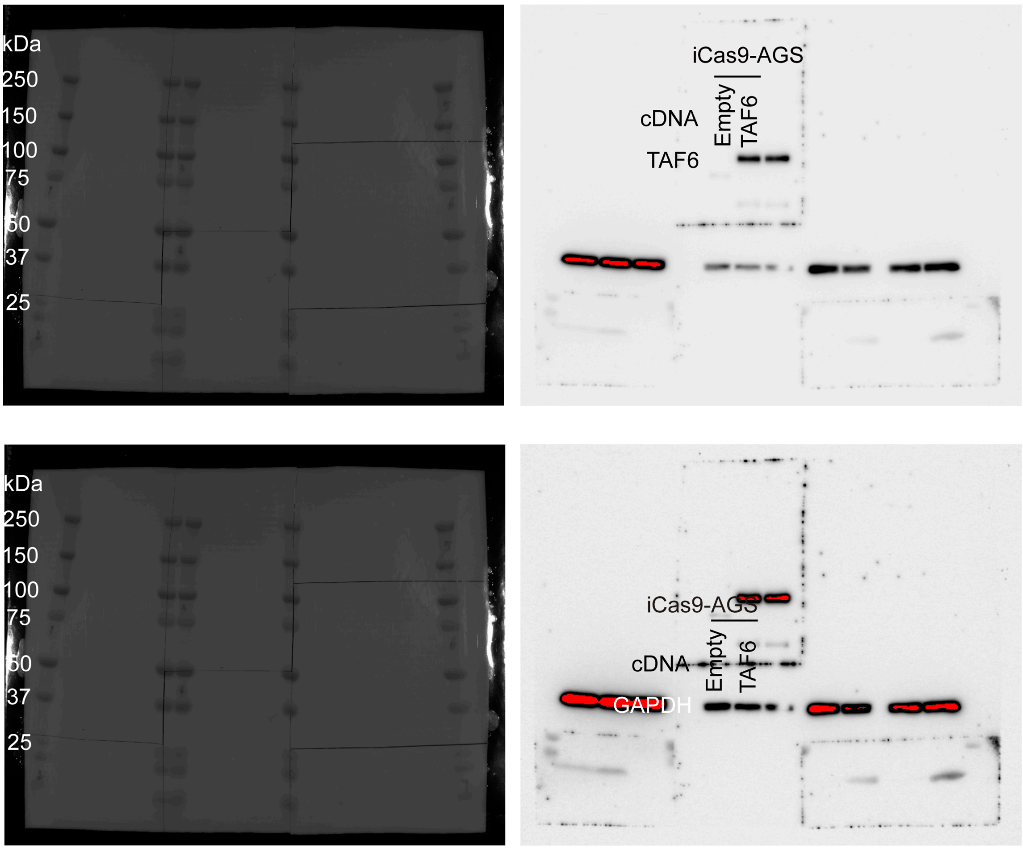
kDa
250
150
100
75
50
37
25
iCas9-AGS
cDNA
Empty
TAF6
TAF6
kDa
250
150
100
75
50
37
25
iCas9-AGS
cDNA
Empty
TAF6
GAPDH

Fig. S9A

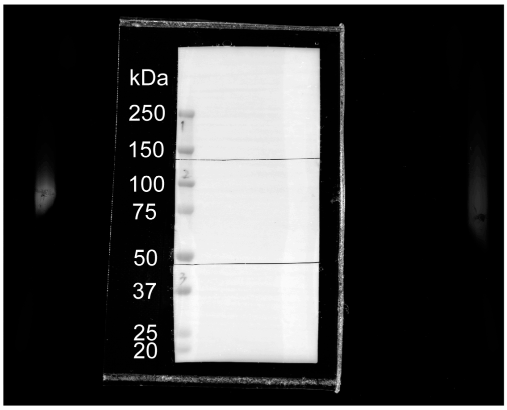

kDa
250
150
100
75
50
37
25
20

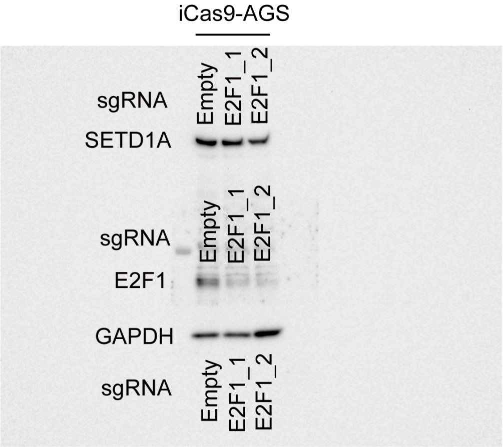

iCas9-AGS
sgRNA
Empty
E2F1_1
E2F1_2
SETD1A
sgRNA
Empty
E2F1_1
E2F1_2
E2F1
GAPDH
sgRNA
Empty
E2F1_1
E2F1_2
